# AXIN1 boosts antiviral response through IRF3 stabilization and induced phase separation

**DOI:** 10.1038/s41392-024-01978-y

**Published:** 2024-10-09

**Authors:** Dan-Ling Dai, Chu Xie, Lan-Yi Zhong, Shang-Xin Liu, Le-Le Zhang, Hua Zhang, Xing-Ping Wu, Zhou-Ming Wu, Kexin Kang, Yan Li, Ya-Meng Sun, Tian-Liang Xia, Chen-Song Zhang, Ao Zhang, Ming Shi, Cong Sun, Mei-Ling Chen, Ge-Xin Zhao, Guo-Long Bu, Yuan-Tao Liu, Kui-Yuan Huang, Zheng Zhao, Shu-Xin Li, Xiao-Yong Zhang, Yun-Fei Yuan, Shi-Jun Wen, Lingqiang Zhang, Bin-Kui Li, Qian Zhong, Mu-Sheng Zeng

**Affiliations:** 1 Department of Experimental Research, State Key Laboratory of Oncology in South China, Guangdong Provincial Clinical Research Center for Cancer, Sun Yat-sen University Cancer Center, Guangzhou, P. R. China; 2 Shenzhen Key Laboratory of Systems Medicine for inflammatory diseases, School of Medicine, Shenzhen Campus of Sun Yat-Sen University, Sun Yat-sen University, Shenzhen, Guangdong, P. R. China; 3 Department of Clinical Laboratory, State Key Laboratory of Oncology in South China, Collaborative Innovation Center for Cancer Medicine, Guangdong Key Laboratory of Nasopharyngeal Carcinoma Diagnosis and Therapy, Sun Yat-sen University Cancer Center, Guangzhou, P. R. China; 4 The State Key Laboratory of Membrane Biology, Tsinghua-Peking Center for Life Sciences, School of Life Sciences, Tsinghua University, Beijing, P. R. China; 5 Department of Pathology, State Key Laboratory of Oncology in South China, Guangdong Provincial Clinical Research Center for Cancer, Sun Yat-sen University Cancer Center, Guangzhou, P. R. China; 6 State Key Laboratory of Cellular Stress Biology, School of Life Sciences, Xiamen University, Xiamen, Fujian, P. R. China; 7 Department of Liver Surgery, State Key Laboratory of Oncology in South China, Guangdong Provincial Clinical Research Center for Cancer, Sun Yat-sen University Cancer Center, Guangzhou, P. R. China; 8 Department of Nuclear medicine, State Key Laboratory of Oncology in South China, Guangdong Provincial Clinical Research Center for Cancer, Sun Yat-sen University Cancer Center, Guangzhou, P. R. China; 9 State Key Laboratory of Organ Failure Research, Guangdong Provincial Key Laboratory of Viral Hepatitis Research, Department of Infectious Diseases, Nanfang Hospital, Southern Medical University, Guangzhou, P. R. China; 10 Medicinal Synthetic Chemistry Center, Department of Experimental Research, State Key Laboratory of Oncology in South China, Guangdong Provincial Clinical Research Center for Cancer, Sun Yat-sen University Cancer Center, Guangzhou, P. R. China; 11 State Key Laboratory of Medical Proteomics, National Center for Protein Sciences (Beijing), Beijing Institute of Lifeomics, Beijing, P. R. China

**Keywords:** Innate immunity, Microbiology

## Abstract

Axis inhibition protein 1 (AXIN1), a scaffold protein interacting with various critical molecules, plays a vital role in determining cell fate. However, its impact on the antiviral innate immune response remains largely unknown. Here, we identify that AXIN1 acts as an effective regulator of antiviral innate immunity against both DNA and RNA virus infections. In the resting state, AXIN1 maintains the stability of the transcription factor interferon regulatory factor 3 (IRF3) by preventing p62-mediated autophagic degradation of IRF3. This is achieved by recruiting ubiquitin-specific peptidase 35 (USP35), which removes lysine (K) 48-linked ubiquitination at IRF3 K366. Upon virus infection, AXIN1 undergoes a phase separation triggered by phosphorylated TANK-binding kinase 1 (TBK1). This leads to increased phosphorylation of IRF3 and a boost in IFN-I production. Moreover, KYA1797K, a small molecule that binds to the AXIN1 RGS domain, enhances the AXIN1-IRF3 interaction and promotes the elimination of various highly pathogenic viruses. Clinically, patients with HBV-associated hepatocellular carcinoma (HCC) who show reduced AXIN1 expression in pericarcinoma tissues have low overall and disease-free survival rates, as well as higher HBV levels in their blood. Overall, our findings reveal how AXIN1 regulates IRF3 signaling and phase separation-mediated antiviral immune responses, underscoring the potential of the AXIN1 agonist KYA1797K as an effective antiviral agent.

## Introduction

Highly pathogenic viruses such as hepatitis B virus (HBV) and severe acute respiratory syndrome coronavirus 2 (SARS-CoV-2) pose significant threats to global health.^1,2^ The innate immune system, particularly the type I interferon (IFN-I) signaling cascade, serves as the body’s primary defense mechanism.^3–5^ IFN-I signaling is crucial for antiviral responses and bridging innate and adaptive immunity.^6,7^ Upon viral infection, pattern-recognition receptors (PRRs) detect pathogen-associated molecular patterns (PAMPs), triggering downstream pathways to induce IFN-stimulated genes (ISGs), curb viral replication, and activate adaptive immune responses.^8,9^ Key players in this process include cyclic GMP-AMP synthase (cGAS)-stimulator of interferon genes (STING)-interferon regulatory factor 3 (IRF3) and retinoic acid-inducible gene I (RIG-I)-mitochondrial antiviral-signaling protein (MAVS)-IRF3.^3,10–13^

In particular, the crucial transcriptional factor IRF3 functions as a common downstream mediator of DNA and RNA viruses-induced antiviral IFN-I signaling, and its protein level directly affects IFN-I response level and the innate immune homeostasis.^10,14^ Previous studies have focused on post-infection regulation of IRF3 stability.^15–17^ However, regulation on resting-state protein level of key factors in innate immune signaling is also vital, which could be crucial for potentiating rapid and efficient response against any possible offenders,^18,19^ but it is less well understood in the case of IRF3. Moreover, modulating innate immune responses can offer broad-spectrum antiviral effects, and potentially overcome the limitations posed by rapidly evolving pathogens and the emergence of drug-resistant strains.^20,21^ Thus, the discovery and functional elucidation of novel players within this cascade have profound implications, not just for our understanding of immune responses, but also for the development of innovative therapeutic strategies.

Regulator of G-protein signaling (RGS) proteins, a large family of proteins with a common RGS domain, primarily regulate G-protein signaling and downstream G protein-coupled receptors (GPCRs) signaling for chemokines in both innate and adaptive immunity.^22,23^ Abnormal RGS protein expression can disrupt B lymphocyte and T lymphocyte function in autoimmune disorders, lymphoid malignancies, and allergic responses.^24,25^ As a member of RGS proteins, axis inhibition protein 1 (AXIN1) has been known to mediate multiple signaling pathways, such as Wnt/β-catenin,^26^ transforming growth factor β (TGF-β),^27^ c-Jun N-terminal kinase (JNK)- mitogen-activated protein kinase kinase kinase (MAP3K)^28^ and liver kinase B1 (LKB1)- AMP-activated protein kinase (AMPK),^29^ and involved in various developmental processes including axis formation,^30^ neural development,^31^ tumor suppression^32^ and metabolism regulation.^29,33^ However, its role in the modulation of immune responses, particularly in the innate immune system, is a relatively new avenue of exploration. Recent insights into liquid-liquid phase separation (LLPS) suggest a fundamental role in organizing cellular biochemistry, including immune responses.^34–36^ Previous reports have revealed that AXIN1 underwent LLPS while it was overexpressed in cells or added with PEG8000 in vitro, and was associated with regulation of the Wnt signaling.^37^ However, it is unclear whether LLPS as a regulatory mechanism in immune signaling plays a role in the non-canonical role of AXIN1 in innate immunity.

In this study, we show that AXIN1 stabilizes IRF3 in the resting state and undergoes phase separation upon viral infection to promote IFN-I production. The AXIN1 agonist KYA1797K further enhances IFN-I expression and provides protection against various virus infections in vitro and in vivo. Our findings highlight the AXIN1–IRF3 axis as a key regulator of innate immune signaling and a promising target for immune therapy.

## Results

### AXIN1 serves as a positive antiviral innate immunity regulator against both DNA and RNA virus infections

**Fig. 1:**
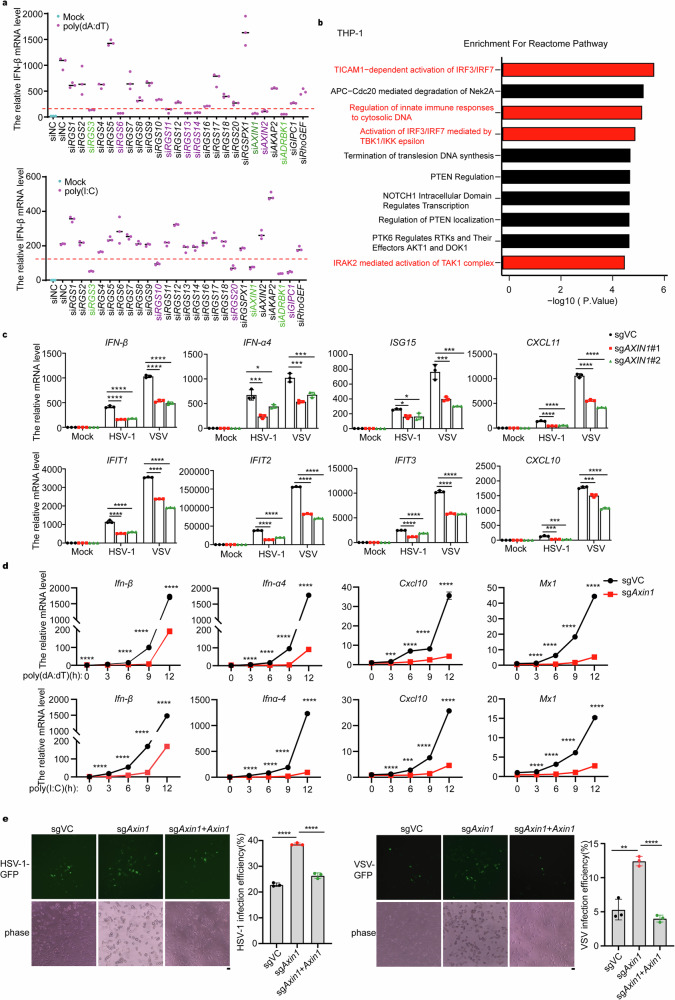
AXIN1 positively regulates DNA and RNA virus-induced IFN-I signaling. **a** qPCR analysis of *IFN-β* mRNA in THP-1 cells transfected with siRNAs targeting 25 RGS family members followed by treatment with 2 μg/ml poly(dA:dT) or poly(I:C) (*n* = 3). In the graph, genes are marked as purple or green if their knockdown resulted in significantly impaired IFN-β expression after either or both of poly(dA:dT) or poly(I:C) induction. **b** Pathway enrichment analysis of differentially expressed proteins (*p* < 0.05) between vector control (sgVC) (*n* = 2) or *AXIN1*-KO (sg*AXIN1*) THP-1 cells (*n* = 3) performed using REACTOME website tools (https://reactome.org/). The top 10 enriched pathways are shown. **c** qPCR analysis of *IFN-β* and *IFN-α4* mRNAs and indicated *ISGs* in vector control (sgVC) or *AXIN1*-KO (sg*AXIN1*#1, sg*AXIN1*#2) THP-1 cells infected with or without HSV-1 (MOI = 0.5) or VSV (MOI = 1) for 12 h (*n* = 3). **d** qPCR analysis of *Ifn-β* and *Ifn-α4* mRNAs and indicated ISGs in vector control (sgVC) or *Axin1*-KO (sg*Axin1*) MEFs treated with or without 2 μg/mL poly(dA:dT) or poly(I:C) for the indicated time periods (*n* = 3). **e** Microscopic imaging and flow cytometry analysis of sgVC, sg*Axin1*, and Axin1-reconstituted sg*Axin1* MEFs infected with HSV-1-GFP (MOI = 0.2) or VSV-GFP (MOI = 0.05) for 16 h (*n* = 3). The scale bar indicates a length of 100 μm. Data are shown as mean ± standard deviation (S.D.) and represent three independent experiments. Statistical analyses were performed using (**c**, **e**) one-way ANOVA with multiple-comparison test or (**d**) Student’s two-tailed unpaired *t*-test. *P < 0.05; **P < 0.01; ***P < 0.001; ****P < 0.0001. AXIN1 axis inhibition protein 1, HSV-1 herpes simplex virus 1, IFN interferon, IRF3 interferon regulatory factor 3, ISGs interferon-stimulated genes, KO knockout, MEFs mouse embryonic fibroblasts, MOI multiplicity of infection, RGS regulator of G-protein signaling, siRNA small interfering RNA, VC vector control, VSV vesicular stomatitis virus

To uncover the potential role of RGS family members in IFN-I signaling, we conducted a comprehensive screening using small interfering RNAs (siRNAs) to individually downregulate the expression of 25 RGS family members and subsequently examined the induction of IFN-β expression upon treatment with poly(dA:dT) or poly(I:C). As shown, *RGS3*, *AXIN1* or *ADRBK1* knockdown resulted in a robust inhibition of IFN-β expression (Fig. 1a). Furthermore, knockdown of *AXIN1* using three different siRNAs exhibited the most pronounced inhibition of IFN-β expression induced by herpes simplex virus 1 (HSV-1) and vesicular stomatitis virus (VSV) infection (Supplementary Fig. 1a). Encouraged by these findings, we generated *AXIN1*-knockout (*AXIN1*-KO) cells and confirmed the knockout efficiency by immunoblotting (Supplementary Fig. 1b). AXIN2, the homolog of AXIN1, was also examined, and its protein expression remained unaffected by *AXIN1*-knockout. To probe the potential role of AXIN1, we performed proteomic mass spectrometry analysis in *AXIN1*-KO and control THP-1 cells. As expected, the genes with differential protein expression levels between *AXIN1*-KO and control cells, were mainly enriched in innate immune pathways (Fig. 1b). We therefore focused on validating the role of AXIN1 in regulating the IFN-I signaling. Consistently, *AXIN1* deficiency significantly impaired the expression levels of IFN-β, IFN-α4, and representative interferon-stimulated genes (ISGs) induced by HSV-1 and VSV infection or poly(dA:dT) and poly(I:C) treatment (Fig. 1c, d and Supplementary Fig. 1c). These results provide strong evidence that AXIN1 positively regulates the IFN-I signaling pathway.

We then investigated the role of AXIN1 in regulating viral infection. AXIN1- deficient MEFs and THP-1 cells were infected with the recombinant virus HSV-1-GFP or VSV-GFP followed by flow cytometry to measure the infection efficiency. Strikingly, we observed that AXIN1 deficiency markedly facilitated HSV-1 and VSV infection, while Axin1 reconstitution in *Axin1*-KO cells rescued the increased infection of HSV-1 and VSV (Fig. 1e and Supplementary Fig. 1d). Collectively, these compelling findings firmly establish the pivotal role of AXIN1 in promoting innate immune responses against DNA and RNA viral infections.

### AXIN1 deficiency decreases total IRF3 protein level in the resting-state

We then investigated the mechanism through which AXIN1 positively regulates innate immune responses. Considering that the TANK-binding kinase 1 (TBK1) and IRF3 are common downstream DNA and RNA virus-induced IFN-I signaling mediators,^11^ we ectopically expressed AXIN1 along with either TBK1 or IRF3-5D (an active IRF3 mutant) in HEK293T cells and examined their effects on IFN-β promoter-driven luciferase activity. The results showed that AXIN1 overexpression promoted TBK1- and IRF3-5D-mediated IFN-β promoter activation, suggesting that AXIN1 acts as a downstream TBK1 effector (Supplementary Fig. 2a). In contrast, IFN-β treatment induced similar levels of CXCL10 and IFIT1 in wild-type (WT) and *AXIN1*-KO cells (Supplementary Fig. 2b), indicating that AXIN1 acts IFN-β upstream. Thus, AXIN1 may function at the level of IRF3, which acts TBK1 downstream and IFN-β upstream.

**Fig. 2:**
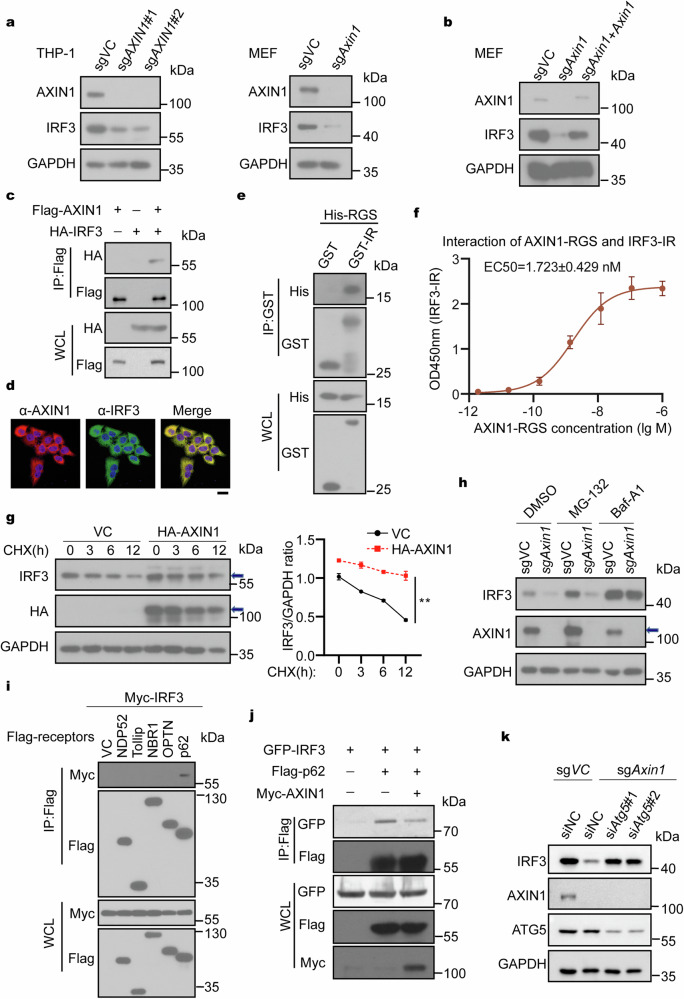
AXIN1 interacts with and stabilizes IRF3. **a** Immunoblotting assay of total IRF3 protein in sgVC or sg*AXIN1* MEFs and THP-1 cells. **b** Immunoblotting assay of IRF3 in Axin1-reconstituted sg*Axin1* MEFs. **c** HEK293T cell lysates co-transfected with HA-IRF3- and Flag-AXIN1-expressing plasmids or vector were immunoprecipitated with anti-Flag beads and immunoblotted with the indicated antibodies. **d** HeLa cells were immunostained with anti-AXIN1 and anti-IRF3, followed by microscopy analysis. Scale bar represents 20 μm. **e** The purified His-AXIN1-RGS was immunoprecipitated with GST or GST-IRF3-IR and immunoblotted with the indicated antibodies. **f** ELISA analysis of the binding affinity of purified GST-IRF3-IR with His-AXIN1-RGS (*n* = 3). **g** Immunoblotting assay (left) and relative quantitation (right) of IRF3 in BEAS-2B cells transfected with a plasmid expressing HA-AXIN1 or VC followed by the treatment with 1 μg/mL CHX for the indicated time periods. The arrows indicate the specific proteins of interest. **h** Immunoblotting assay of IRF3 in sgVC or sg*Axin1* MEFs treated with DMSO, 1 μM MG-132, or 1 μM Baf-A1 for 12 h. The arrow indicates the AXIN1 protein. **i** HEK293T cells were transfected with plasmids expressing Myc-IRF3 and indicated Flag-tagged cargo receptors followed by immunoprecipitation and immunoblotting with the indicated antibodies. **j** HEK293T cells were transfected with GFP-IRF3, Flag-p62, and Myc-AXIN1 or the related vector control and then subjected to the immunoprecipitation and immunoblot with the designated antibodies. **k** Immunoblotting assay of IRF3 in sg*Axin1* MEFs transfected with RNAi#*Atg5* (si*Atg5*#1, si*Atg5*#2). Data are shown as mean ± standard deviation (S.D.) and represent three independent experiments. Statistical analyses were performed using (**g**) two-way ANOVA comparison test. *P < 0.05; **P < 0.01; ***P < 0.001; ****P < 0.0001. ELISA enzyme-linked immunosorbent assay, IFN interferon, IgG Immunoglobulin G, IRF3 interferon regulatory factor 3, WCL whole cell lysate

Upon virus infection, IRF3 undergoes serial phosphorylation processes followed by dimerization and nuclear translocation to initiate downstream signaling.^38^ We therefore detected phosphorylated and total IRF3 levels in virus-infected and control cells. Unexpectedly, *AXIN1-*KO decreased not only the phosphorylated IRF3 upon virus infection, but also the total IRF3 at the resting-state (Supplementary Fig. 2c). Consistently, the total IRF3 level at the resting-state decreased significantly in the *Axin1*-KO MEFs and THP-1 cells (Fig. 2a). These results suggested that AXIN1 was important for maintaining the total IRF3 level in the resting-state, which serves as a reservoir for efficient phosphorylated IRF3 generation upon virus infection and determines the response level upon confronting virus challenge. Consistently, the IRF3 protein level increased in AXIN1-overexpressing cells (Supplementary Fig. 2d). Furthermore, Axin1 reconstitution in *Axin1*-KO cells rescued the decreased IRF3 protein level (Fig. 2b). Moreover, the IRF3 mRNA levels in control and *Axin1*-KO MEFs were comparable, suggesting that AXIN1 regulates the IRF3 protein level (Supplementary Fig. 2e). Importantly, the previous mass spectrometry proteomic analysis also demonstrated a 75% decrease in IRF3 protein level in AXIN1-deficient THP-1 cells compared to control cells (Fig. 1b and Supplementary Fig. 2f–g).

To further determine whether IRF3 acts downstream of AXIN1 in the innate immune response, *IRF3*-knockout cells were transfected with AXIN1-expressing or control plasmids, followed by analysis of IFN-β expression after poly(I:C) treatment. The results showed that AXIN1 overexpression could not promote IFN-β expression in *IRF3*-knockout cells (Supplementary Fig. 2h), supporting that AXIN1 was an upstream IRF3 regulator. Together, these results suggest that AXIN1 facilitates IFN-I signaling by maintaining the IRF3 protein level in the resting-state, thus allowing a rapid and robust innate immune response against infecting virus.

### AXIN1 interacts with IRF3

As AXIN1 is a scaffold protein exerting various functions by interacting with different protein partners,^39,40^ we next investigated whether AXIN1 interacted with IRF3. We observed that HA-IRF3 was co-immunoprecipitated with Flag-AXIN1 in cells expressing both proteins (Fig. 2c). Furthermore, the immunostaining and confocal microscopy results indicated that AXIN1 and IRF3 were mainly co-localized in the cytoplasm (Fig. 2d). These results together reveal a physical interaction between AXIN1 and IRF3.

To determine the regions involved in AXIN1–IRF3 interaction, we first conducted a co-immunoprecipitation experiment using several AXIN1 truncation mutants and found that AXIN1 N-terminus (aa 1–211), which includes the RGS domain, is required and sufficient for the interaction (Supplementary Fig. 3a). Conversely, we used IRF3 truncation mutants for co-immunoprecipitation assay and found that the IR domain of IRF3 was indispensable for the interaction between AXIN1 and IRF3 (Supplementary Fig. 3b). Since the RGS domain is the main part of the AXIN1 N-terminus, we investigated whether AXIN1 RGS domain directly interacted with IRF3 IR domain. We performed immunoprecipitation and enzyme-linked immunosorbent assay (ELISA) in cell-free system using purified His-tagged AXIN1 RGS domain and GST-tagged IRF3 IR domain. As expected, the AXIN1 RGS directly interacted with the IRF3 IR (Fig. 2e). ELISA showed an EC50 of 1.723±0.429 nM between AXIN1 RGS domain and IRF3 IR domain, suggesting a strong interaction affinity (Fig. 2f). Orthogonally, AXIN1 RGS and IRF3 IR domains were co-localized in the cytoplasm, as revealed by immunofluorescence staining (Supplementary Fig. 3c). Together, these results show that AXIN1 directly interacts with IRF3 and that AXIN1 RGS domain and IRF3 IR domain are crucial for this interaction.

### AXIN1 blocks p62-mediated autophagic IRF3 degradation

We next investigated the mechanism underlying the AXIN1-mediated IRF3 protein increase. We determined the IRF3 protein half-life in BEAS-2B cells treated with cycloheximide (CHX), a protein translation inhibitor^41^ and observed that AXIN1 overexpression prolonged IRF3 half-life (Fig. 2g). Previous studies have suggested that proteasome and autophagy pathways are major protein degradation systems in eukaryotic cells.^42^ To identify the degradation system that dominantly regulates the AXIN1 knockout-mediated IRF3 degradation, we treated *Axin1*-KO cells with inhibitors specifically targeting these two degradation pathways and found that the autophagic degradation inhibitor Baf-A1, but not proteasome degradation inhibitor MG-132, largely restored the IRF3 decrease caused by *Axin1* KO (Fig. 2h), suggesting that IRF3 mainly undergoes autophagic degradation in the absence of AXIN1.

It is well accepted that cargo receptors deliver ubiquitinated proteins to autophagosomes for selective degradation.^43^ To investigate the cargo receptor responsible for autophagic IRF3 degradation, we performed immunoprecipitation assay using HEK293T cells transfected with plasmids expressing IRF3 and different cargo receptors. We found that IRF3 interacted only with p62 and further confirmed the interaction between the endogenous IRF3 and p62 (Fig. 2i and Supplementary Fig. 4a). These results indicated that autophagic IRF3 degradation was mediated by the cargo receptor p62. Additionally, the interaction between IRF3 and p62 was enhanced in cells treated with autophagy inducing reagents such as Earle’s balanced salt solution (EBSS) and Rapamycin (Rapa), further supporting the role of p62 as an autophagic IRF3 degradation mediator (Supplementary Fig. 4b,c). Consistently, AXIN1 overexpression inhibited the interaction between p62 and IRF3 (Fig. 2j). Moreover, IRF3 degradation was almost completely abrogated in *Axin1*-silenced cells when ATG5, a downstream mediator for p62-mediated autophagic degradation, was knocked down by siRNA (Fig. 2k). In summary, these findings suggest that AXIN1 inhibits p62-mediated autophagic IRF3 degradation.

### AXIN1 inhibits K48-linked IRF3 ubiquitination at K366

The p62 ubiquitin-associated (UBA) domain is essential for recruiting ubiquitinated substrates for degradation.^44^ We therefore tested the ability of a truncated p62 without its UBA domain to interact with IRF3 and found that UBA-truncated p62 could not interact with IRF3 (Supplementary Fig. 5a), suggesting that IRF3 degradation was mediated by its ubiquitin chains. AXIN1 overexpression decreased the lysine (K) 48-linked IRF3 ubiquitination. Neither K27- nor K63-linked polyubiquitination appeared to be a major IRF3 modification and AXIN1 overexpression did not change that (Supplementary Fig. 5b). To identify the K48-linked IRF3 ubiquitination sites, the lysine in five potential IRF3 ubiquitination sites predicted by the UbPred program^45^ were individually mutated to arginine (R). Subsequent immunoprecipitation results showed that the K48-linked IRF3-K366R ubiquitination reduced most significantly among these mutants (Supplementary Fig. 5c). Moreover, the interaction between p62 and IRF3-K366R was most attenuated than that with WT IRF3 or other mutants (Supplementary Fig. 5d). In summary, our results demonstrate that K48-linked IRF3 ubiquitination at K366 is a signal for p62 recognition and subsequent autophagic degradation.

### AXIN1 enhances the interaction between USP35 and IRF3

Given that AXIN1 is not a deubiquitinating enzyme (DUB), we hypothesized that AXIN1 might function as a scaffold to recruit certain DUBs to remove the K48-linked IRF3 ubiquitin chains. To identify the IRF3-targeting DUB, we employed an immunoprecipitation-based approach to screen for DUBs that interact with both AXIN1 and IRF3, and successfully identified five potential candidate DUBs (Supplementary Fig. 6a). Interestingly, only the interaction between IRF3 and ubiquitin specific peptidase 35 (USP35) was enhanced when AXIN1 was overexpressed (Supplementary Fig. 6b). Consistently, *AXIN1* knockdown attenuated the interaction between IRF3 and USP35, but not the interaction between IRF3 and any other USPs (Supplementary Fig. 6c). These results indicate that AXIN1 potentially functions as a crucial scaffold to facilitate the interaction between USP35 and IRF3.

Subsequently, we investigated the role of USP35 in regulating IRF3 stability. AXIN1 overexpression inhibited the K48-linked ubiquitination, which was further attenuated by USP35 augmentation, revealing that USP35 may be recruited by AXIN1 to remove the K48-linked IRF3 polyubiquitin moieties (Supplementary Fig. 6d,e). Consistently, *USP35* knockdown decreased the IRF3 level (Supplementary Fig. 6f) and the expression level of IFN-β, CXCL10 and ISG15 upon HSV-1 or VSV infection (Supplementary Fig. 6g), suggesting the importance of USP35 in maintaining IRF3 level and potentiating innate immune response. These results indicate that AXIN1 might recruit USP35 to remove the K48-linked IRF3 ubiquitination, thereby maintaining IRF3 stability and facilitating innate immune response.

### Liquid-liquid phase separation of AXIN1 induced by virus infection facilitates the phosphorylation of IRF3

**Fig. 3:**
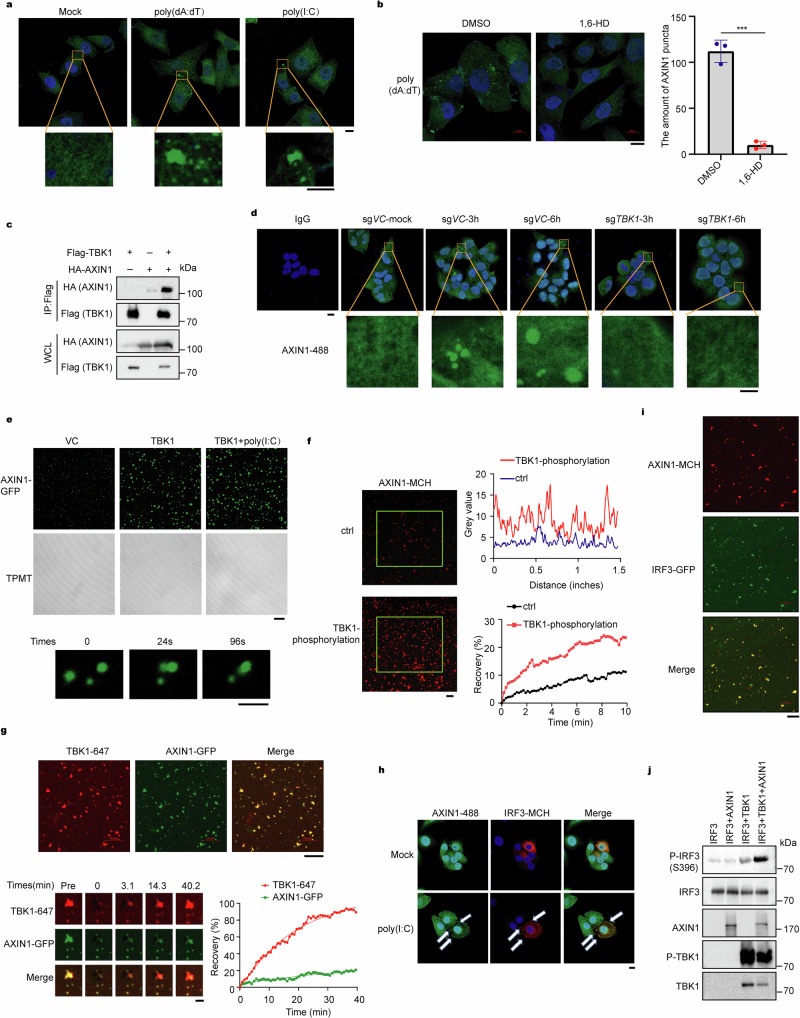
Phosphorylated TBK1 promotes the LLPS of AXIN1. **a** Immunostaining analysis of endogenous AXIN1 in HeLa cells treated by poly (dA:dT) (1.5 μg/μl) and poly (I:C) (1.5 μg/μl) for 3 h. Scale bar represents 10 μm in the top original image, and it denotes 5 μm in the magnified image. **b** Immunostaining analysis of endogenous AXIN1 in HeLa cells stimulated by poly (dA:dT) (1.5 μg/μl) followed by treatment with DMSO or 1,6-hexanediol (1,6-HD) for 10 min. Scale bar represents 10 μm. **c** Immunoprecipitation analysis of Flag-TBK1 and HA-AXIN1 in HEK293T cells. **d** Immunostaining analysis of endogenous AXIN1 in sgVC or sg*TBK1* HeLa cells treated with or without poly (I:C). Scale bar represents 10 μm in the top original image, and it denotes 2 μm in the magnified image. **e** LLPS of purified AXIN1-GFP (5 μM) treated by TBK1 which was pulled down from cell lysates in HEK293T cells induced with or without poly (I:C) and droplet fusion assay. Scale bar represents 10 μm in the top original image, and it denotes 2 μm in the magnified image. **f** The fluorescence image of purified AXIN1-MCH (5 μM) treated with or without phosphorylated TBK1 in the presence of 2% PEG8000, followed by the intensity line analysis and FRAP analysis. Scale bar represents 10 μm. **g** The fluorescence image and FRAP analysis of representative AXIN1-GFP (5 μM) and 647-labeled TBK1 in the mixed droplets. Scale bar represents 10 μm in the top original image, and it denotes 2 μm in the magnified image. **h** The fluorescence image analysis of endogenous AXIN1 and exogenous IRF3-MCH in HeLa cells treated by poly (I:C) (1.5 μg/μl) for 3 h. The arrow indicates the AXINl co-localized with punctate IRF3. Scale bar represents 10 μm. **i** Droplet formation of AXIN1-MCH and IRF3-GFP (5 μM) treated with phosphorylated TBK1 in the presence of 2% PEG8000. Scale bar represents 10 μm. **j** Purified IRF3-MCH was treated with or without phosphorylated TBK1 or MBP-AXIN1-GFP followed by immunoblotting with indicated antibody. VC vector control, CTRL control, MCH mcherry

AXIN1 maintains the basal expression level of IRF3 in the resting state, enabling its rapid phosphorylation for efficient IFN-I production upon virus infection. However, the specific mechanism by which AXIN1 regulates IRF3 signaling in response to virus infection requires further investigation. Intriguingly, immunofluorescence imaging revealed the robust aggregation of AXIN1 into substantial puncta in the cytoplasm upon nucleic acid sensing or virus infection (Fig. 3a and Supplementary Fig. 7a). Importantly, the treatment with 1,6-hexanediol (1,6-HD), a compound that putatively disrupts weak hydrophobic interactions,^46^ significantly inhibited the AXIN1 puncta formation (Fig. 3b). These results suggest that AXIN1 undergoes liquid-liquid phase separation (LLPS) upon virus infection in cells.

Considering that the protein kinase TBK1 is the common regulator in the IFN-I signaling induced by both DNA and RNA viruses and phosphorylation has been identified as a trigger for LLPS,^47^ we hypothesized that AXIN1 could undergo LLPS in the presence of TBK1-mediated phosphorylation. We found that AXIN1 interacted with TBK1 and colocalized in the cytoplasm (Fig. 3c and Supplementary Fig. 7b). Consistent with our hypothesis, we observed that knockout of *TBK1* significantly impaired the LLPS of AXIN1 upon poly(I:C) treatment (Fig. 3d). Furthermore, the Coomassie Brilliant Blue staining results showed that overexpression of TBK1 increased the molecule weight of AXIN1, indicating that AXIN1 may be phosphorylated by TBK1 (Supplementary Fig. 7c). Next, due to the lack of antibody specifically targeting phosphorylated AXIN1, we performed phosphorylation mass spectrometry analysis and confirmed that AXIN1 was phosphorylated by TBK1 at several serine/threonine sites (Supplementary Fig. 7d). These results indicated that AXIN1 was phosphorylated by TBK1, which potentially facilitates the AXIN1 LLPS formation.

Next, we conducted in vitro experiments to assess whether TBK1 phosphorylation could promote the LLPS of AXIN1. Maltose-binding protein (MBP)-tagged AXIN1-GFP or -MCH proteins were purified and then treated with TBK1, which was pulled down from cell lysate. Notably, more and larger AXIN1-GFP droplets appeared upon the addition of TBK1 from TBK1-overexpressing cells lysates upon poly(I:C) treatment (Fig. 3e), suggesting that TBK1 phosphorylation could promote the LLPS of AXIN1. Additionally, we observed the two distinct AXIN1 puncta fused into a larger droplet, indicating the dynamic nature and liquid-like properties of AXIN1 puncta in vitro (Fig. 3e). Subsequently, we purified TBK1 to conduct in vitro phosphorylation assay and the phosphorylated TBK1 was added into the purified AXIN1-MCH proteins in the presence of 2% PEG8000. As shown, more AXIN1-MCH droplets occurred upon TBK1 phosphorylation treatment, and the fluorescence intensity greatly increased in the TBK1-phosphorylation group compared to the control (Fig. 3f). Meanwhile, the addition of phosphorylated TBK1 also significantly enhanced the fluorescence recovery speed of AXIN1-MCH droplets after photobleaching (Fig. 3f).

To further investigate the role of TBK1 in the formation of AXIN1 LLPS, 647-labeled TBK1 was added to AXIN1-GFP proteins in the presence of 2% PEG8000, and compared with 647-labeled TBK1 or AXIN1-GFP alone in the same conditions. We found that 647-labled TBK1 alone did not form phase-separated droplets, while AXIN1-GFP could form droplets in 2% PEG8000 (Supplementary Fig. 7e). Upon further addition of 647-labeled TBK1, the droplets became significantly larger and more numerous (Supplementary Fig. 7e). Additionally, in the mixed 647-labeled TBK1 and AXIN1-GFP group, the fluorescence signals of TBK1-647 and AXIN1-GFP highly overlapped, indicating that both proteins were incorporated into the same droplets (Fig. 3g). Moreover, the fluorescence recovery after photobleaching (FRAP) analysis demonstrated that the recovery rate of fluorescence in TBK1-647 was notably more rapid compared to that observed in AXIN1-GFP (Fig. 3g). It has been reported that clients diffused much faster within droplets than scaffolds.^48^ This suggests that AXIN1 acts as the scaffold in LLPS, recruiting TBK1 as the ‘client’ into the droplets. As a client, TBK1 exhibited faster fluorescence recovery due to its quicker exchange rate between the inside and outside of the droplets. These results confirmed that TBK1 phosphorylation could promote the LLPS of AXIN1 and TBK1 was recruited into the phase.

Subsequently, we sought to determine whether IRF3 is recruited to the AXIN1 condensates. As expected, AXIN1 condensates co-localized with punctate IRF3 structures in the cytoplasm upon poly(I:C) treatment (Fig. 3h). Interestingly, immunoprecipitation assay revealed that the interaction between AXIN1 and IRF3 was enhanced following both HSV-1 and VSV infections (Supplementary Fig. 7f). Consistently, AXIN1 and IRF3 were present in the same droplets in the presence of TBK1 phosphorylation (Fig. 3i). Moreover, we found that the phase separation of AXIN1 could promote the phosphorylation of IRF3 (Fig. 3j). These results revealed that AXIN1 may act as the scaffold to recruit IRF3 into the droplets and promoted the phosphorylation of IRF3.

These data suggests that AXIN1 undergoes LLPS upon virus infection, with TBK1 and IRF3 being recruited into the AXIN1 droplets to efficiently promote the phosphorylation of IRF3.

### AXIN1-deficient mice are susceptible to virus infection

**Fig. 4:**
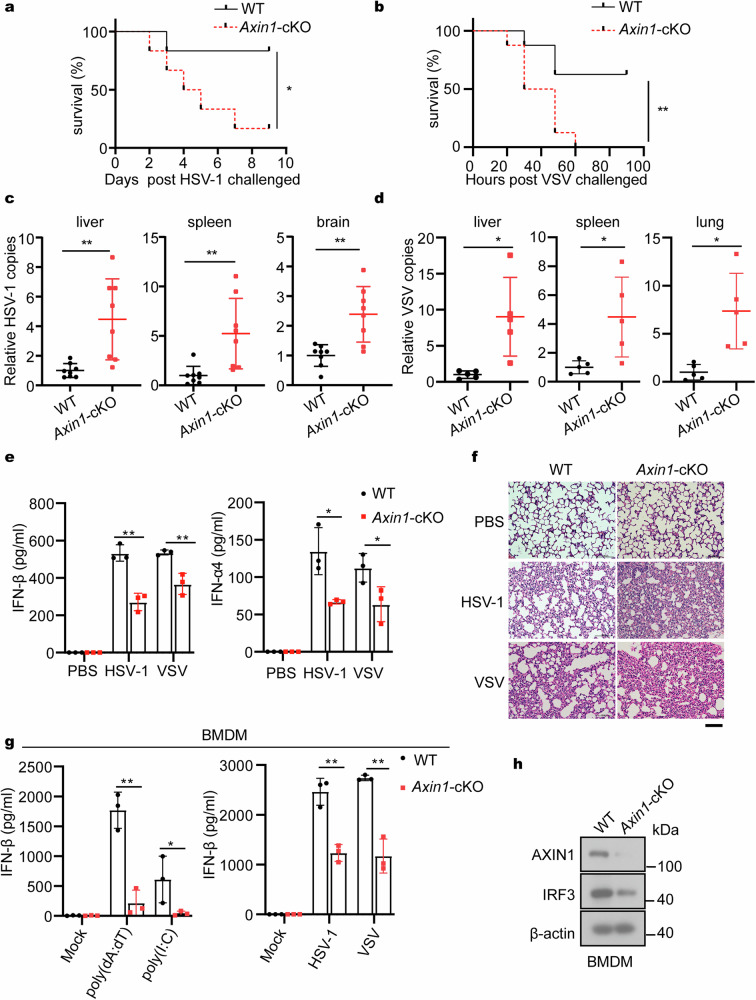
Axin1 conditional knockout mice are more susceptible to DNA and RNA virus infections and produce less IFN-I. **a**, **b** Survival analysis of WT or *Axin1*-cKO mice intravenously injected with (**a**) 8×10^6^ pfu/g HSV-1 (*n* = 6) or (**b**) 1 × 10^7^ pfu/g VSV (*n* = 8). **c** qPCR analysis of the virus DNA copy number in liver, spleen, and brain of WT or *Axin1*-cKO mice intravenously injected with 1×10^6^ pfu/g HSV-1 for 72 h (*n* = 8). **d** qPCR analysis of the virus RNA copy number in liver, spleen, and lung of WT or *Axin1*-cKO mice intravenously injected with 1×10^6^ pfu/g VSV for 48 h (*n* = 5). **e** ELISA analysis of serum IFN-β and IFN-α4 in WT or *Axin1*-cKO mice intravenously injected with 1 × 10^6^ pfu/g HSV-1 or VSV for 24 h (*n* = 3). **f** H&E staining showing damages in the lungs of WT or *Axin1*-cKO mice intravenously injected with 1 × 10^6^ pfu/g HSV-1 or VSV for 48 h, scale bar represent 100 μm. **g** ELISA analysis of IFN-β in the supernatant of BMDMs from WT or *Axin1*-cKO mice infected with HSV-1 (MOI = 5) and VSV (MOI = 5) or treated with 3 μg/mL poly(dA:dT) and poly(I:C) for 16 h (*n* = 3). **h** Immunoblotting assay of AXIN1 and IRF3 in the VC and *Axin1-*cKO BMDMs. Data are shown as mean ± standard deviation (S.D.) and represent two independent experiments. Statistical analyses were performed using (**a**, **b**) Log-rank (Mantel-Cox) test, (**e**, **g**) Student’s two-tailed unpaired *t*-test, or (**c**, **d**) Student’s two-tailed unpaired *t*-test with Welch’s correction. *P < 0.05; **P < 0.01; ***P < 0.001; ****P < 0.0001. WT wild-type, cKO conditional knockout, BMDM bone-marrow-derived macrophage, ELISA enzyme-linked immunosorbent assay, H&E hematoxylin and eosin, MOI multiplicity of infection

Since AXIN1 plays an important role in the antiviral response in vitro and whole-body *Axin1* KO causes embryonic lethality,^30,49^ we investigated the function of AXIN1 in vivo using mice with myeloid lineage-specific *Axin1* deletion (*Axin1*^*fl/fl*^*Lyz2-cre*^*+*^, hereafter designated as “*Axin1-cKO*” mice). The *Axin1* KO efficiency in bone-marrow-derived macrophages (BMDMs) of *Axin1-cKO* mice was verified (Supplementary Fig. 8). *Axin1-cKO* mice exhibited increased susceptibility to both HSV-1 or VSV infection than WT mice upon intravenous injection, as evidenced by the lower survival rate and period (Fig. 4a, b), suggesting that AXIN1 protects mice against both DNA and RNA virus infections. Consistent with this, the number of HSV-1 genomic DNA copies increased in *Axin1-cKO* mice liver, brain, and spleen (Fig. 4c). Similarly, VSV replication in *Axin1-cKO* mice spleen, liver, and lung was significantly enhanced than that in WT mice (Fig. 4d).

We additionally infected WT and *Axin1-cKO* mice with HSV-1 and VSV to assess the serum levels of IFN-β and IFN-α4 and found that AXIN1 deficiency resulted in reduced levels of these cytokines (Fig. 4e). We also observed more inflammatory cell infiltration in the *Axin1-cKO* mice lung than that in the WT mice lung after HSV-1 and VSV infection (Fig. 4f). As shown, genetic *Axin1* ablation significantly inhibited IFN-β secretion upon HSV-1 and VSV infections or poly(dA:dT) and poly(I:C) treatments (Fig. 4g). Additionally, the IRF3 level in BMDM cells isolated from *Axin1-cKO* mice was lower than that of WT (Fig. 4h). In summary, conditional *Axin1* KO impairs IFN-I secretion, increasing the susceptibility to both DNA and RNA virus. Thus, AXIN1 is crucial for host defense against both DNA and RNA virus in vivo.

### KYA1797K, a small molecule AXIN1 agonist, promotes antiviral innate immunity against highly pathogenic virus, including SARS-CoV-2

**Fig. 5:**
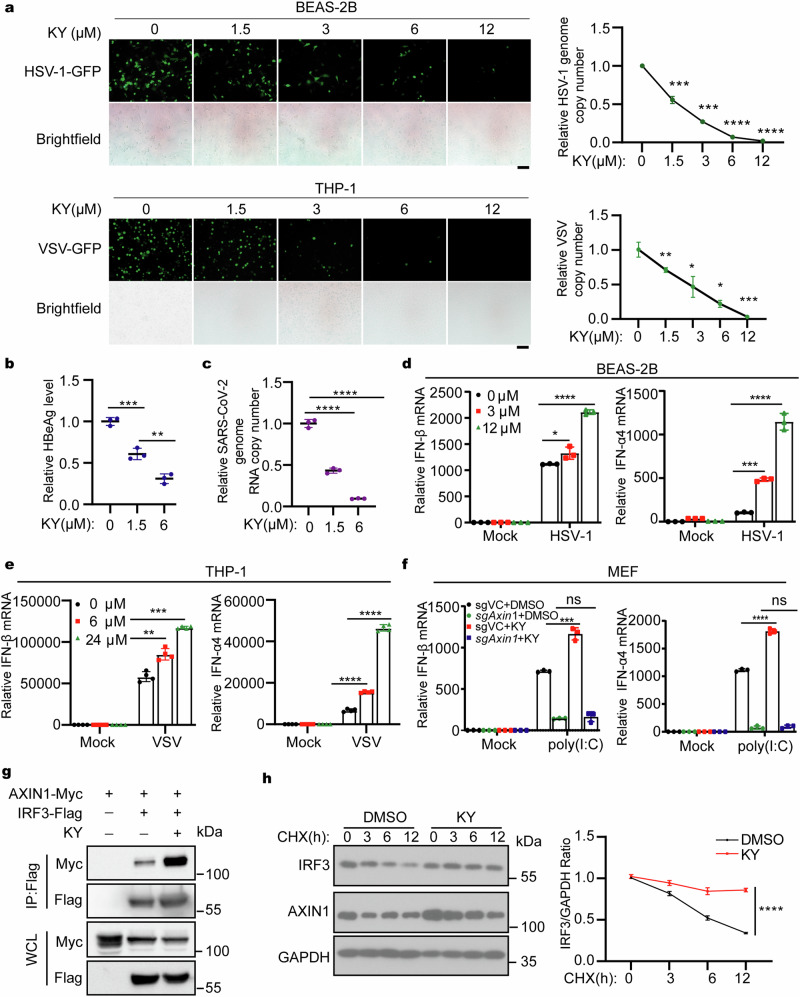
A small molecule AXIN1 agonist inhibits HSV-1 and VSV infections via enhancing IFN-I expression. **a** Microscopy imaging (left) or qPCR analysis (right) indicating the HSV-1 or VSV amount in BEAS-2B (top) and THP-1 cells (bottom) treated with the indicated KYA1797K concentrations followed by HSV-1-GFP (MOI = 0.1; *n* = 3) or VSV-GFP (MOI = 0.1; *n* = 4) infection for 16 h. Scale bar represents 100 μm. **b** ELISA analysis of HBeAg in the supernatant of Hepa1-6 cells treated with the indicated KYA1797K concentrations followed by transfection with HBV plasmids for 48 h (*n* = 3). **c** qPCR analysis of the SARS-CoV-2 copy number in the supernatant of BEAS-2B cells treated with different KYA1797K concentrations (*n* = 3). **d**, **e** qPCR analysis of IFN-β and IFN-α4 expression in (**d**) BEAS-2B (*n* = 3) and (**e**) THP-1 cells (*n* = 4) treated with KYA1797K as indicated concentrations and infected with HSV-1 (MOI = 1) or VSV (MOI = 1) for 12 h. **f** qPCR analysis of IFN-β and IFN-α4 expression in VC or *Axin1*-KO MEFs treated with DMSO or KYA1797K (6 μM) followed by transfection with 2 μg/mL poly(I:C) for 6 h (*n* = 3). **g** HEK293T cells were transfected with plasmids expressing Myc-AXIN1 and Flag-IRF3 and treated with 12 μM KYA1797K for 12 h followed by immunoprecipitation with anti-Flag beads and immunoblotting with the indicated antibodies. **h** Immunoblotting assay (left) and relative quantitation (right) of IRF3 in BEAS-2B cells exposed to 12 μM KYA1797K for 12 h, followed by addition of 1 μg/mL CHX for different time periods. Data are shown as mean ± standard deviation (S.D.) and represent three independent experiments. Statistical analyses were performed using (**a**–**f**) one-way ANOVA with Tukey’s multiple-comparison test or (**h**) two-way ANOVA comparison test. *P < 0.05; **P < 0.01; ***P < 0.001; ****P < 0.0001; ns not significant. Baf-A1 bafilomycin A1, CHX cycloheximide, DMSO dimethyl sulfoxide, MOI multiplicity of infection, SARS-CoV-2 severe acute respiratory syndrome coronavirus 2, KY KYA1797K

Due to the crucial role of AXIN1 in the antiviral immune response, we further investigated whether we could modulate the innate immune response through targeting AXIN1. KYA1797K is a small molecule that binds to AXIN1 RGS domain^50^ and we examined the effect of KYA1797K treatment on antiviral immunity. Encouragingly, KYA1797K treatment remarkably inhibited HSV-1 and VSV infection of THP-1 and BEAS-2B cells dose-dependently (Fig. 5a). Importantly, KYA1797K also suppressed the HBV and SARS-CoV-2 replication in Hepa1-6 and BEAS-2B cells, respectively, which supports the pan-antiviral effect of KYA1797K (Fig. 5b, c). Consistently, KYA1797K administration promoted HSV-1 and VSV infection- or poly(dA:dT) and poly(I:C) treatment-induced IFN-β and IFN-α4 production dose-dependently (Fig. 5d, e and Supplementary Fig. 9a).

To explore the mechanism underlying immune-enhancing and antiviral effects of KYA1797K, we examined IFN-I production after poly(I:C) stimulation in AXIN1-deficient or control MEFs with or without KYA1797K treatment. Notably, KYA1797K treatment significantly enhanced IFN-β production in control cells, but not AXIN1-deficient MEFs (Fig. 5f). This supported that KYA1797K elevated IFN production mainly through targeting AXIN1. Moreover, in *IRF3*-knockout HeLa cells, the effect of KYA1797K to promote IFN-β production was nearly completely abrogated, suggesting its dependence on IRF3-mediating IFN-β signaling (Supplementary Fig. 9b). We then investigated whether USP35 involves in the immune-enhancing and antiviral effects of KYA1797K. *USP35* knockdown in THP-1 significantly inhibited IFN-β response upon stimulation with poly (dA:dT) or poly (I:C) in both KYA1797K-treated and control groups (Supplementary Fig. 9c). We further examined HSV-1 infection or VSV infection efficiency in *USP35* knockdown or control THP-1 cells with or without KYA1797K treatment. Microscopy imaging and flow cytometry results showed that *USP35* knockdown promoted HSV-1 and VSV infection, and while KYA1797K was able to suppress the infection of HSV-1 and VSV, this effect largely reduced in USP35 knockdown cells (Supplementary Fig. 9d,e). Collectively, these findings suggest that the pro-innate immune and antiviral effects of KYA1797K depend on its effect on the AXIN1-IRF3 axis.

We further investigated whether KYA1797K modulated AXIN1–IRF3 interaction and found that KYA1797K enhanced the interaction between AXIN1 and IRF3, which was consistent with KYA1797K-mediated IFN-β production enhancement (Fig. 5g). Furthermore, KYA1797K treatment prolonged IRF3 half-life (Fig. 5h). In conclusion, KYA1797K is a potential innate immune agonist that prominently promotes IFN-I expression and inhibits several viral infections in vitro, suggesting that targeting the AXIN1–IRF3 axis could be a novel strategy to develop antiviral drugs.

### KYA1797K protects mice from both DNA and RNA virus infections

**Fig. 6:**
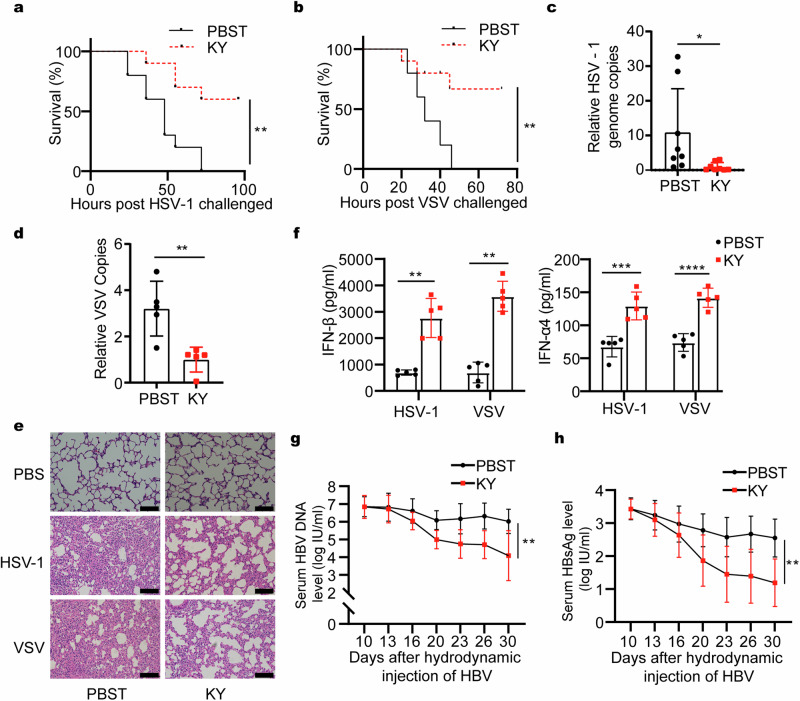
In vivo KYA1797K treatment protects mice from various viral infections. **a**, **b** Survival analysis of C57BL/6 mice intraperitoneally injected with 25 mg/kg KYA1797K, followed by intravenous 1 × 10^7^ pfu/g (**a**) HSV-1 or (**b**) VSV injection (*n* = 10). **c**, **d** qPCR analysis of DNA or RNA copy number in the brain or liver of C57BL/6 mice intraperitoneally injected with KYA1797K followed by intravenous 1 × 10^6^ pfu/g (**c**) HSV-1 (*n* = 8) or (**d**) VSV (*n* = 5) injection for 48 h. **e** H&E analysis of the lung of C57BL/6 mice intraperitoneally injected with 25 mg/kg KYA1797K followed by intravenous 1 × 10^6^ pfu/g HSV-1 or VSV injection for 48 h. Scale bar indicates 100 μm. **f** ELISA analysis of serum IFN-β and IFN-α4 of C57BL/6 mice intraperitoneally injected with 25 mg/kg KYA1797K followed by intravenous 1 × 10^6^ pfu/g HSV-1 or VSV injection for 24 h (*n* = 5). **g**, **h** qPCR analysis of (**g**) HBV DNA copy number and (**h**) ELISA analysis of HBsAg in the serum of male C57BL/6 mice hydrodynamically injected with pAAV/HBV1.2 plasmid for 10 days followed by intraperitoneal KYA1797K injection (*n* = 10). Data are shown as mean ± standard deviation (S.D.) and represent two independent experiments. Statistical analyses were performed using (**a**, **b**) Log-rank (Mantel–Cox) test, (**g**, **h**) two-way ANOVA with Sidak’s multiple-comparison test, or (**c**, **d**, **f**) Student’s two-tailed unpaired *t*-test with Welch’s correction. *P < 0.05; **P < 0.01; ***P < 0.001; ****P < 0.0001; ns not significant. ELISA enzyme-linked immunosorbent assay, H&E hematoxylin and eosin, HBV hepatitis B virus, HSV-1 herpes simplex virus 1, IFN interferon, VSV vesicular stomatitis virus, KY KYA1797K

We then examined whether KYA1797K protects against DNA or RNA virus infection in vivo. We injected mice intraperitoneally with KYA1797K or vehicle 1 day before HSV-1 and VSV challenges and then treated them daily with KYA1797K after virus challenge. KYA1797K treatment protected the mice from HSV-1 and VSV infection, verified by the increased survival rate and duration of the KYA1797K-treated mice (Fig. 6a, b). Histological examination indicated less lung damage, accompanied with reduced HSV-1 and VSV loads in the brain and liver of KYA1797K-treated mice (Fig. 6c–e). In addition, KYA1797K treatment increased the serum IFN-β and IFN-α4 levels (Fig. 6f). In response to HSV-1 and VSV infection, BMDMs isolated from KYA1797K-treated mice showed increased IFN-β level in the supernatant (Supplementary Fig. 10a). Moreover, we established a chronic HBV infection mouse model through hydrodynamic HBV1.2 plasmid injection and then intraperitoneally injected these mice with KYA1797K or the control solution PBST every two days. HBV DNA and HBsAg levels reduced in KYA1797K-treated mice serum, indicating that KYA1797K inhibited the HBV replication in vivo (Fig. 6g, h). KYA1797K treatment did not significantly affect mice body weight (Supplementary Fig. 10b). Converging these findings, it is evident that KYA1797K protects mice against DNA and RNA virus infections via promoting IFN-I production in vivo.

### Increased AXIN1 expression correlates with increased IFN-β expression level and reduced HBV replication level

**Fig. 7:**
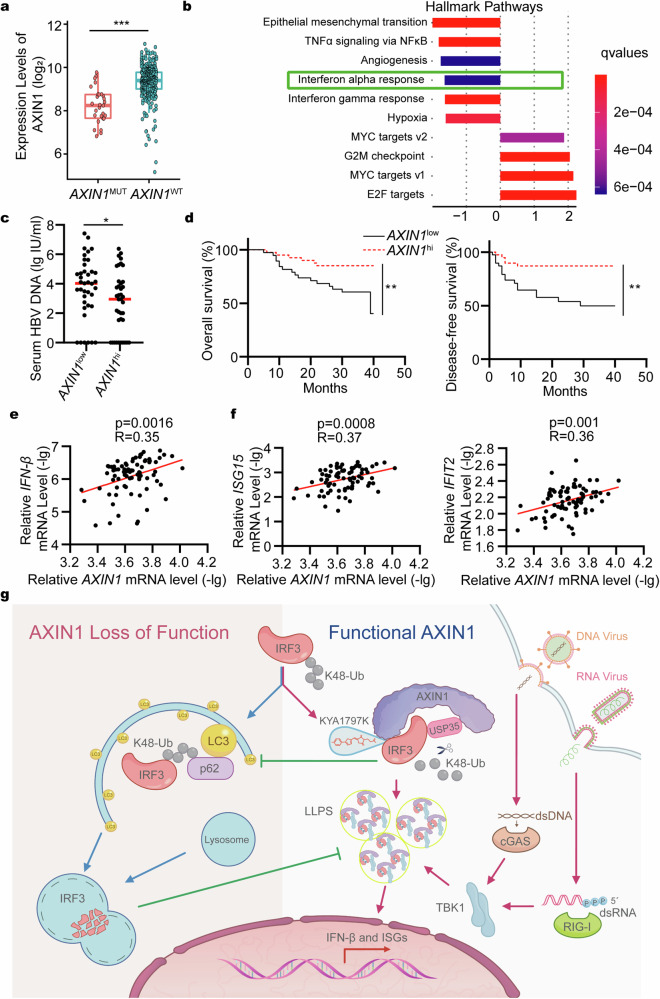
Increased AXIN1 expression correlates with increased IFN-β expression level and reduced HBV replication level. **a** AXIN1 expression level in *AXIN1*^MUT^ (*n* = 28) and *AXIN1*^WT^ (*n* = 330) human HCC samples from the TCGA LIHC dataset (https://portal.gdc.cancer.gov/). **b** GSEA of differentially expressed genes between *AXIN1*^MUT^ and *AXIN1*^WT^ groups. **c** qPCR analysis of serum HBV DNA in patients of *AXIN1*^hi^ (*n* = 39) and *AXIN1*^low^ (*n* = 39) groups. **d** The overall and progression-free survival analysis of patients in *AXIN1*^hi^ and *AXIN1*^low^ groups. **e**, **f** The correlation analysis of *AXIN1* and (**e**) *IFN-β*, (**f**) *ISG15* or *IFIT2* mRNA levels in pericarcinoma tissues (*n* = 78). **g** Schematic representation of IFN-I signaling regulated by AXIN1/IRF3 axis. The scheme was provided by Coloring Guangzhou Ltd. Data are shown as mean ± standard deviation (S.D.) and represent two independent experiments. The statistical evaluations were conducted utilizing (**a**, **c**) Student’s two-tailed unpaired *t*-test, (**d**) Log-rank (Mantel-Cox) test, or (**e**, **f**) linear regression analysis. *P < 0.05; **P < 0.01; ***P < 0.001; ****P < 0.0001. AXIN1 axis inhibition protein 1, HCC hepatocellular carcinoma, IFN interferon, IRF3 interferon regulatory factor 3, ISG interferon-stimulated genes, GSEA gene set enrichment analysis, HBV hepatitis B virus

Given the crucial role of AXIN1 in regulating the innate immune response and its close association with hepatocellular carcinoma (HCC),^51,52^ we sought to investigate whether AXIN1 dysfunction is linked to IFN-I signaling in HCC patients. We analyzed 358 human HCC samples, including 28 samples with *AXIN1* mutations, from the cancer genome atlas (TCGA) dataset that had both genetic status and RNA expression profile. AXIN1 expression level in samples harboring *AXIN1* mutations was significantly lower than that in samples harboring WT *AXIN1* (Fig. 7a). Moreover, gene set enrichment analysis (GSEA) results suggested that *AXIN1* mutations correlated with reduced IFN-I signaling (Fig. 7b). To examine AXIN1-regulated global protein expression changes, we generated *Axin1*-KO Hepa1-6 cells (Supplementary Fig. 11a), and then performed mass spectrometry proteomic analysis. REACTOME pathway enrichment analysis expectedly reported that differentially expressed proteins between *Axin1*-KO and control cells were mainly enriched in the immune regulation pathway (Supplementary Fig. 11b). These results indicate that the decreased AXIN1 expression level and *AXIN1* mutations correlate with IFN-I signaling downregulation in HCC specimens.

HBV is the main pathogen leading to HCC.^53,54^ Moreover, *AXIN1* mutations, which correlate with decreased AXIN1 expression level, appear frequently in HBV-associated HCC patients.^55^ We therefore investigated the correlation between AXIN1 expression and HBV replication in HBV-associated HCC. Increasing evidence supports that HBV replication occurs mainly in peritumoral liver tissues in HBV-associated HCC patients.^56,57^ HBV HBsAg and DNA levels in peritumoral tissues significantly correlate with their serum levels, which are important indicators for HCC prognosis.^58^ Therefore, we examined AXIN1 expression in peritumoral liver tissues and serum HBV DNA load of 78 HBV-associated HCC patients who did not receive anti-HBV therapy. Then, we divided the patients into *AXIN1*^hi^ and *AXIN1*^low^ groups using the median as cutoff and analyzed the corresponding serum HBV DNA levels of these two groups. We found that the HBV load was significantly lower in patients with increased AXIN1 expression levels in peritumoral tissues (Fig. 7c). Moreover, high AXIN1 expression in peritumoral tissues correlated with improved overall and progress-free survival (Fig. 7d). In addition, quantitative real-time PCR (qPCR) results showed that AXIN1 expression in peritumoral tissues positively associated with the upregulation of IFN-β and ISGs, including ISG15 and IFIT2 (Fig. 7e, f), suggesting that AXIN1 expression might be essential for regulating IFN-I signaling in HBV infection. These results collectively support previous reports on HBV replication as well as its negative effect on prognosis, indicating an important role of AXIN1 in HBV replication inhibition and innate immune response regulation.

Together, these results show that increased AXIN1 expression correlates with high IFN-β expression level, low HBV replication level, and improved overall and progress-free survival of HBV-associated HCC patients.

In general, we proposed a comprehensive model illustrating the role of AXIN1 in regulating innate immune response (Fig. 7g). In the resting state, functional AXIN1 recruits USP35 to cleave K48-linked IRF3 ubiquitin chains, thereby preventing IRF3 from interacting with p62 and subsequent autophagic degradation. Upon virus infection, AXIN1 is phosphorylated by TBK1 and undergoes phase separation, thereby recruiting stable IRF3 into the condensates to facilitate the phosphorylation by TBK1 and ultimately promote the IFN-I signaling. This mechanism ensures a substantial basal IRF3 level and rapid phosphorylation efficiency to activate IFN-I signaling while encountering internal or external challenges. However, in cases of AXIN1 loss-of-function due to low expression or inactivating mutations, the accumulation of K48-linked ubiquitin on IRF3 leads to its degradation, thereby hindering innate immune response activation and increasing the susceptibility to viral infections.

## Discussion

The IFN-I signaling is crucial for defending against infectious microbes, making it an attractive target for the development of broad-spectrum antiviral drug.^59–61^ Utilizing extensive siRNA screening targeting RGS family, we identified AXIN1 as a positive regulator of IRF3-mediated IFN-I signaling, and that potential agonists targeting AXIN1 could prominently enhance antiviral immunity in vitro and in vivo.

A pivotal discovery from our research is that AXIN1 maintains IRF3 protein level in the resting state. Knockout of *AXIN1* dramatically reduced resting-state IRF3 level and impaired the generation of phosphorylated IRF3 upon virus challenge, leading to ineffective IFN-I response. The basal IRF3 protein level maintained by AXIN1 is crucial as a reservoir ready for rapid and efficient activation upon viral infection. In more detail, AXIN1 constantly recruits USP35 to remove the K48-linked ubiquitination from IRF3, thereby preventing p62-mediated IRF3 autophagic degradation. Previous studies indicated that upon virus infection, IRF3 could interact with both CALCOCO2 and p62, and supposed that p62 might also play a role in controlling IRF3 stability under other conditions, and here, the regulation of p62-mediated degradation of IRF3 in the resting state is further elucidated. Our finding complements the protein level regulation network of IRF3, which underscores the importance of pre-activation regulation in innate immunity.

The discovery of AXIN1’s involvement in LLPS in response to virus infection adds a new dimension to our understanding of cellular responses to viral challenges and expands our understanding of AXIN1’s role in phase separation. Previous reports found that AXIN1 undergoes LLPS under conditions of excessive exogenous overexpression, which was shown to recruit the destruction complex to facilitate β-catenin phosphorylation by GSK3β in Wnt signaling.^37^ However, the physiological conditions that stimulate AXIN1 LLPS remain completely unknown, and here we are the first to report that AXIN1’s LLPS can be induced by viral infection. Post-translational modifications are crucial to modulate LLPS, and protein phosphorylation is one of the key regulatory mechanisms influencing LLPS, affecting LLPS through altering protein charge, modifying π bond stacking, and creating binding pockets.^62^ Our results suggest that phosphorylation of AXIN1 by phosphorylated TBK1 upon viral infection could promote AXIN1 LLPS, which then recruits additional TBK1 and IRF3 proteins into the condensate, facilitates the phosphorylation of IRF3 and enhances IFN-I production. However, the specific mechanisms and critical phosphorylation sites involved in this process remain to be further elucidated. It was reported that AXIN1 mediated LLPS involves multiple AXIN1 domains and a complex interplay of interactions. For example, the DIX domain is involved in AXIN1’s head-to-tail polymerization,^63^ influencing puncta formation, and the IDR1 domain is indispensable for AXIN1 LLPS as it is essential for GSK3β interaction and contributes to β-catenin binding.^37^ Moreover, the pathways regulated by AXIN1 are intricate, and it has been reported that GSK3β and β-catenin were involved in the antiviral innate immune response regulation.^64,65^ Whether AXIN1’s phase separation upon viral stimulation can induce crosstalk between the β-catenin pathway and the TBK1-IRF3 pathway, thereby coordinating a response to viral challenges, remains to be further investigated. This potential interaction also opens new avenues for understanding how cellular signaling pathways integrate to mount an effective antiviral response.

Given AXIN1-IRF3 axis’s crucial role in innate immune activation, we thus target this axis to modulate innate immune response against virus infection. We found the small molecule KYA1797K, which was reported to bind AXIN1 RGS domain,^50^ poses a prominent immune-enhancing property against various virus infection, including HBV and SARS-CoV-2. The KYA1797K’s ability to enhance the general anti-viral immunity may be advantageous in confronting different virus variants and drug-resistant strains. Moreover, enhancing innate immunity has been widely recognized as an important strategy for treating cancers and many drugs targeting innate immunity are undergoing clinical trials,^66–70^ and KYA1797K may also be an anti-cancer candidate. Importantly, compared with innate immune activators like Toll-like receptors or STING agonists, KYA1797K targeting AXIN1–IRF3, potentiates rather than directly eliciting IFN-I signaling, minimizing the risk of systemic inflammation response that could greatly limit clinical application.^71–73^ In addition, the correlation of AXIN1 expression with IFN-β levels and HBV replication in human HCC patients highlights the clinical relevance of our findings. The decreased AXIN1 expression in samples with AXIN1 mutations and its association with reduced IFN-I signaling in HCC underscore the importance of AXIN1 in immune regulation and its potential implications in cancer therapy.

In conclusion, this study provides substantial evidence positioning AXIN1 as a critical regulator of innate immunity, particularly in the context of viral infections. The discovery of AXIN1’s role in IFN-I signaling, its interaction with IRF3, and the novel mechanism of LLPS underscore the complexity of immune responses and open new pathways for therapeutic interventions. As we continue to explore the multifaceted roles of scaffold proteins like AXIN1 in immune regulation, their potential as targets in drug development becomes increasingly evident, offering hope for more effective treatments against a variety of infectious diseases and possibly cancer.

## Methods

### Ethics approval statements

All mouse experiments in this study were approved by the Committee on the Ethics of Animal Experiments of Sun Yat-sen University Cancer Center (ethical number: L102042020060L). All animal experiments procedures were strictly performed following the Guide for the Care and Use of Laboratory Animals of the Ministry of Science and Technology of the People’s Republic of China. Use of clinical samples, including tumor tissues, adjacent non-tumor tissues, and blood from patients with hepatocellular carcinoma, in this study was approved by the Committee on the Ethics of Sun Yat-sen University Cancer Center (Approval number: G2023-191-01), and was strictly performed in accordance with relevant international and domestic ethical guidelines and legal regulations.

### Mice

C57BL/6 *Axin1*^fl/fl^Lyz2-Cre^+^ (*Axin1-cKO*) mice with genetically ablated *Axin1* in myeloid cells were kindly gifted by Professor Sheng-Cai Lin. All mice were housed in a specific-pathogen-free (SPF) animal facility in a controlled environment (22–25 °C, 50% humidity, 12 h light/dark cycle) at Sun Yat-sen University. The genotyping primers were detailed in Supplementary Table 1. All animal experiments followed the guidelines of the Institutional Animal Care and Use Committee of Sun Yat-sen University. 8-9-week-old C57BL/6 *Axin1*^fl/fl^Lyz2-Cre^-^ (WT) and *Axin1*^fl/fl^Lyz2-Cre^+^ (*Axin1-cKO*) mice were intravenously injected with HSV-1 or VSV for further survival analysis. We obtained different tissues from each mouse for viral titer analysis at the indicated time points.

### KYA1797K treatment and viral infection

5-6-week-old C57BL/6 WT mice purchased from Guangdong Laboratory Animal Center were intraperitoneally injected with 25 mg/kg KYA1797K. After 12 h, the mice were challenged with a dose of HSV-1 or VSV via intravenous injection. Then, the mice were treated with 25 mg/kg KYA1797K daily. We analyzed the survival rates and collected the blood from the orbital sinus for ELISA, and obtained different tissues from each mouse for viral titer analysis at the indicated time points.

### HBV plasmid-transfected mouse model

Here, 5-week-old C57BL/6 male mice were hydrodynamically injected with 6 μg pAAV/HBV1.2 plasmids for 10 days and then intraperitoneally injected with 20 mg/kg KYA1797K every two days. The mice were monitored for body weight change. Serum was collected to quantify HBV copy number using the HBV DNA Quantitative Detection Kit (Daan Gene) following the manufacturer’s guidelines.

### siRNA transfection

A total of 1 × 10^5^ cells were transfected with specific siRNA duplexes, which were administered via RNAi MAX (Invitrogen) as the manufacturer’s guidelines. The siRNAs against *AXIN1*, *AXIN2* and *USP35* were synthesized from RIBOBIO and the sequence were listed in Supplementary Table 1. Non-targeting siRNA was used as control.

### *Axin1*-KO cell generation using CRISPR/Cas9 system

*Axin1*-KO cell lines were generated by CRISPR/cas9 system (lentiCRISPR v2, Addgene plasmid# 52961). The sequences of the single guide RNA (sgRNA) were presented in Supplementary Table 1. An empty vector was used as a control (LentisgVC). The lentiCRISPR virus were packaged using PSPAX2 plasmid (Addgene plasmid#12260) and PMD2.G (Addgene plasmid #12259). Subsequently, 2 × 10^6^ THP-1 cells and MEFs were infected with lentiviral media for 6 h in the presence of 4 mg/mL polybrene, followed by selection with 0.5 and 4 μg/mL puromycin for THP-1 and MEF cells, respectively. Knockout efficiency was verified by immunoblotting.

### RNA and HSV-1 DNA extraction and quantitative real-time PCR

Total RNAs were isolated from cells with TRIZOL regents (T924, Sigma-Aldrich) following the manufacturer’s instructions. cDNA was synthesized by reverse transcribing 1 μg RNA in a 20 µL reaction mixture with a kit (A5001, Promega). The mRNA expression level was analyzed by qPCR using the LightCycler 480 SYBR Green I Master (4887352001, Roche). The gene-specific primers were listed in Supplementary Table 1. The HSV-1 DNA was meticulously extracted from the cellular samples employing the Omega Tissue DNA Mini Kit (Omega).

### TBK1 purification

2.5 × 10^6^ 293 F cells were transfected with 1 mg of PCAGGS-TBK1-Flag for 36 h. The cells were then harvested by centrifugation at 8000 rpm for 1 h at 4 °C to collect the cell pellets. Subsequently, the cellular pellets were suspended in a lysis solution formulated with 25 mM Tris (pH 7.5), 300 mM sodium chloride, and 0.5% N-Dodecyl-β-D-maltoside (DDM). The cellular lysates were obtained by subjecting the cells to high-pressure homogenization, followed by centrifugation at 20,000 rpm for 30 min. The resulting supernatant lysates were subjected to purification using Flag beads (Genescripts). The elution of the TBK1 proteins was accomplished by employing the flag peptide from Beyotime, at a concentration of 150 μg/mL, in a buffer that comprised 25 mM Tris (pH 7.5) and 300 mM sodium chloride. The TBK1 proteins underwent further purification via a Superdex 200 Increase 10/300 column, provided by GE Healthcare.

### In vitro phosphorylation

For TBK1 auto-phosphorylation, 4 µg TBK1 were incubated in a buffer consisting of 20 mM HEPES (pH 7.5), 150 mM NaCl, 10 mM MgCl_2_, 2 mM DTT, 100 µM ATP for 1 h at 30 °C.

### AXIN1 purification

To purify MBP-His6-AXIN1, MBP-His6-AXIN1-GFP, and MBP-His6-AXIN1-MCH proteins, the *E.coli* cells were transformed with their respective plasmids and cultured in TB. Induction with Isopropyl β-D-Thiogalactoside (IPTG) was carried out to stimulate expression of these proteins. The harvested cells were subsequently re-suspended in a binding buffer, which is composed of 50 mM Tris-Cl (pH 7.9), 2 M NaCl, and 10 mM imidazole. The cells were then lysed using a high-pressure homogenizer and subsequently sedimented at 20,000 rpm for 30 min. The resulting supernatants were purified using Amylose Resin (NEB). Following thorough washing with the binding buffer, the proteins were eluted with an elution buffer consisting of 50 mM Tris-Cl (pH 7.9), 2 M NaCl, and 10 mM maltose. Further purification of the AXIN1 proteins was performed using a Superdex 200 Increase 10/300 column (GE Healthcare) in a buffer composed of 20 mM HEPES (pH 7.4) and 1 M NaCl.

### In vitro phase separation assay

AXIN1 proteins dissolved in a buffer containing 20 mM HEPES (pH 7.4) and 1 M NaCl were mixed with a buffer consisting of 20 mM HEPES (pH 7.4), adjusting the NaCl concentration to 150 mM. Subsequently, 5 μM AXIN1 proteins were treated with phosphorylated TBK1 for 1 h at 30 °C, followed by treatment with 2% PEG8000. The NaCl concentration was further adjusted to 150 mM, and droplet formation was then examined. For imaging, droplets were observed on a glass-bottom cell culture dish using a Zeiss LSM 880 microscope. Zeiss LSM software (Version 4.2 SP1) and ZEN software 2012 (Zeiss) were utilized for image analysis.

### Immunoprecipitation

The HEK293T cells were transfected with appropriate empty plasmids or constructed plasmids on 10 cm^2^ dishes with Lipo3000 for 36 h. Then, the cells were harvested with lysis buffer [50 mM HEPES, pH 7.4; 150 mM NaCl; 5 mM EDTA; 0.1% Nonidet P 40 (NP40)] supplemented with protease inhibitor cocktail (044693159001, Roche). Following centrifugation at 12,000 × g at a temperature of 4 °C for a 20-minute interval, the supernatants were gathered and subsequently subjected to an incubation with 20 µL of either Anti-c-myc Agarose Affinity Gel (product code A7470, Sigma) or Anti-Flag M2 Affinity Gel (product code A2220, Sigma), extending throughout the night at 4 °C. The beads were then washed five times with lysis buffer and eluted with 2× SDS sample buffer followed by boiling for 5 min at 100 °C. Immunoblot analysis was carried out for detection of indicated antibodies. For endogenous co-immunoprecipitation, 8 × 10^6^ THP-1 cells were resuspended in 1 mL lysis buffer according to the procedure indicated above.

### Immunostaining

The cells were seeded on coverslips transfected with 0.5 μg HA-RGS and Flag-IR for 24 h. After washing with PBS three times, the cells were fixed with 4% paraformaldehyde for 20 min at room temperature. After being permeabilized with 0.1% Triton X-100, the cells were blocked using bovine serum albumin (BSA) for 15 mins, followed by overnight incubation with primary antibodies against Flag and HA. Then, they were incubated with Alexa Flour 594-conjugated goat anti-rabbit and Alexa Flour 488-conjugated goat anti-mouse secondary antibodies. The confocal images were acquired and analyzed by Zeiss LSM 880.

### Immunoprecipitation for ubiquitination

The HEK293T cells were subjected to transfection with either Myc-tagged IRF3 and Flag-tagged AXIN1, or with a control vector, along with HA-tagged ubiquitination plasmids (HA-Ub, HA-K27-Ub, HA-K48-Ub, or HA-K63-Ub) in 10 cm^2^ dish for 36 h. The cells were then lysed using 200 µL lysis buffer (50 mM HEPES, 150 mM NaCl, 5 mM EDTA, 0.1% NP40) in the presence of protease inhibitor cocktail containing 1% SDS and boiled for 10 min. Then, the products were diluted by lysis buffer for 5 times to 1 mL and then subjected to an incubation at 4 °C for a duration of 30 min. After centrifugation for 20 min at 12,000 rpm, the supernatants were transferred to a new microcentrifuge tube for overnight incubation with myc beads. Then the products were subjected to immunoprecipitation and immunoblotting analysis.

### ELISA

The isolated HSV-1- or VSV-stimulated or poly(dA:dT)- or poly(I:C)-transfected BMDMs were seeded into 48 well plates (5 × 10^4^ cells) for 16 h. The culture medium was collected for IFN-α and IFN-β measurement according to the ELISA kit manufacturer guidelines (PBL).

### Lung histology

The lungs from virus-infected or uninfected mice were dissected, fixed in 10% formalin, and embedded into paraffin for hematoxylin and eosin (H&E) staining.

### Statistical analysis

Prism 8.0 (GraphPad Software; San Diego, CA, USA) was used for data analysis. Data are represented as the mean ± standard deviation (S.D.). Unless otherwise noted, statistical significance was determined using unpaired, two-tailed Student’s *t*-test for comparing two groups, one-way analysis of variance (ANOVA) with Dunnett’s multiple comparisons test for comparing more than two groups, and two-way ANOVA for comparing more than two groups with two or more time points. *P < 0.05; **P < 0.01; ***P < 0.001; ****P < 0.0001 and ns, not significant.

## Supplementary information

supplementary-clean

Western blot source data

Dataset-AI-proof-figures

## Data Availability

Publicly Available Datasets: The genetic variation data and RNA expression profiles in this study have been sourced from the Genomic Data Commons (GDC)- TCGA Liver Cancer (LIHC) dataset (https://portal.gdc.cancer.gov/). The mass spectrometry raw data that generated in this study have been submitted and are publicly accessible at ProteomeXchange (https://www.proteomexchange.org/), with a dataset identifier PXD055574.
