# Supplementary material for: AXIN1 boosts antiviral response through IRF3 stabilization and induced phase separation
Source: Signal Transduct Target Ther. 2024 Oct 9;9:281. doi: 10.1038/s41392-024-01978-y (PMC11464762; doi:10.1038/s41392-024-01978-y)
Supplement: Supplementary file 1 — supplementary-clean [file 41392_2024_1978_MOESM1_ESM.docx]

Supplementary Materials for

**AXIN1 boosts antiviral response through IRF3 stabilization and induced phase separation**

Authors: Dan-Ling Dai^1,*^, Chu Xie^1,*^, Lan-Yi Zhong^1,*^, Shang-Xin Liu^1^, Le-Le Zhang^1^, Hua Zhang^2^, Xing-Ping Wu^3^, Zhou-Ming Wu^1^, Kexin Kang^4^, Yan Li^5^, Ya-Meng Sun^1^, Tian-Liang Xia^1^, Chen-Song Zhang^6^, Ao Zhang^3^, Ming Shi^7^, Cong Sun^1^, Mei-Ling Chen^8^, Ge-Xin Zhao^1^, Guo-Long Bu^1^, Yuan-Tao Liu^1^, Kui-Yuan Huang^9^, Zheng Zhao^1^, Shu-Xin Li^1^, Xiao-Yong Zhang^9^, Yun-Fei Yuan^7^, Shi-Jun Wen^10^, Lingqiang Zhang^11^, Bin-Kui Li^7,✉^, Qian Zhong^1,✉^, Mu-Sheng Zeng^1,✉^

Correspondence to: [zengmsh@sysucc.org.cn,](mailto:zengmsh@sysucc.org.cn,) [zhongqian@sysucc.org.cn](mailto:zhongqian@sysucc.org.cn), [libk@sysucc.org.cn](mailto:libk@sysucc.org.cn).

**This PDF file includes:**

Materials and Methods

Supplementary Figures 1-11

Supplementary Table 1

Materials and Methods

**Reagents and antibodies.** ELISA kits for murine IFN-α and IFN-β (PBL); lipofectamine 3000 (Invitrogen); poly(dA:dT) (InivoGen); poly(I:C) (UBIO); SYBR (Roche); M-CSF (R&D); Bafilomycin A1 (Baf-A1) (Selleck), MG-132 (Selleck), KYA1797K (Selleck), and Cycloheximide (A8244-1000, APExBio). Anti-Flag (66008-1-Ig, 1:1000, Proteintech), anti-flag (F4049, 1:1000, Sigma), anti-myc (16286-1-Ig, 1:1000, Proteintech), anti-β-actin (66009-1-Ig, 1:4000, Proteintech), anti-HA (c29F4, Cell signaling technology), anti-GAPDH (60004-1-Ig, 1:4000, Proteintech), anti-AXIN1 (C76H11, 1:1000, Cell signaling technology), anti-AXIN1 (2B11, 1:500, Santa Cruz), anti-GFP (sc-9996, 1:1000, Santa Cruz), anti-GST (10000-O, 1:2000, Proteintech), anti-USP35 (ab86791, Abcam), anti-IRF3 (11904S, 1:1000, Cell signaling technology), anti-IRF3 (ab68481, 1:1000, Abcam), anti-p62 (D5E2, 1:1000, Cell signaling technology), anti-p62 (ab109012, 1:1000, Abcam), anti-His (10E2, 1:2000, Abmart), anti-TBK1(ab109735, 1:1000, Abcam), anti-phosphorylated-TBK1(5483S, 1:1000, Cell signaling technology), anti-phosphorylated-IRF3 (Ser396) (AF2436, 1:1000, Affinity), and anti-ATG5 (12994S, 1:1000, Cell signaling technology).

**Plasmids.** *Axin1*-KO plasmids were generated using LentiCRISPR v2. The following plasmids were constructed with an empty pcDNA3.1 vector: Flag-AXIN1, Flag-AXIN1-1–211, Flag-AXIN1-209–346, Flag-AXIN1-346–505, Flag-AXIN1-505–862, Myc-AXIN1, Myc-IRF3, HA-IRF3, HA-AXIN1, Flag-TBK1. PCAGGS-TBK1-Flag were also generated. IRF3-GFP were generated from pEGFP vector. Additionally, 6B-Myc-IRF3, USPs, HA-Ub, HA-Ub-K48, and HA-Ub-K63 were gifted by Professor Hongbing Shu (Wuhan University, Wuhan, China), while Flag-NDP52, Flag-Tollip, Flag-NBR1, Flag-OPTN, and Flag-p62 were gifted by Professor Xiaofeng Zhu (Sun Yat-sen University, Guangzhou, China). Myc-IRF3-DP and Myc-IRF3-IR were gifted by Professor Jun Cui (Sun Yat-sen University, Guangzhou, China). MBP-His6-AXIN1, MBP-His6-AXIN1-GFP, and MBP-His6-AXIN1-MCH were gifted by Professor Yeguang Chen (Tsinghua University, Beijing, China).

**Cell lines.** HEK-293T, HeLa, BEAS-2B, Vero and Hepa1-6 cells were maintained in DMEM (C11995500BT, GIBCO; California). THP-1 cells, MEFs (MEF p53−/−) were cultured in RPMI medium 1640 supplemented with 10% (v/v) FBS. All cells were cultured at 37 °C in a humidified atmosphere comprising 5% CO_2_. HEK-293T, HeLa, and BEAS-2B cells were purchased from ATCC, while Hepa1-6 and THP-1 cells were kindly provided by Professor Yunfei Yuan (Sun Yat-sen University, Guangzhou, China) and Professor Erwei Song (Sun Yat-sen University, Guangzhou, China), respectively.

**BMDM isolation.** BMDMs were isolated from 5-9-week-old mice femurs and tibias and cultured in RPMI medium 1640 (C11875500BT, GIBCO; California) supplemented with 10% (v/v) fetal bovine serum (FBS; 10099-141C, GIBCO; Australia) and 0.1% macrophage-colony stimulating factor (M-CSF) for 4–6 days. The medium was refreshed once during culturing.

**Virus titer determination.** HSV-1-GFP and VSV-GFP were propagated on Vero cells and the titer was measured by plaque assay. Vero cells were seeded into 6 well plates at a confluency of 100% in DMEM supplemented with 10% FBS. After diluting the stock virus from 10^−2^ to 10^−9^, the viruses were transferred to Vero cells and incubated the cells for 3 h at 37℃. After discarding the viral suspension, 1% methyl cellulose is added to each well, and the cells are further cultured in the CO2 incubator. Distinct plaques become apparent after 2-3 days. Then, the medium was removed and the cells were fixed with 4% paraformaldehyde for 10 min at room temperature followed by staining with 0.5% crystal violet for 10 min. Finally, the plaques of air-dried wells were counted for pfu/ml titer.

**Sample preparation for mass spectrometry and REACTOME analysis.** A total of 5×10^6^ vector control or *Axin1*-KO Hepa1-6 cell lines were cultured in T-25 cm^2^ flasks, harvested, washed three times with phosphate-buffered saline (PBS). The supernatants were discarded and the cell pellets were sent to the Institute of Biomedical Sciences (Phoenix Center) for label-free proteomic studies using mass spectrometry followed by REACTOME enrichment pathway analysis.

**Supplementary Figures**


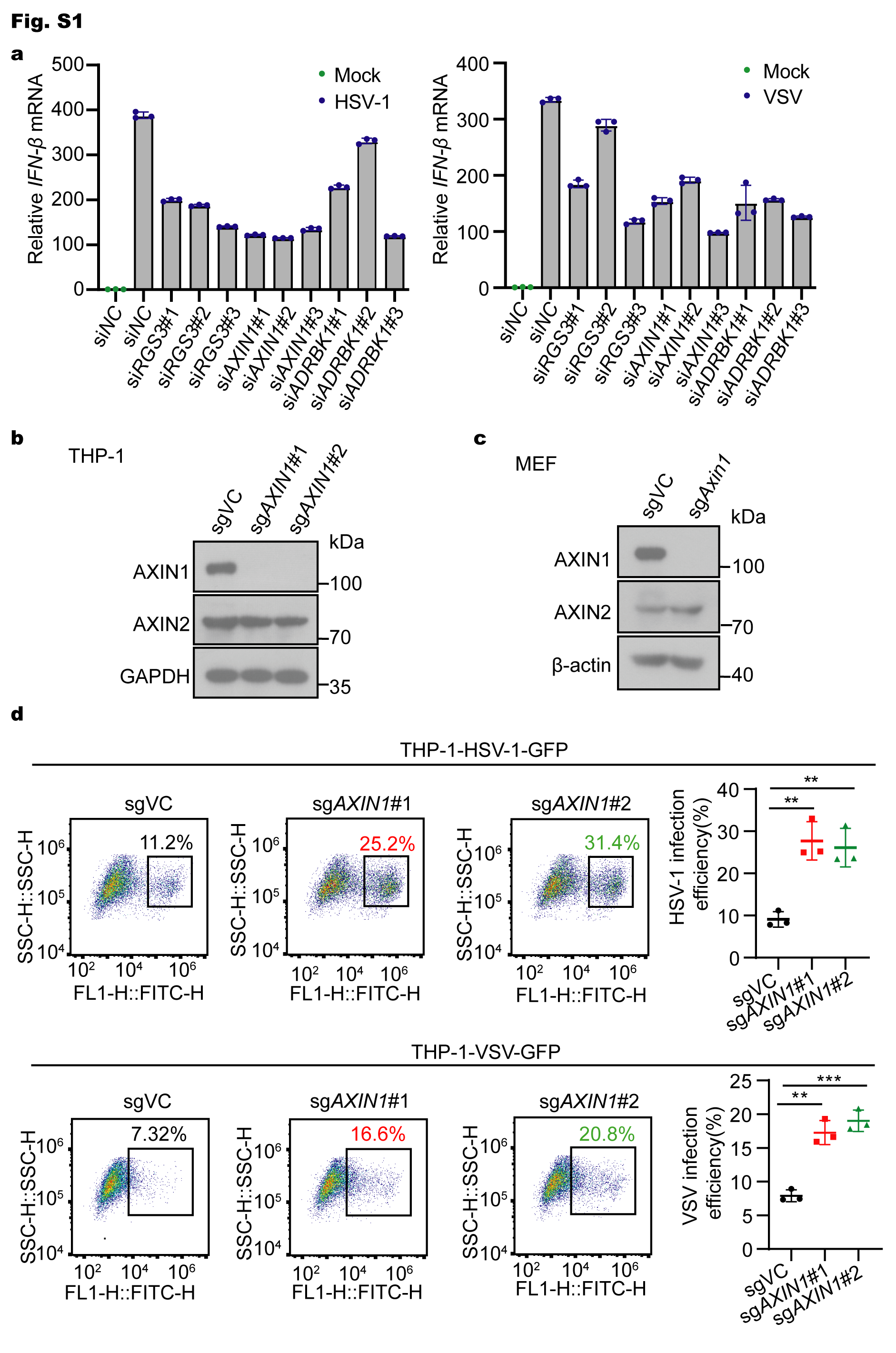


**Supplementary Fig. 1 | *AXIN1* KO impairs IFN-β expression and promotes DNA and RNA virus infections. a**, qPCR analysis of IFN-β mRNA in THP-1 cells transfected with three siRNAs targeting RGS3, AXIN1, and ADRBK1 individually followed by HSV-1 or VSV infection for 12 h (n=3). **b**, **c**, Immunoblotting assay of AXIN1 and AXIN2 in control vector (sgVC) or *AXIN1*-KO (sg*AXIN1*) (b) THP-1 and (c) MEFs. **d**, Flow cytometry analysis of HSV-1 or VSV amount in VC or *AXIN1*-KO THP-1 cells infected with HSV-1-GFP (MOI=0.05) or VSV-GFP (MOI=0.05) for 16 h (n=3). Data are shown as mean ± standard deviation (S.D.) and represent three independent experiments. Statistical analyses were performed using (**d**) one-way ANOVA with multiple comparisons test. *, *P* < 0.05; **, *P* < 0.01; ***, *P* < 0.001; ****, *P* < 0.0001. HSV-1, herpes simplex virus 1; MEF, mouse embryonic fibroblast; MOI, multiplicity of infection; RGS, regulator of G-protein signaling; siRNA, small interfering RNA.


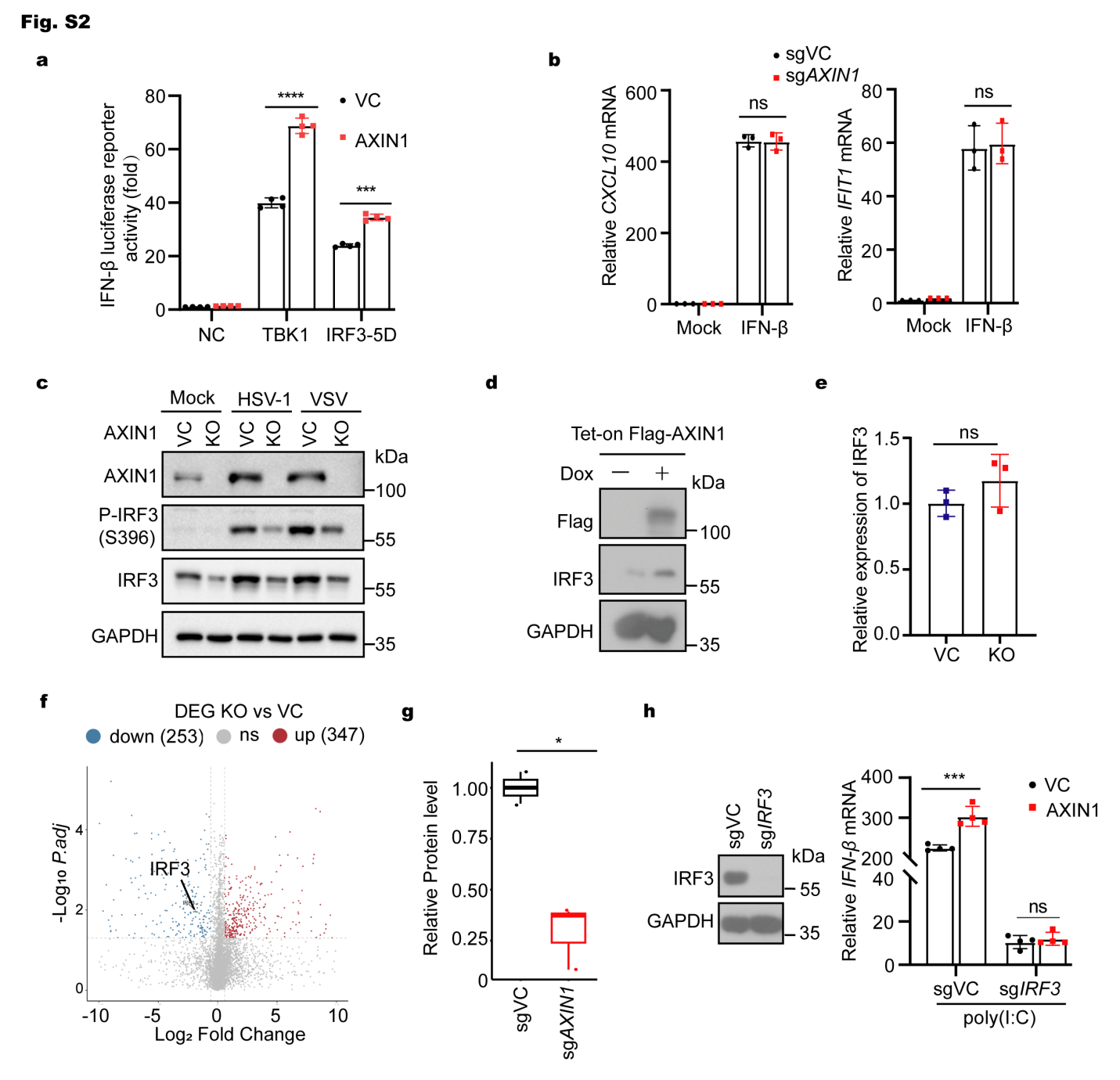


**Supplementary Fig. 2 |** ***AXIN1* KO decreases the total IRF3 protein at the resting-state. a**, Luciferase reporter assay of the *IFN-β* promoter activity in HEK293T cells co-transfected with a Flag-TBK1- or Flag-IRF3-5D expressing plasmid together with a Flag-AXIN1- or vector control (VC)-expressing plasmid (n=4). **b**, qPCR analysis of *CXCL10* and *IFIT1* mRNAs in sgVC or sg*AXIN1* THP-1 cells treated with 0.2 ng/mL IFN-β for 4 h (n=3). **c**, Immunoblotting assay of phosphorylated and total IRF3 protein in sgVC or sg*AXIN1* THP-1 cells infected with or without HSV-1 or VSV. **d**, Immunoblotting assay of IRF3 in AXIN1-inducible HeLa cells treated with or without 50 ng/mL Dox. **e**, qPCR analysis of the IRF3 mRNA in VC or *Axin1*-KO MEFs (n=3). **f,** Proteomic mass spectrometry analysis of differentially expressed proteins between vector control (VC) (n=2) or *AXIN1*-KO (n=3) THP-1 cells. Blue dots, significantly down-regulated proteins. Red dots, significantly up-regulated proteins. Gery dots, not-significantly expressed proteins. **g,** Relative protein level of IRF3 in vector control or *AXIN1*-KO THP-1 cells. **h**, Immunoblotting assay (left) indicating IRF3 protein expressed in VC or *IRF3*-KO BEAS-2B cells. qPCR analysis (right) of IFN-β mRNA in VC or *IRF3*-KO BEAS-2B cells transfected with VC or AXIN1-expressing plasmid followed by poly(I:C) treatment for 6 h (n=4). Data are shown as mean ± standard deviation (S.D.) and represent three independent experiments. Statistical analyses were performed using (**a, b, e, f, g, h**) Student’s two-tailed unpaired *t*-test. *, *P* < 0.05; **, *P* < 0.01; ***, *P* < 0.001; ****, *P* < 0.0001; ns, not significant. Dox, doxorubicin; HSV-1, herpes simplex virus 1; IFN, interferon; IRF3, interferon regulatory factor 3; KO, knockout; MEF, mouse embryonic fibroblast; siRNA, small interfering RNA; VC, vector control; VSV, vesicular stomatitis virus.


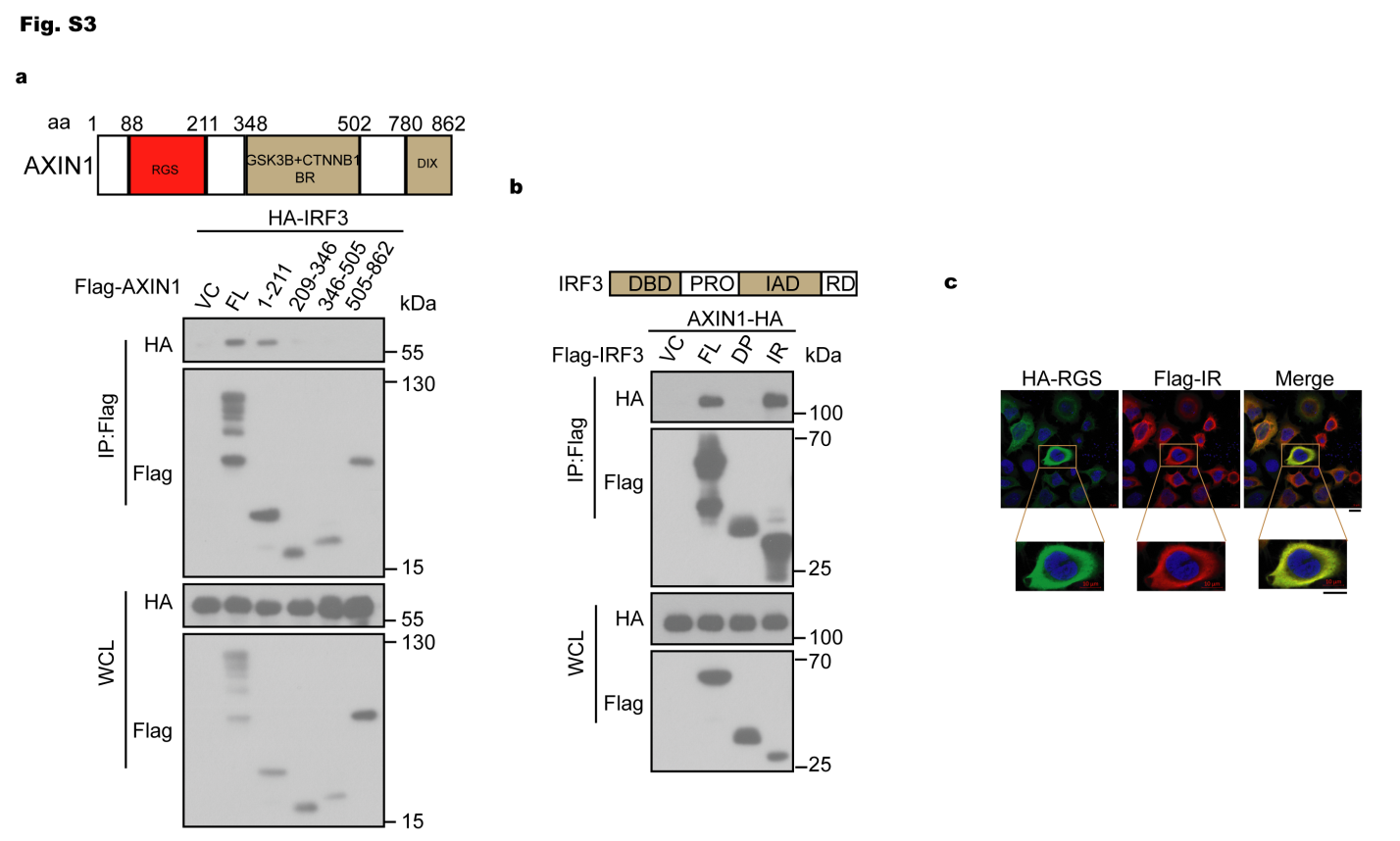


**Supplementary Fig. 3 | AXIN1 interacts with IRF3 depending on the RGS domain and IR domain. a**, HEK293T cell lysates transfected with a plasmid expressing HA-IRF3 together with a plasmid expressing Flag-tagged full-length or truncated AXIN1 or vector were immunoprecipitated with anti-Flag beads and immunoblotted with the indicated antibodies. **b**, HEK293T cell lysates transfected with a plasmid expressing HA-AXIN1 together with a plasmid expressing Flag-tagged full-length or truncated IRF3 or vector were immunoprecipitated with anti-Flag beads and immunoblotted with the indicated antibodies. **c**, HeLa cells were transfected with HA-RGS and Flag-IR followed by immunostaining with the indicated antibodies and confocal microscopy analysis. Scale bar represents 10 μm.


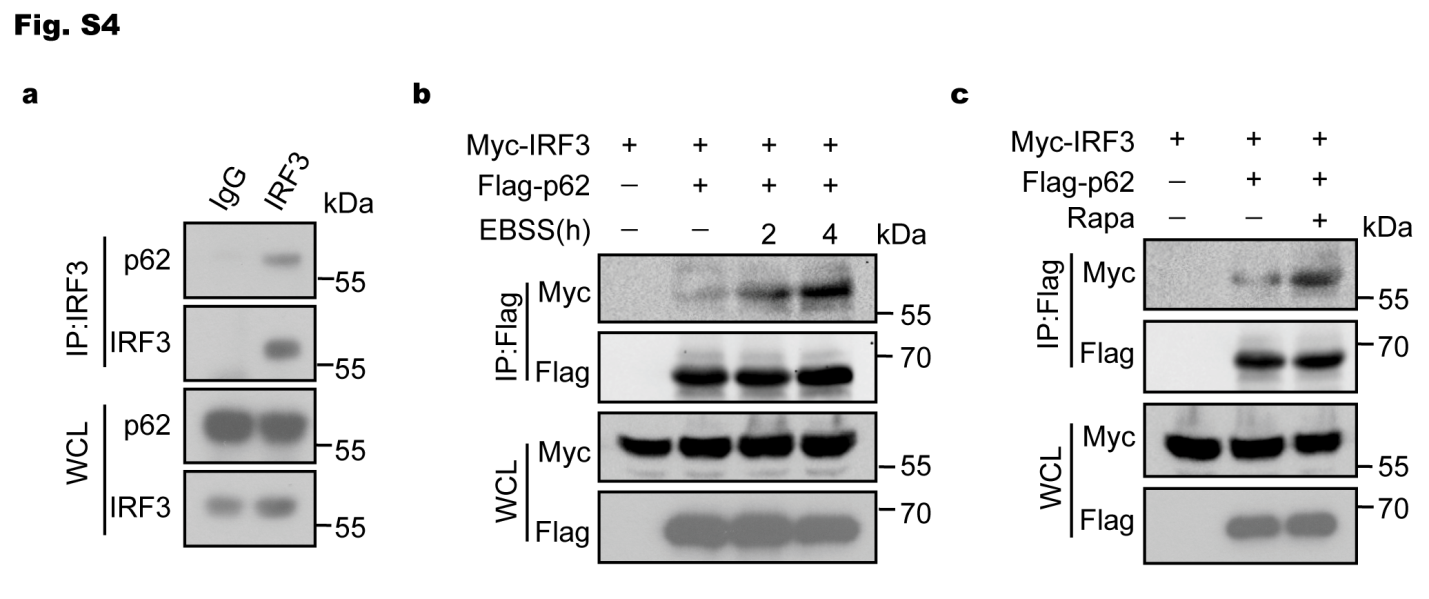


**Supplementary Fig. 4 | Autophagic IRF3 degradation is mediated by the cargo receptor p62.** **a**, THP-1 cell lysate was immunoprecipitated with anti-IRF3 beads and immunoblotted with the indicated antibodies. **b**, **c**, HEK293T cells transfected with Myc-IRF3 and Flag-p62 were treated with (**b**) EBSS for indicated time periods or (**c**) Rapa for 24 h followed by immunoprecipitation and immunoblotting with the indicated antibodies. Rapa, rapamycin; EBSS, Earle's Balanced Salt Solution.


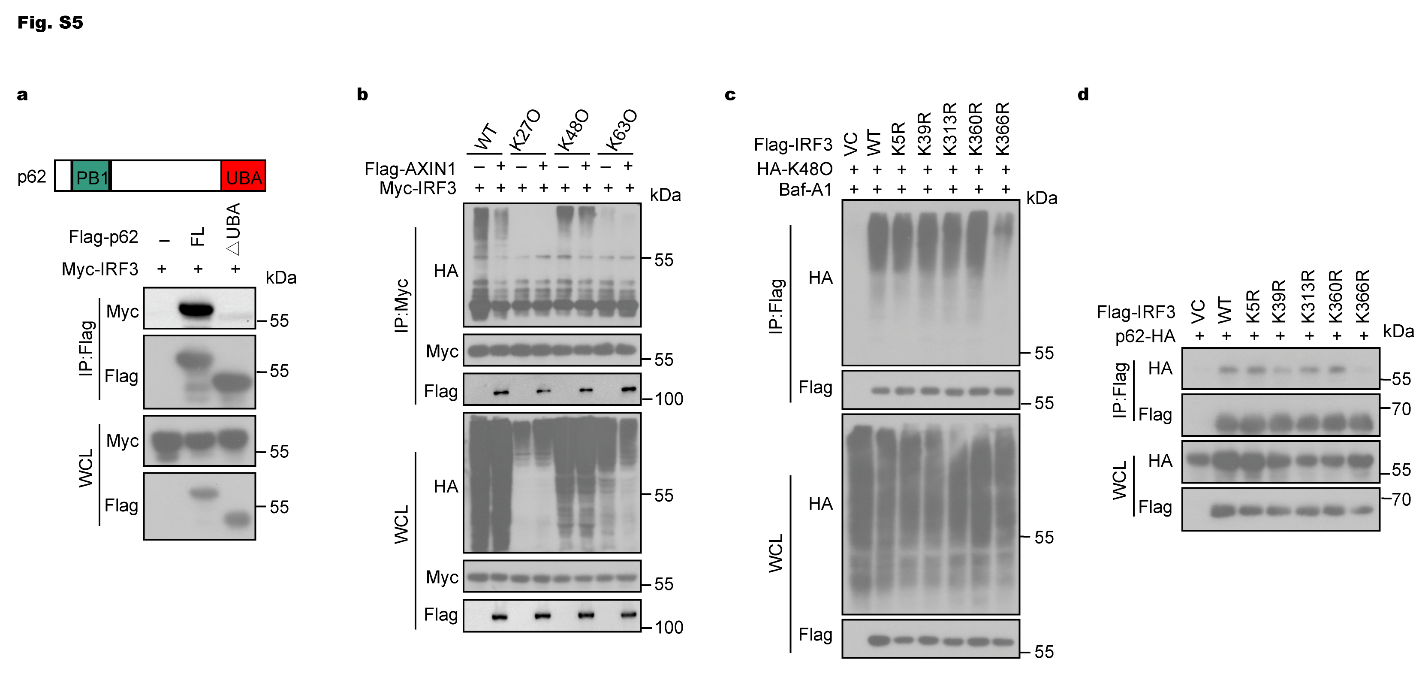


**Supplementary Fig. 5 | AXIN1 inhibits K48-linked IRF3 ubiquitination at K366.** **a**, HEK293T cells were transfected with plasmids expressing Myc-IRF3 and Flag-tagged full-length or a truncated p62 mutant or vector followed by immunoprecipitation and immunoblotting with the indicated antibodies. **b**, HEK293T cells were transfected with plasmids expressing Myc-IRF3, Flag-AXIN1 and HA-tagged WT or mutant (K27O, K48O, K63O) ubiquitin followed by Baf-A1 treatment for 4 h before immunoprecipitation with anti-Myc and immunoblotting with the indicated antibodies. **c**, HEK293T cells were transfected with HA-K48O ubiquitin and Flag-tagged WT or mutant IRF3 (K5R, K39R, K313R, K360R, K366R) followed by Baf-A1 treatment for 4 h before immunoprecipitating with anti-Flag beads and immunoblotting with the indicated antibodies. **d**, HEK293T cells were transfected with HA-p62 and Flag-tagged WT or mutant IRF3 as indicated followed by immunoprecipitation with anti-Flag beads and immunoblotting with the indicated antibodies. Baf-A1, bafilomycin A1; WCL, whole cell lysates; WT, wild-type.


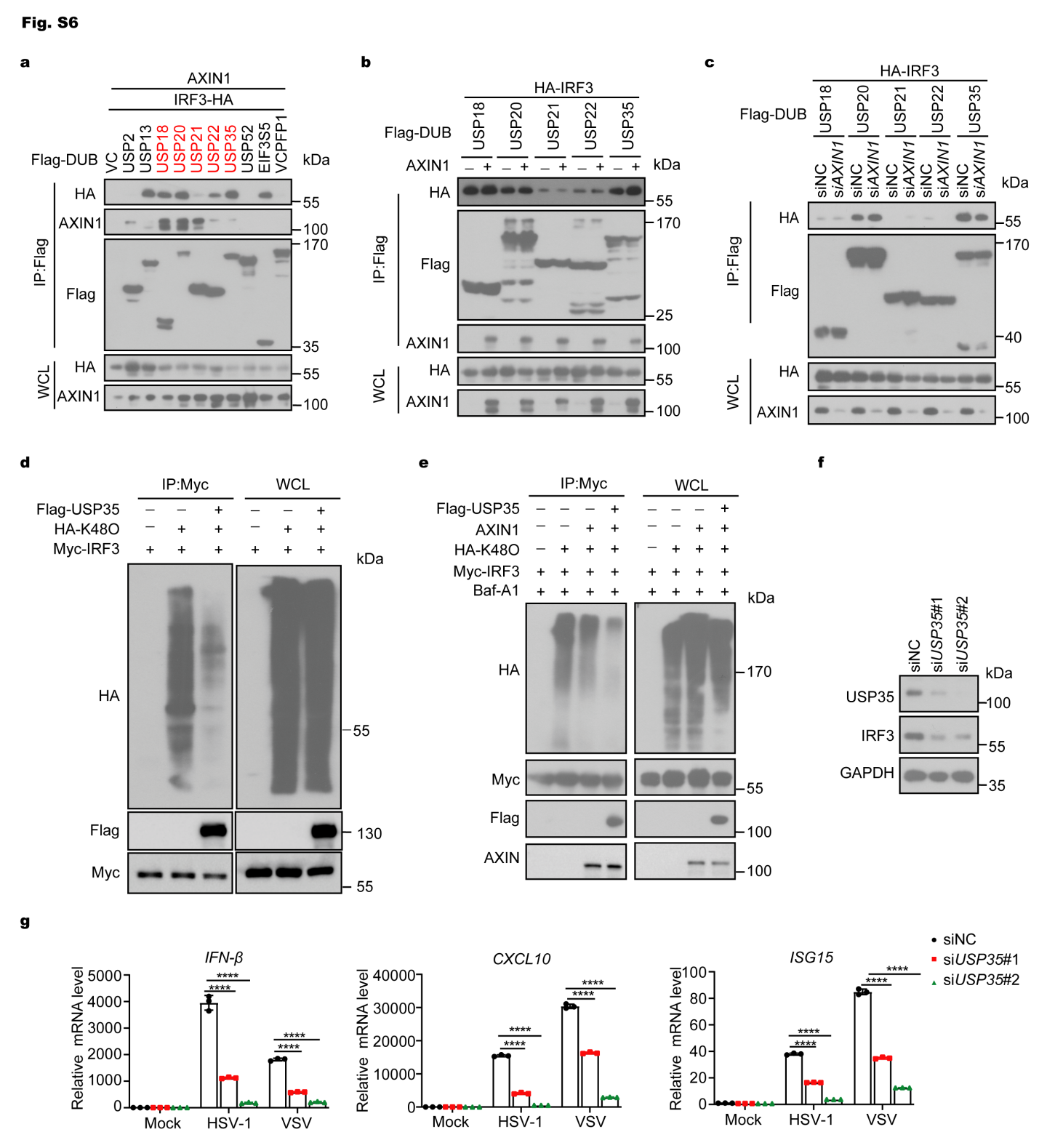


**Supplementary Fig. 6 | USP35 promotes IFN-I signaling by removing the K48-linked IRF3 ubiquitination.** **a**, HEK293T cells were transfected with plasmids expressing HA-IRF3, AXIN1, and several DUBs as indicated, followed by immunoprecipitation with anti-Flag beads and immunoblotting with the indicated antibodies. **b**, HEK293T cells were transfected with HA-IRF3, AXIN1, and Flag-DUBs (USP18, USP20, USP21, USP22, USP35) followed by immunoprecipitation with anti-Flag beads and immunoblotting with the indicated antibodies. **c**, HEK293T cells were transfected with AXIN1-RNAi (si*AXIN1*) for 24 h and then with a plasmid expressing DUBs and HA-IRF3, followed by immunoprecipitation with anti-Flag beads and immunoblotting with the indicated antibodies. **d**, HEK293T cells were transfected with plasmids expressing Flag-USP35, HA-K48O, and Myc-IRF3 as indicated, followed by immunoprecipitation with anti-Myc beads and immunoblotting with the indicated antibodies. **e**, HEK293T cells were transfected with plasmids expressing Myc-IRF3, HA-K48O, AXIN1, and Flag-USP35 and then treated with Baf-A1 for 4 h before immunoprecipitation with anti-Myc beads and immunoblotted with the indicated antibodies. **f**, Immunoblotting assay for IRF3 in BEAS-2B cells transfected with two independent USP35-specific (si*USP35*#1 and si*USP35*#2) or negative control (siNC) siRNAs. **g**, qPCR analysis of *IFN-β, CXCL10* and *ISG15* in BEAS-2B cells transfected with or without RNAi#*USP35* followed by HSV-1 or VSV infection for 12 h (n=3). Data are shown as mean ± standard deviation (S.D.) and represent three independent experiments. Statistical analyses were performed using (**g**) one-way ANOVA with Tukey’s multiple-comparison test. *, *P* < 0.05; **, *P* < 0.01; ***, *P* < 0.001; ****, *P* < 0.0001. siRNA, small interfering RNA; USP, ubiquitin specific peptidase; K, lysine.


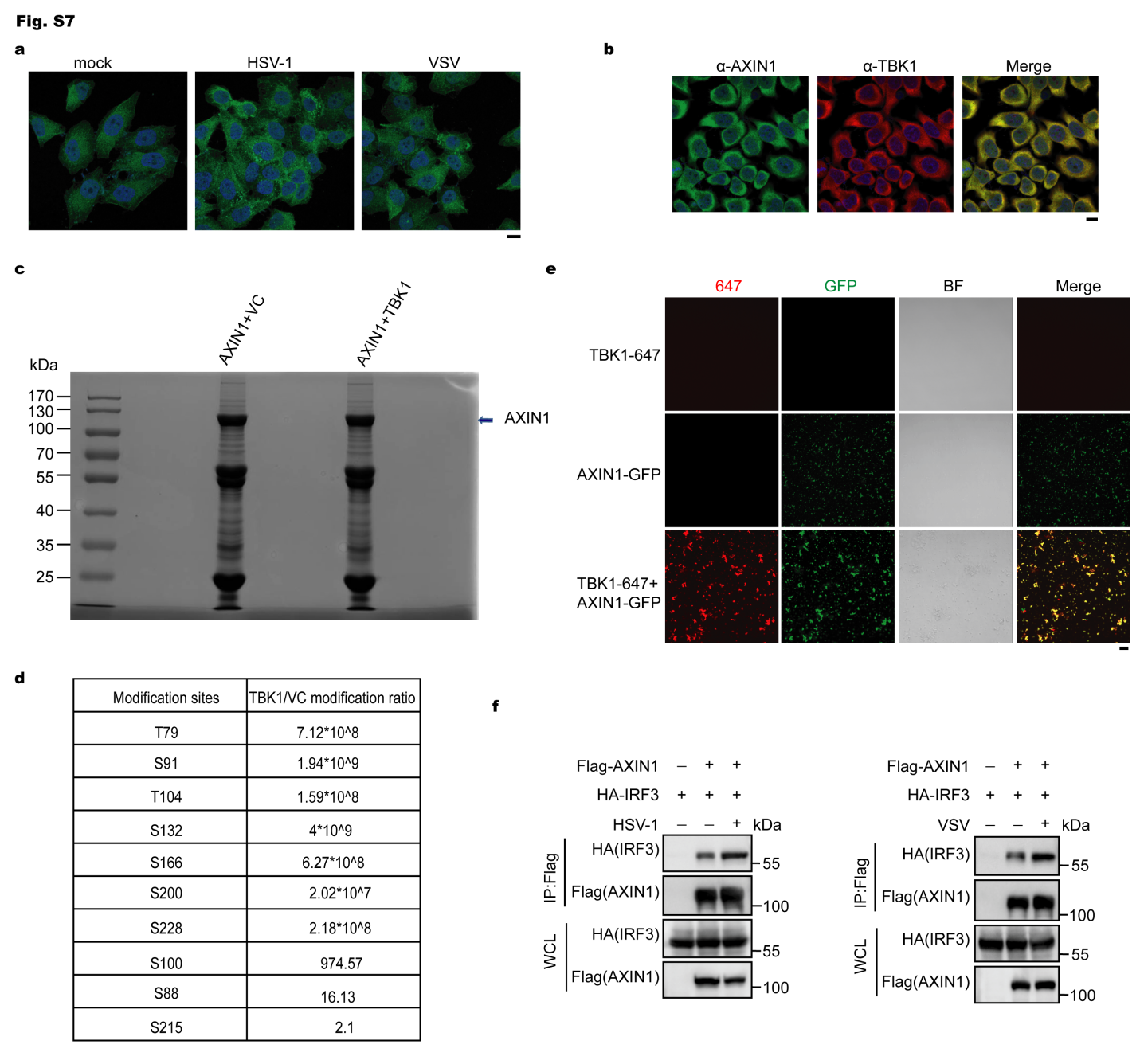


**Supplementary Fig. 7** | **AXIN1 could be phosphorylated by TBK1**. **a**, Immunostaining analysis of endogenous AXIN1 in Hela cells infected with HSV-1 and VSV for 3h. Scale bar represents 10 μm. **b**, Immunostaining analysis of endogenous AXIN1 and TBK1 in BEL-7407 cells. Scale bar represents 10 μm. **c**, HEK293T cells transfected with AXIN1-Flag and TBK1 or VC were immunoprecipitated with Flag-beads before harvesting for Coomassie Brilliant Blue staining. The arrow indicates the AXIN1 protein. **d**, The AXIN1 phosphorylation sites identified in AXIN1+TBK1 and AXIN1+VC group from (**c**) by mass spectrometry analysis. **e,** 647-labeled phosphorylated TBK1, AXIN1-GFP or AXIN1-GFP with 647-labled phosphorylated TBK1 was subjected to microscopy analysis. Scale bar represents 10 μm. **f,** HeLa cells were transfected with HA-IRF3 and Flag-AXIN1 or the related vector control followed by HSV-1 or VSV (MOI=1) infection for 3 hours. Immunoprecipitating were performed using Flag-beads and then analyzed with the indicated antibodies. AXIN1, Axis inhibition protein 1; IRF3, interferon regulatory factor 3; WCL, whole cell lysate.


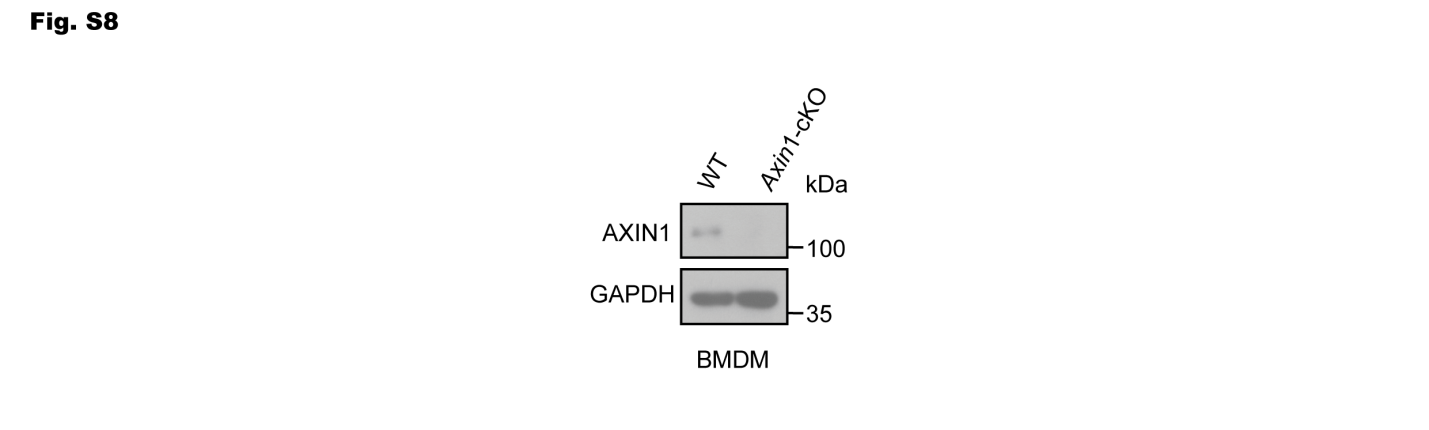


**Supplementary Fig. 8 | *Axin1* KO efficiency in BMDMs.** Immunoblotting assay of AXIN1 in BMDMs isolated from WT or *Axin1*-cKO mice. AXIN1, axis inhibition protein 1; BMDM, bone-marrow-derived macrophage; WT, wild-type; cKO, conditional knockout.


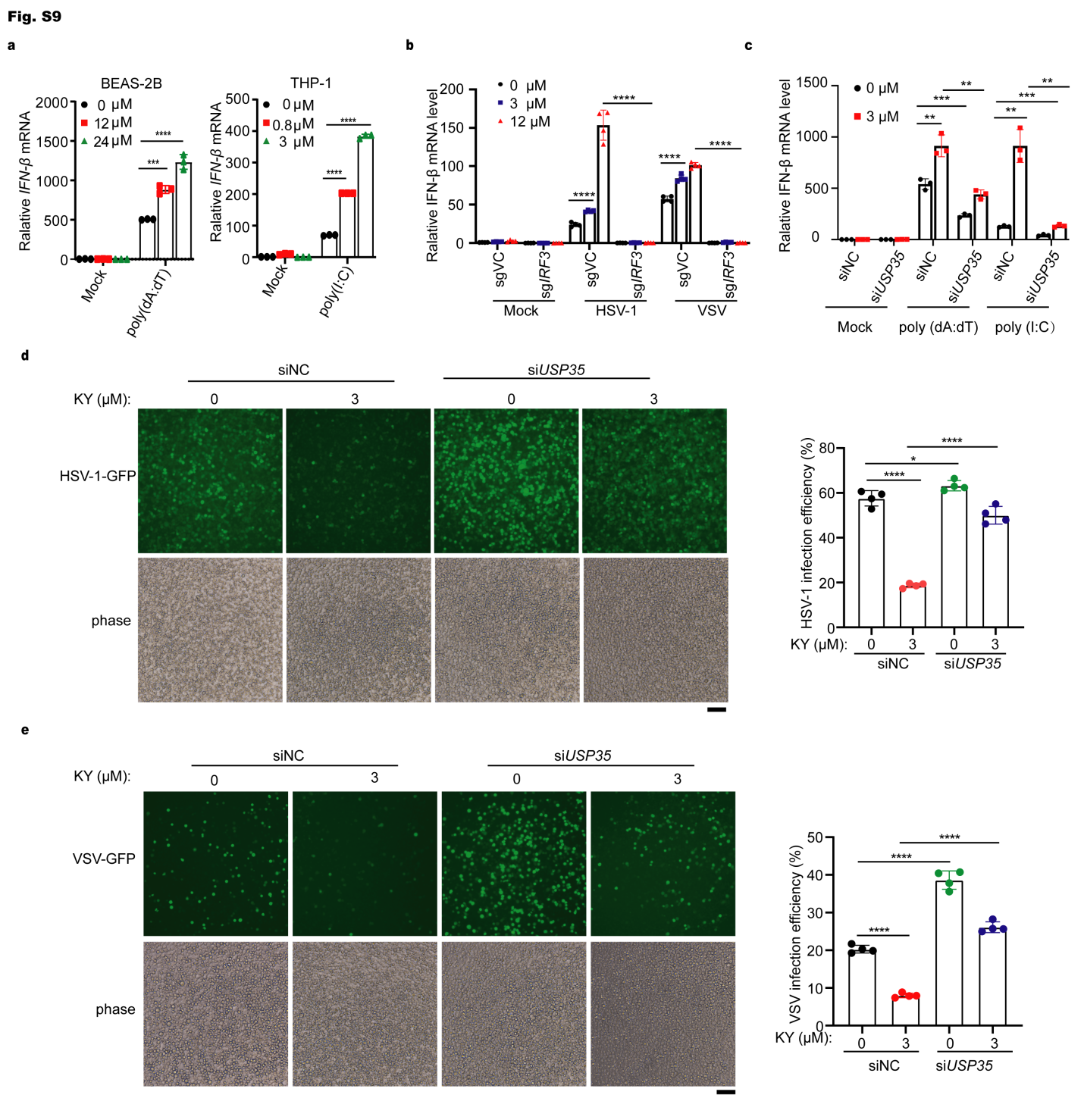


**Supplementary Fig. 9 | Knockdown of USP35 hinders the upregulation of IFN-β expression induced by KYA1797K treatment. a,** qPCR analysis of the IFN-β mRNA in BEAS-2B and THP-1 cells treated with different KYA1797K concentrations, followed by 2 µg/mL poly(dA:dT) or poly(I:C) induction for 6 h (n=3). **b,** qPCR analysis of IFN-β mRNAs in vector control (sgVC) or *IRF3*-KO (sg*IRF3*) HeLa cells that were treated with 0, 3, 12 µM KYA1797K for 6 hours and were then stimulated with or without HSV-1 or VSV infection (n=4). **c,** qPCR analysis of IFN-β mRNAs in vector control (siNC) or *USP35*-KD (si*USP35*) THP-1 cells that were treated with or without 3 µM KYA1797K for 6 hours and were then stimulated with or without 2 μg/mL poly(dA:dT) or poly(I:C) for 6h (n=3). **d, e,** Microscopic imaging and flow cytometry analysis of siNC or si*USP35* THP-1 cells that were treated with or without 3 µM KYA1797K for 6 hours and then infected with (d) HSV-1-GFP (MOI=0.1) or (e) VSV-GFP (MOI=0.05) for 16 h (n=4). Scale bar in (**d, e**) represents 100 μm. Data are shown as mean ± standard deviation (S.D.) and represent three independent experiments. Statistical analyses were performed using (**a-e**) one-way ANOVA with multiple-comparison test. *, *P* < 0.05; **, *P* < 0.01; ***, *P* < 0.001; ****, *P* < 0.0001. KY refers to KYA1797K.

**
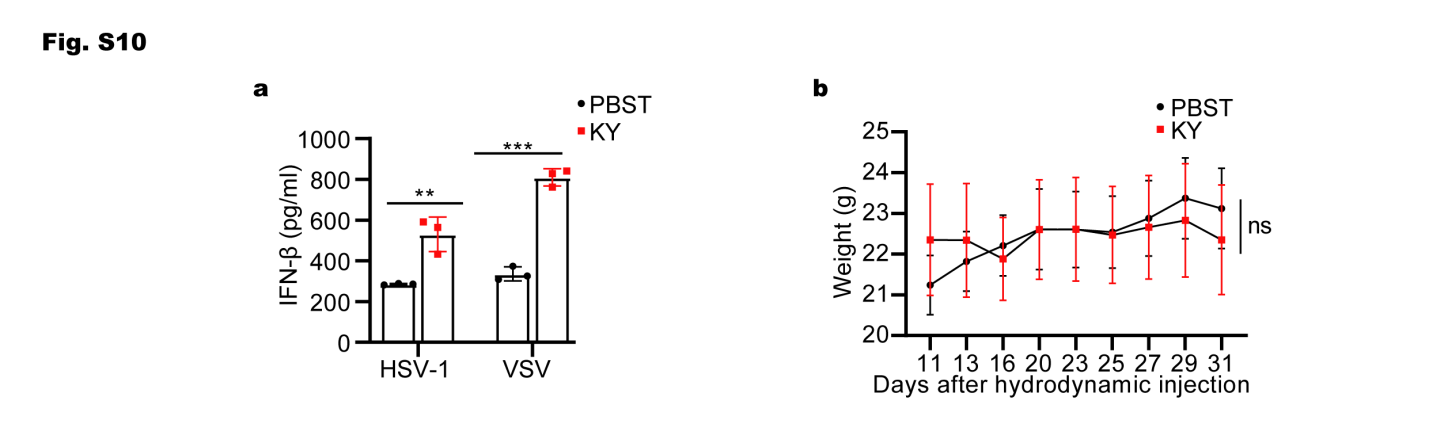
**

**Supplementary Fig. 10 | KYA1797K administration increases IFN-β secretion and does not obviously affect mice body weight.** **a**, ELISA analysis of IFN-β in the supernatant of BMDMs isolated from C57BL/6 mice treated with KYA1797K followed by HSV-1 and VSV infection for 16 h (n=3). **b**, Body weight analysis of C57BL/6 mice hydrodynamically injected with pAAV/HBV1.2 plasmid for 10 days and intraperitoneally injected with KYA1797K (n=10). Data are shown as mean ± standard deviation (S.D.) and represent two independent experiments. Statistical analyses were performed using (**a**) Student’s two-tailed unpaired *t*-test or (**b**) two-way ANOVA with Sidak’s multiple-comparison test. *, *P* < 0.05; **, *P* < 0.01; ***, *P* < 0.001; ****, *P* < 0.0001; ns, not significant. KY refers to KYA1797K.


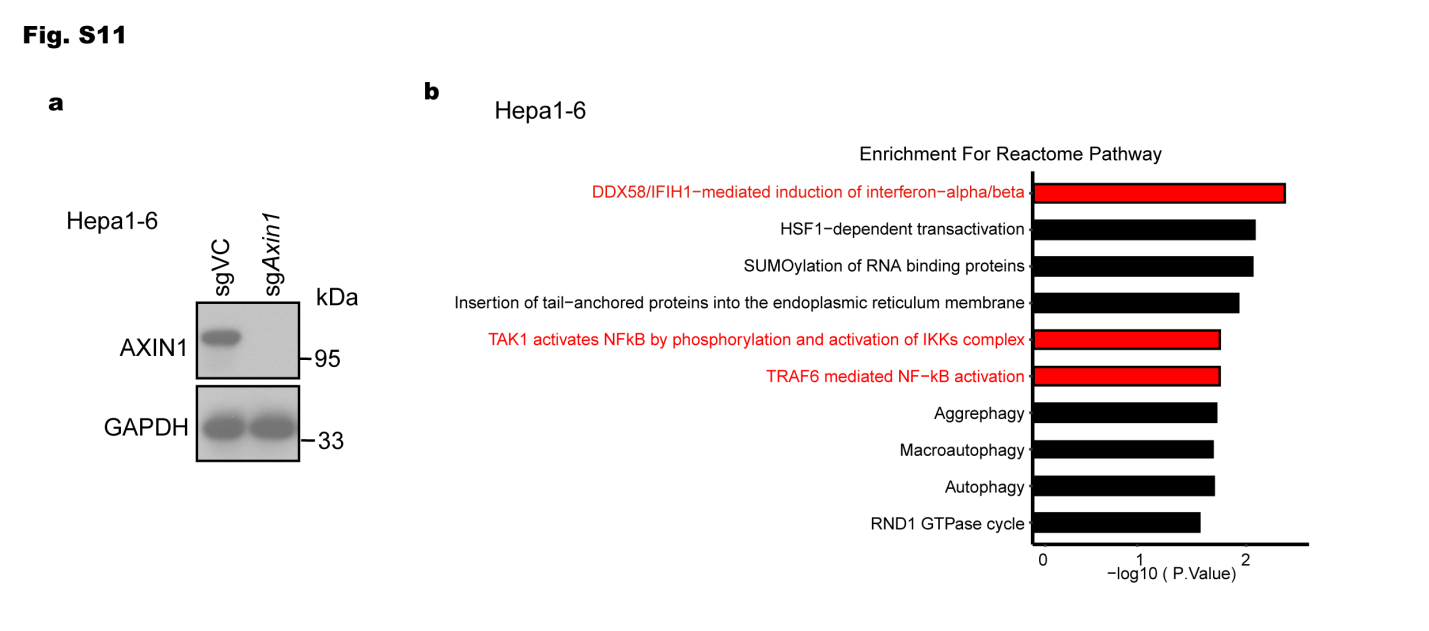


**Supplementary Fig. 11 | Differentially expressed proteins between *Axin1*-KO and control cells are mainly enriched in immune regulation pathway.** **a**, Immunoblotting assay of AXIN1 in sgVC or sg*Axin1* Hepa1-6 cells. **b**, Proteomic mass spectrometry of sgVC (n=4) and sg*Axin1* (n=4) Hepa1-6 cells. Differentially expressed proteins were analyzed by R Limma package. Pathway enrichment analysis was performed using differential expressed proteins (p<0.01) using the REACTOME website tools (<https://reactome.org/>). The top 10 enriched pathways are shown. The p-value indicates the significance of the enrichment. AXIN1, axis inhibition protein 1; VC, vector control.

Supplementary Tables

Supplementary Table 1. Sequences of primers and sgRNA

| **Name** | **Sense strand/sense primer (5′-3′)** | **Antisense strand/antisense primer (5′-3′)** | |
| --- | --- | --- | --- |
| **Primers for genotyping** | | | |
| Axin1-flox | CCAGCTGAAATTGCTGCTGCA | | TCCTTCTTCTATGCACGAAGACA |
| Lyz2-Cre | CCCAGAAATGCCAGATTACG | | CTTGGGCTGCCAGAATTTCTC |
| **sgRNA sequences** | | | |
| sgAXIN1#1 | CTGCTCGCTGTCGTTGGCAC | | GTGCCAACGACAGCGAGCAG |
| sgAXIN1#2 | TGCTGCTTACGGATCCTGTA | | TACAGGATCCGTAAGCAGCA |
| sgmAxin1 | TCAAGTAGACGGTACAACGA | | TCGTTGTACCGTCTACTTGA |
| **siRNA sequences** | | | |
| siAXIN1#1 | GCATCGTTGTGGCGTACTA | |  |
| siAXIN1#2 | GAAAGGTGTTGGCATTAAA | |  |
| siAXIN1#3 | GAAGCACGTACCCAAGTCA | |  |
| siAXIN2#1 | GTGAGTTGGTTGTCACTTA | |  |
| siAXIN2#2 | GAGCGATCCTGTTAATCCT | |  |
| siUSP35#1 | AGGACTGCTCGGAGTATCT | |  |
| siUSP35#2 | CAGCAGAAAACCGCTACTA | |  |
| **Primers for qPCR** | | | |
| IFN-β | TGTTGAGAACCTCCTGGCTAA | | TCCCCTGGTGAAATCTTCTTT |
| ACTB | GTGAAGGTGACAGCAGTCGGT | | AAGTGGGGTGGCTTTTAGGAT |
| IFN-α4 | GAGGGCCTTGATACTCCTGGCACA | | TCTAGGAGGCTCTGTTCCCAAGCA |
| ISG15 | TCCTGGTGAGGAATAACAAGGG | | GTCAGCCAGAACAGGTCGTC |
| CXCL11 | GGCAGATATTGAGAAAGCCTCC | | GCCTTGCTTGCTTCGATTTG |
| IFIT1 | TCAGGTCAAGGATAGTCTGGAG | | AGGTTGTGTATTCCCACACTGTA |
| IFIT2 | GGAGGGAGAAAACTCCTTGGA | | GGCCAGTAGGTTGCACATTGT |
| IFIT3 | TCAATAAGGAAGTCCCTGATGC | | TATGGACAAACCCTCTAAACCA |
| CXCL10 | TGGCATTCAAGGAGTACCTC | | TTGTAGCAATGATCTCAACACG |
| mCxcl10 | CCTGCCCACGTGTTGAGAT | | TGATGGTCTTAGATTCCGGATTC |
| mMx1 | GTGGTAGTCCCCAGCAATGT | | TGCTGACCTCTGCACTTGAC |
| mIfn-β | ATAAGCAGCTCCAGCTCCAA | | CTGTCTGCTGGTGGAGTTCA |
| mIfn-α4 | CCTGTGTGATGCAGGAACC | | TCACCTCCCAGGCACAGA |
| mActb | TGGTTACAGGAAGTCCCTCAC | | ACAGAAGCAATGCTGTCACCTT |
| mIfit1 | CAAGGCAGGTTTCTGAGGAG | | GACCTGGTCACCATCAGCAT |
| HSV | AACGCGTCCTTGTTCTCGGC | | TGAGGCGCGATTCTGGATGC |
| VSV | ACGGCGTACTTCCAGATGG | | CTCGGTTCAAGATCCAGGT |
| mIrf3 | GAGCGCCGAACGAGGTTCAG | | CTTCCAGGTTGACACGTCCG |
| USP35 | GGAGGAGCCACCATCTAGC | | ACACCCAGTCAATCATCCTGC |
