# Supplementary material for: AXIN1 boosts antiviral response through IRF3 stabilization and induced phase separation
Source: Signal Transduct Target Ther. 2024 Oct 9;9:281. doi: 10.1038/s41392-024-01978-y (PMC11464762; doi:10.1038/s41392-024-01978-y)

Source Fig.2a (left)

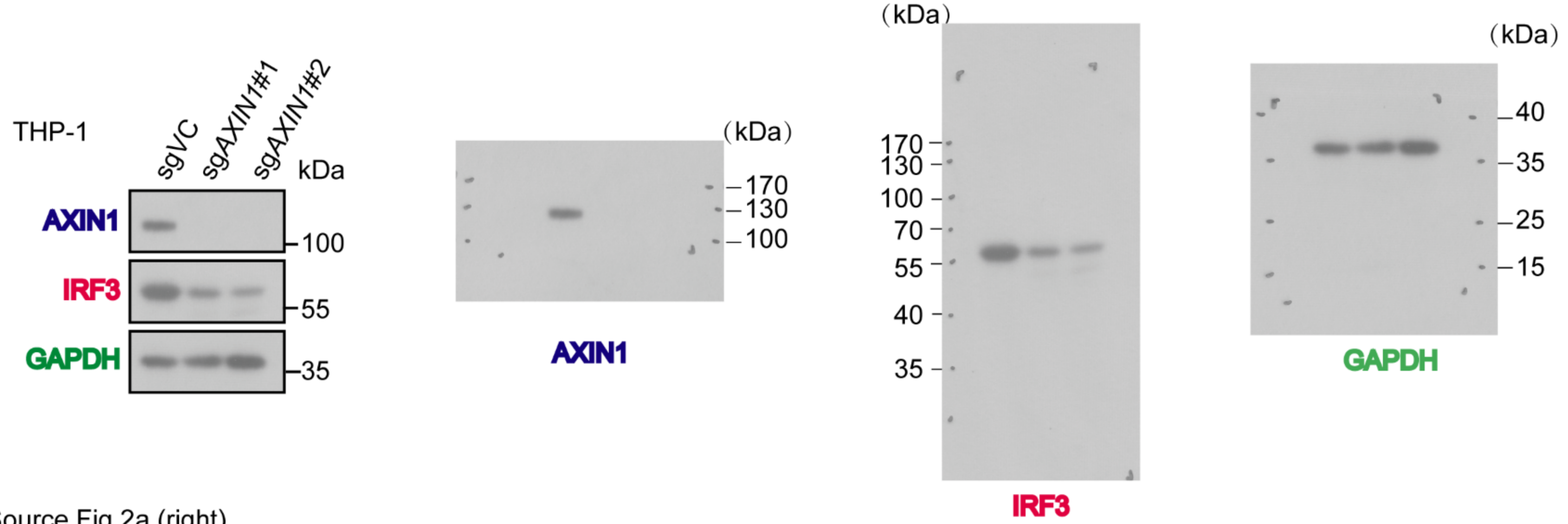

Source Fig.2a (right)

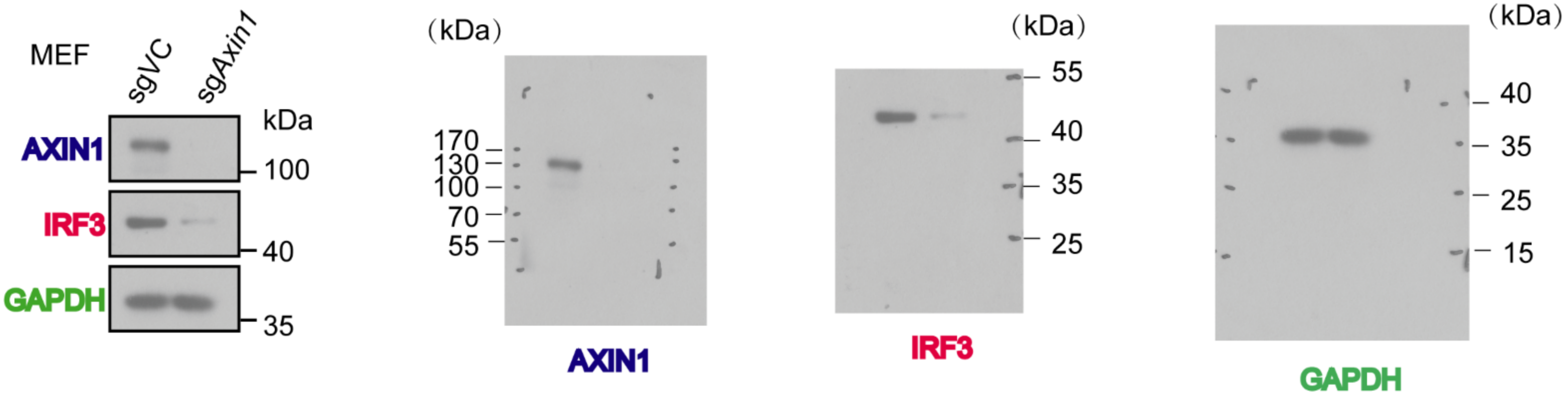

Source Fig.2b

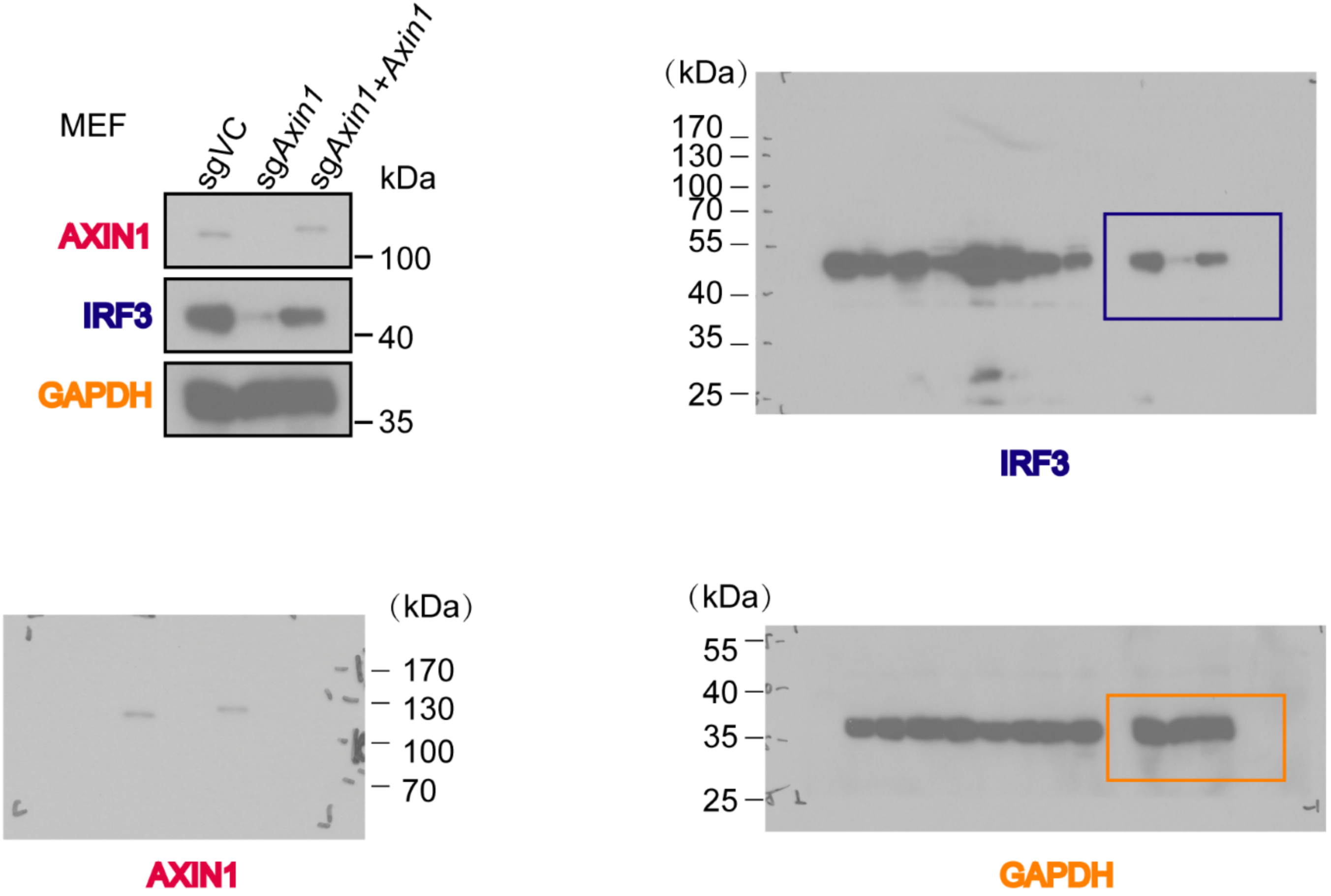

Source Fig.2c

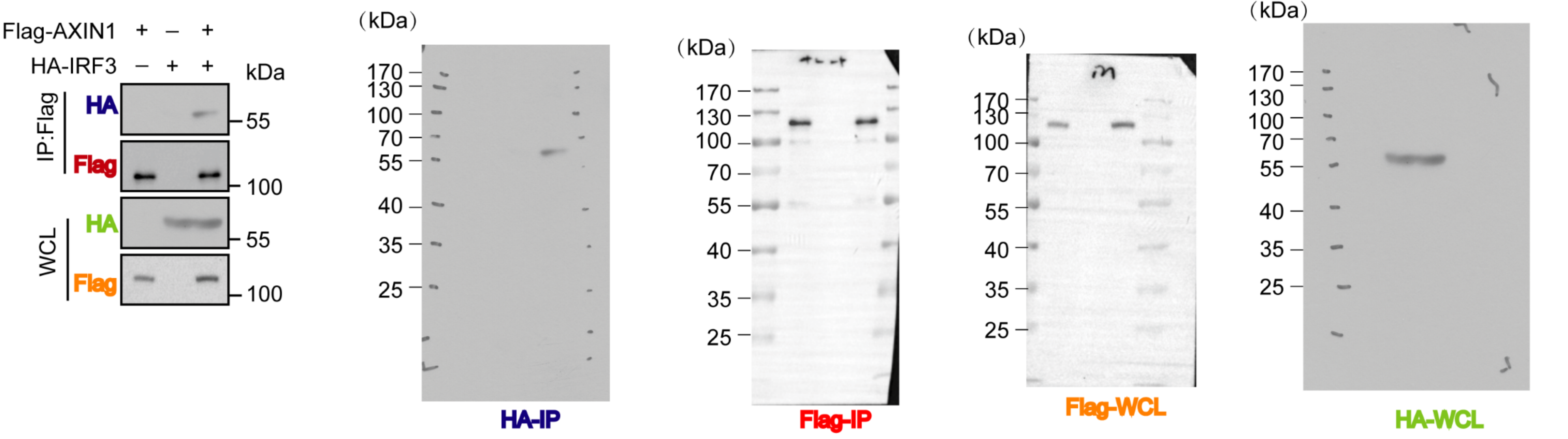

Source Fig.2e

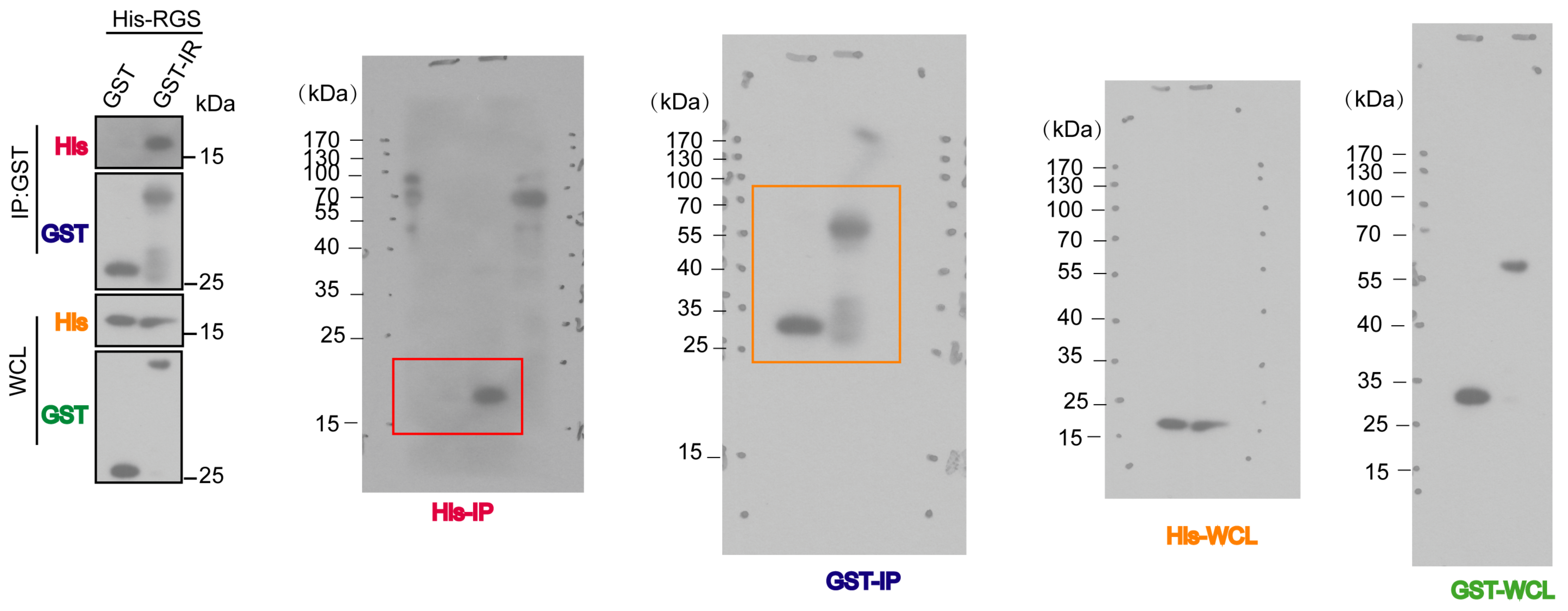

Source Fig.2g

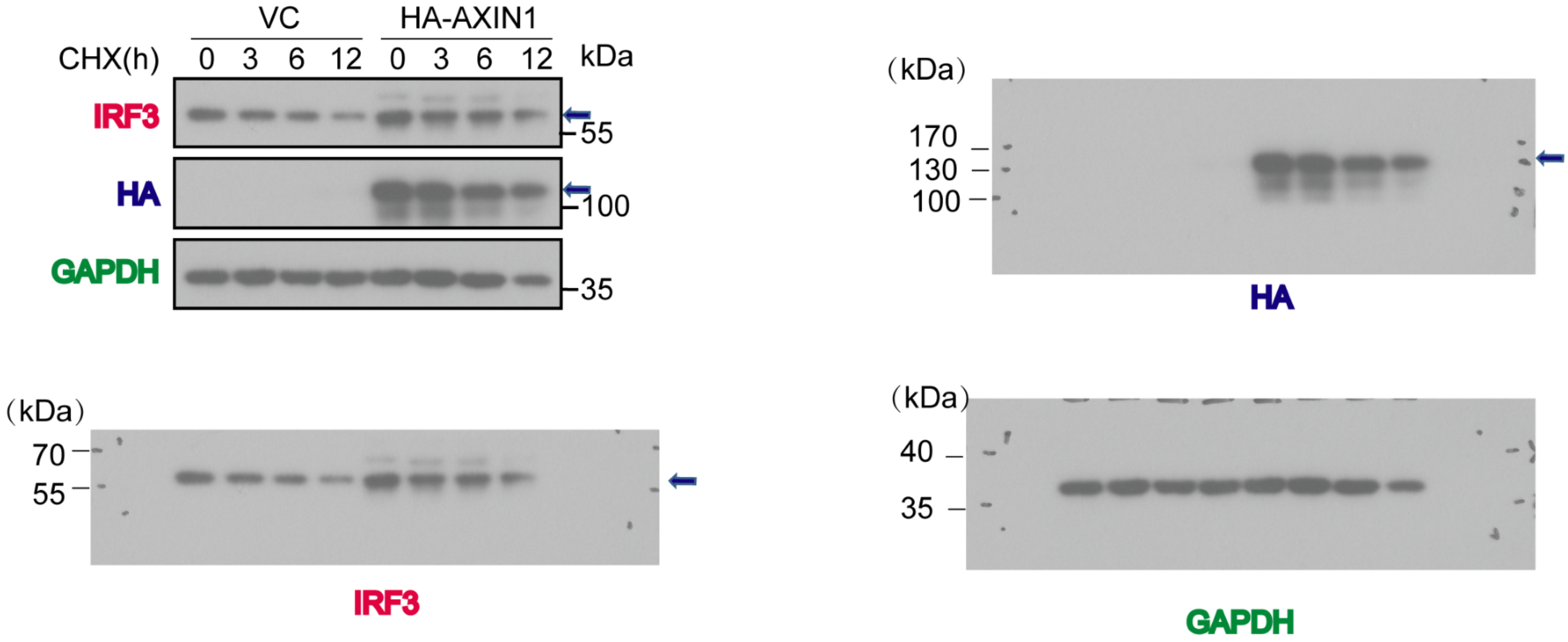

Source fig. 2h

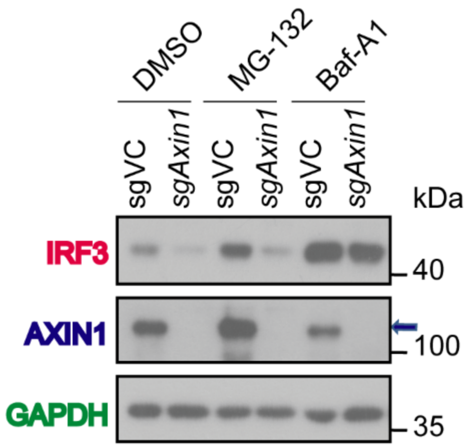

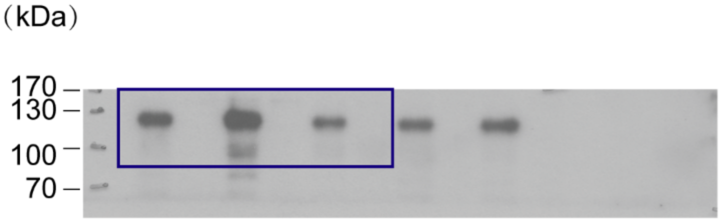

AXIN1

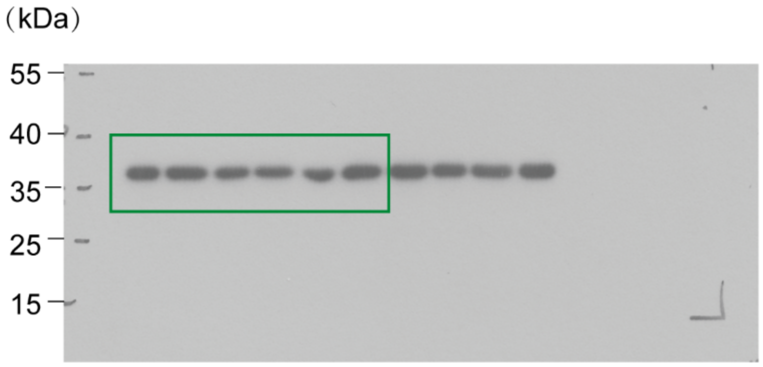

GAPDH

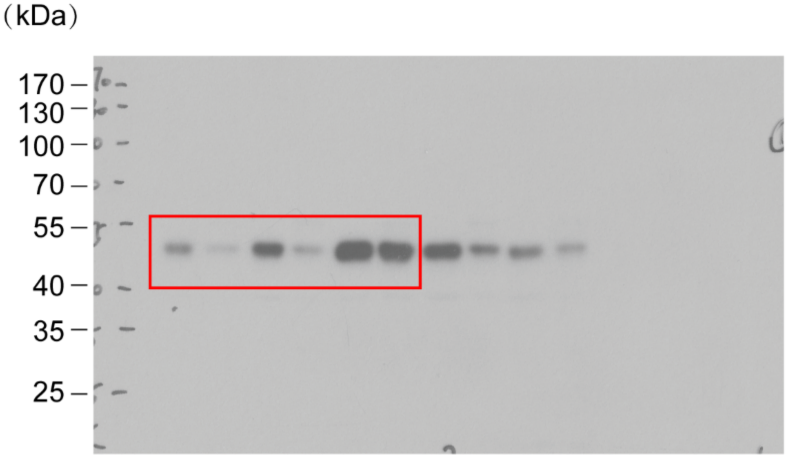

IRF3

Source Fig.2i

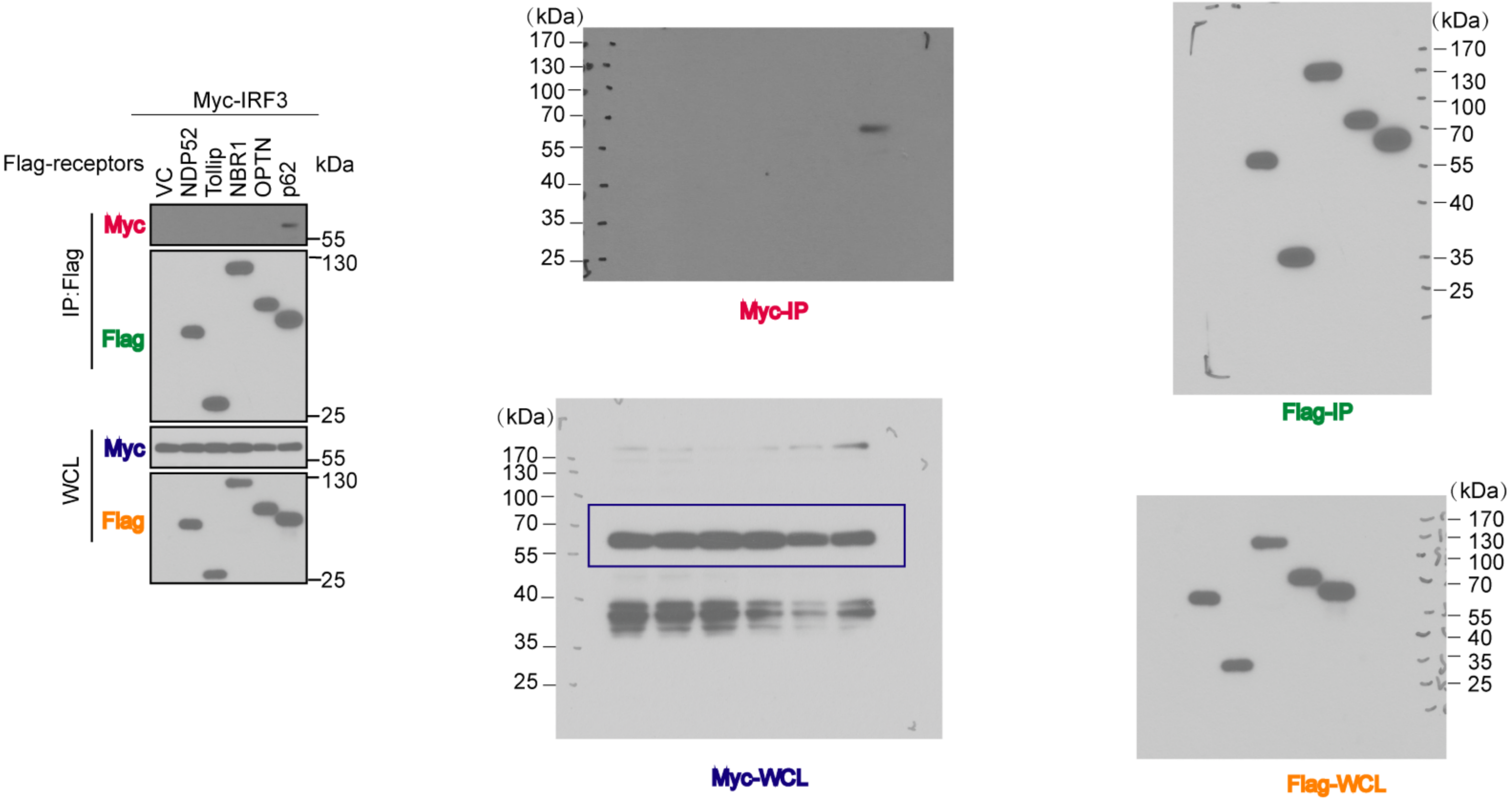

Source Fig.2j

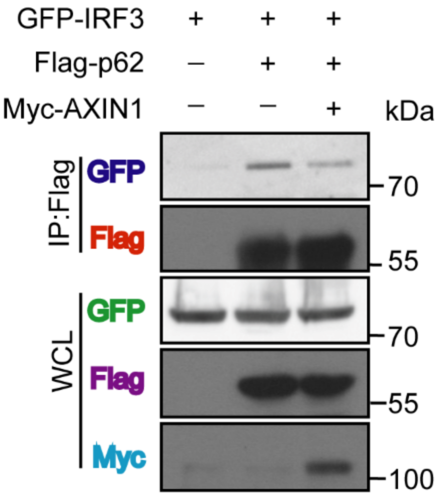

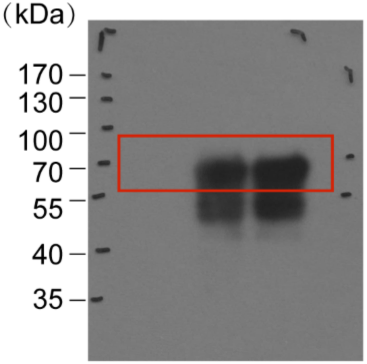

Flag-IP

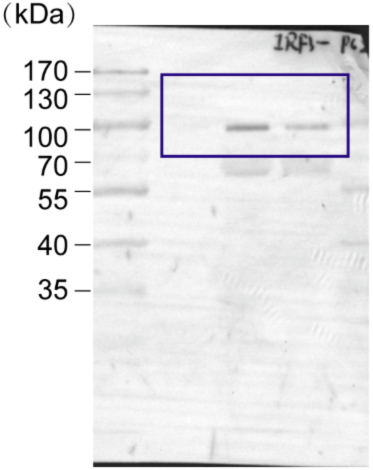

GFP-IP

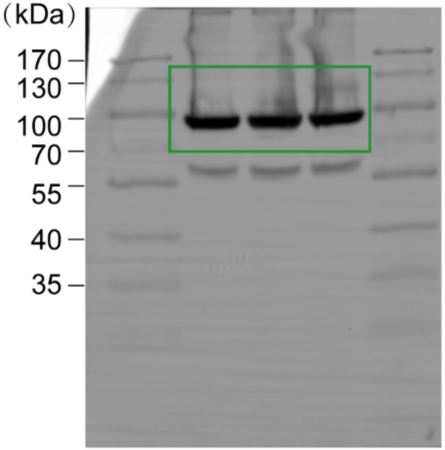

GFP-WCL

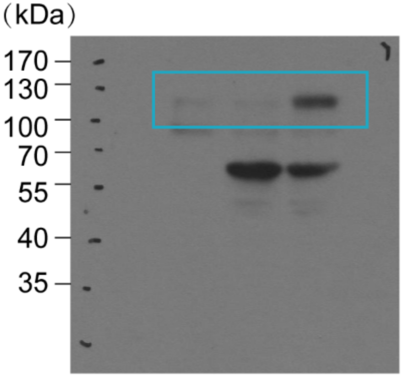

Myc-WCL

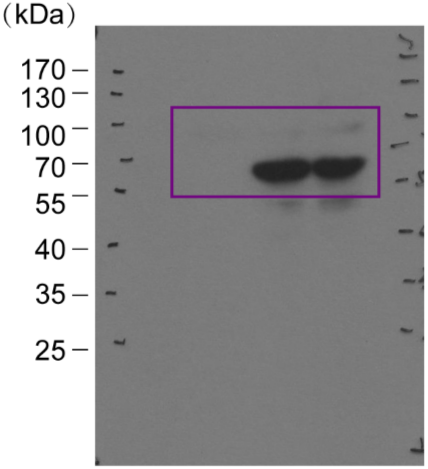

Flag-WCL

Source Fig.2k

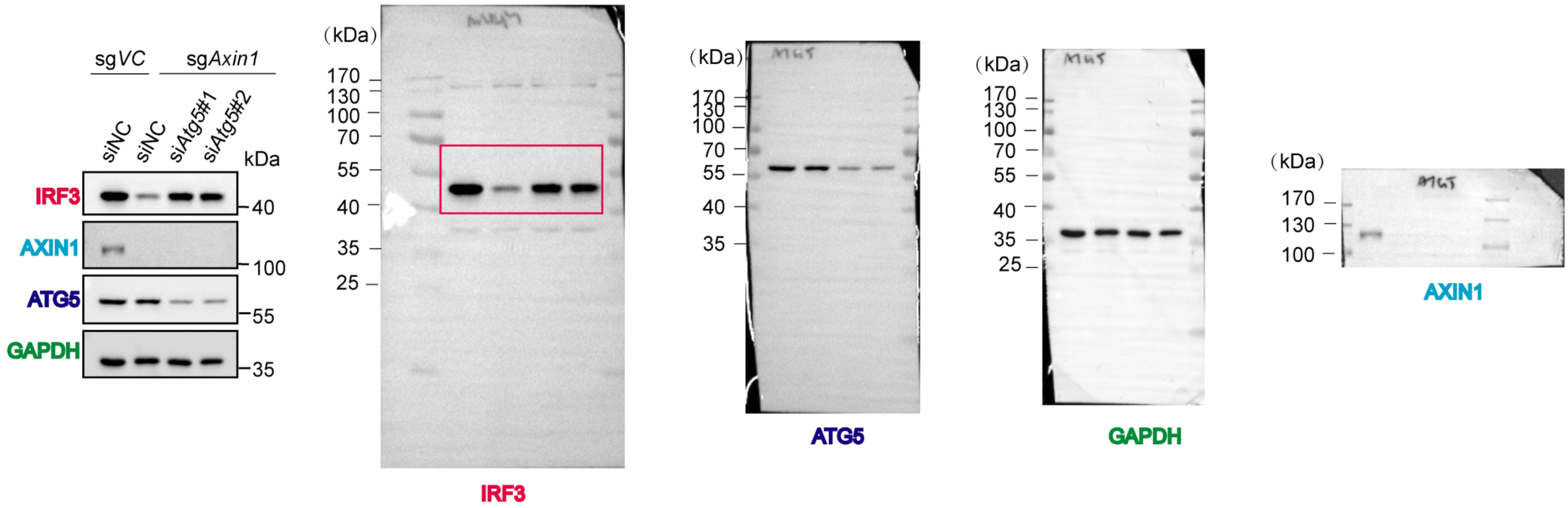

Source Fig.3c

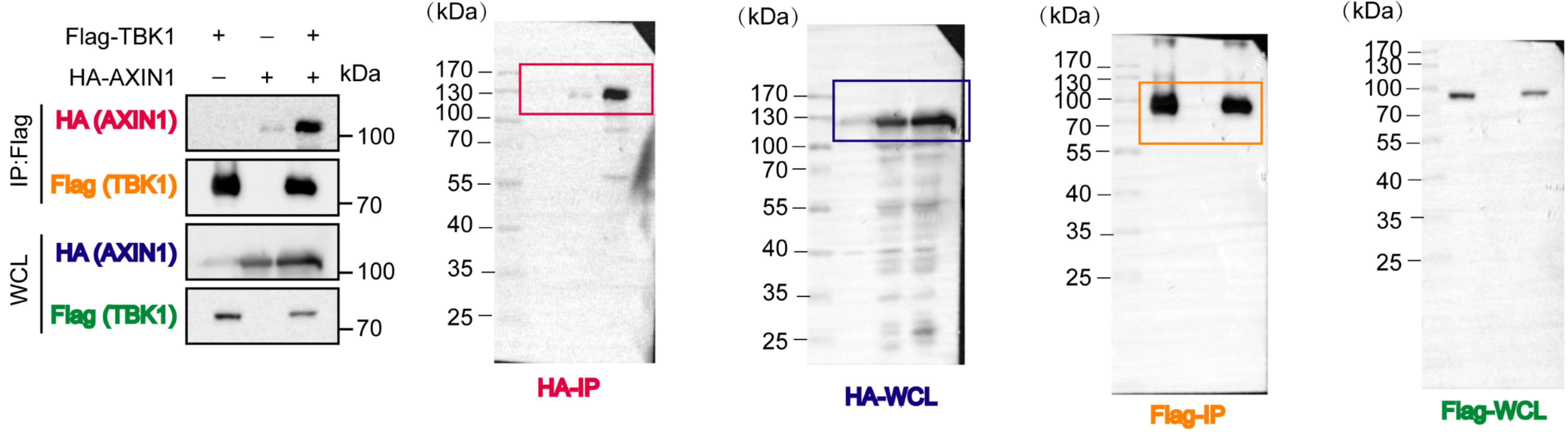

Source Fig.3j

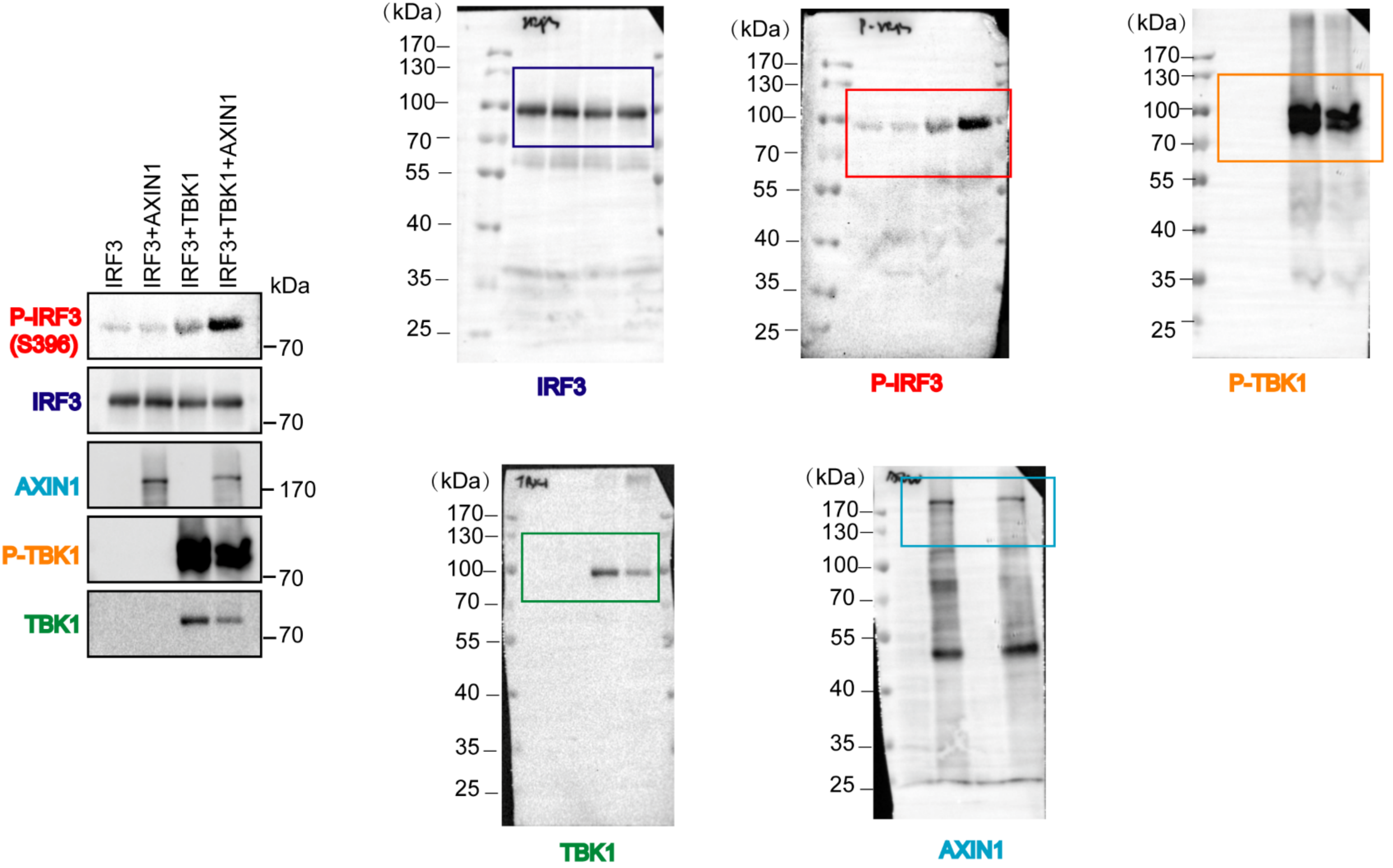

Source Fig.4h

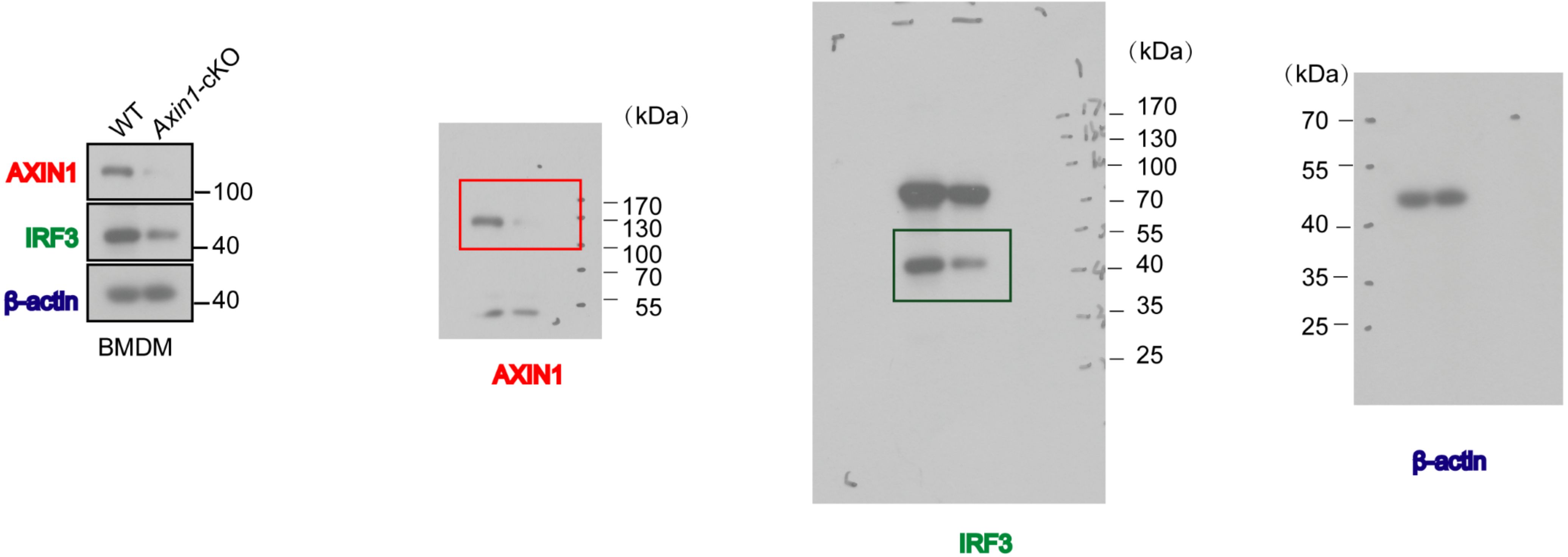

Source Fig.5g

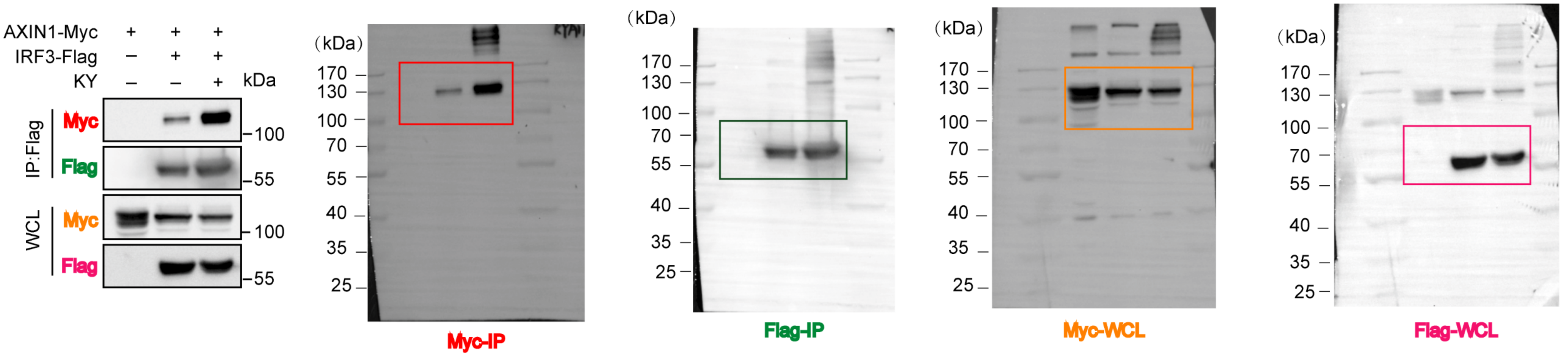

Source Fig.5h

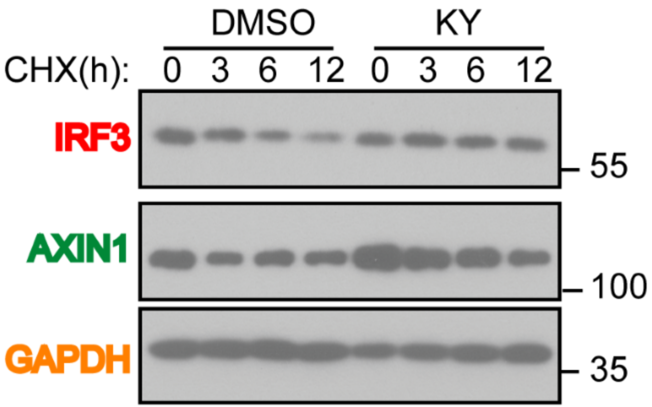

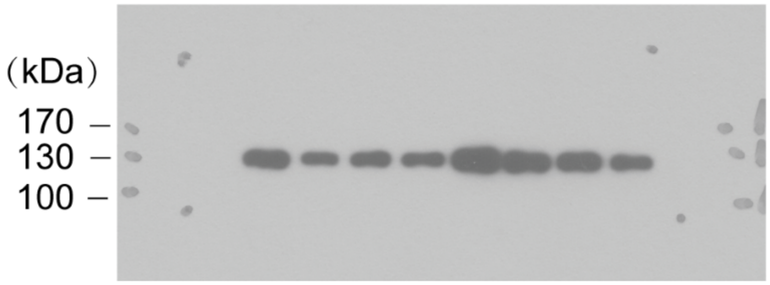

AXIN1

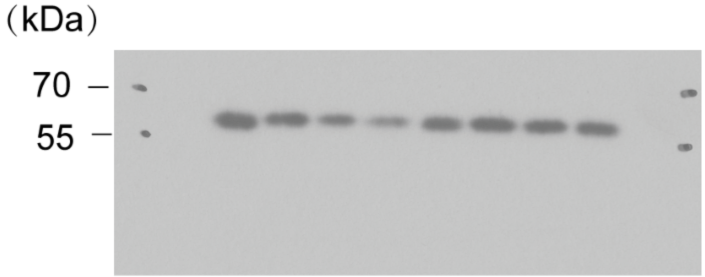

IRF3

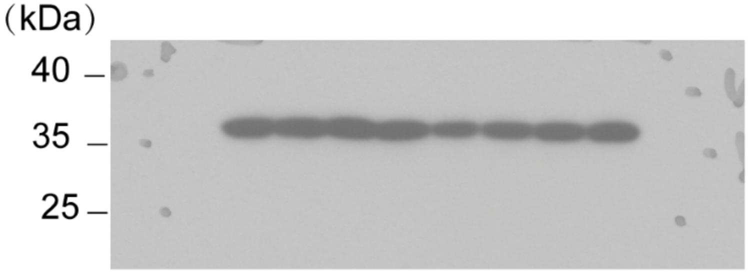

GAPDH

Source Fig. s1b

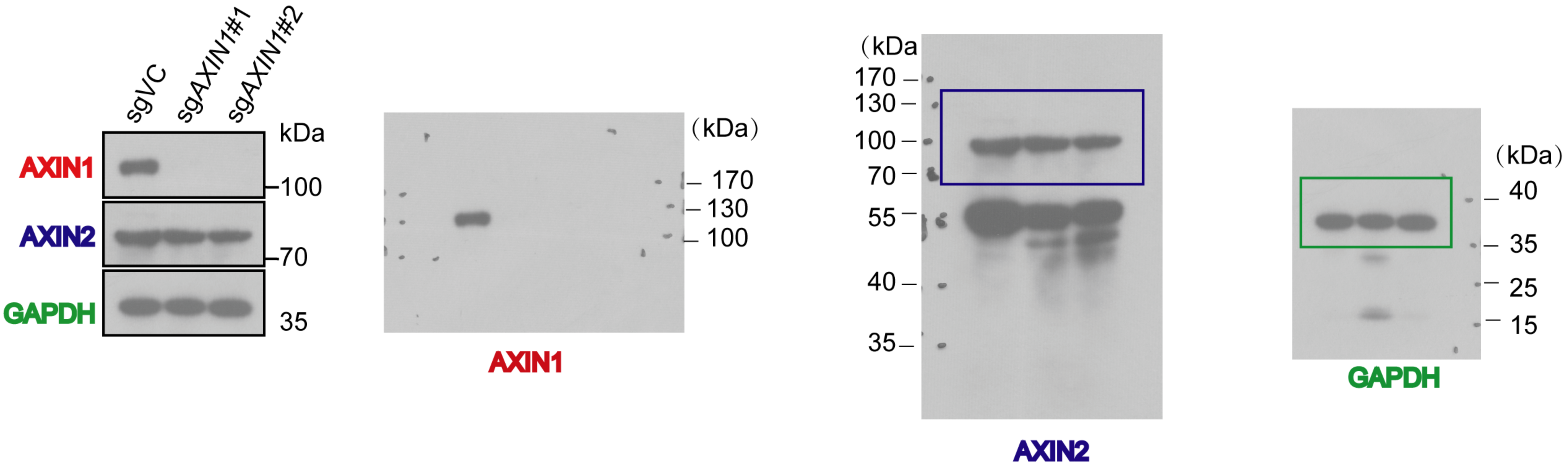

Source Fig. s1c

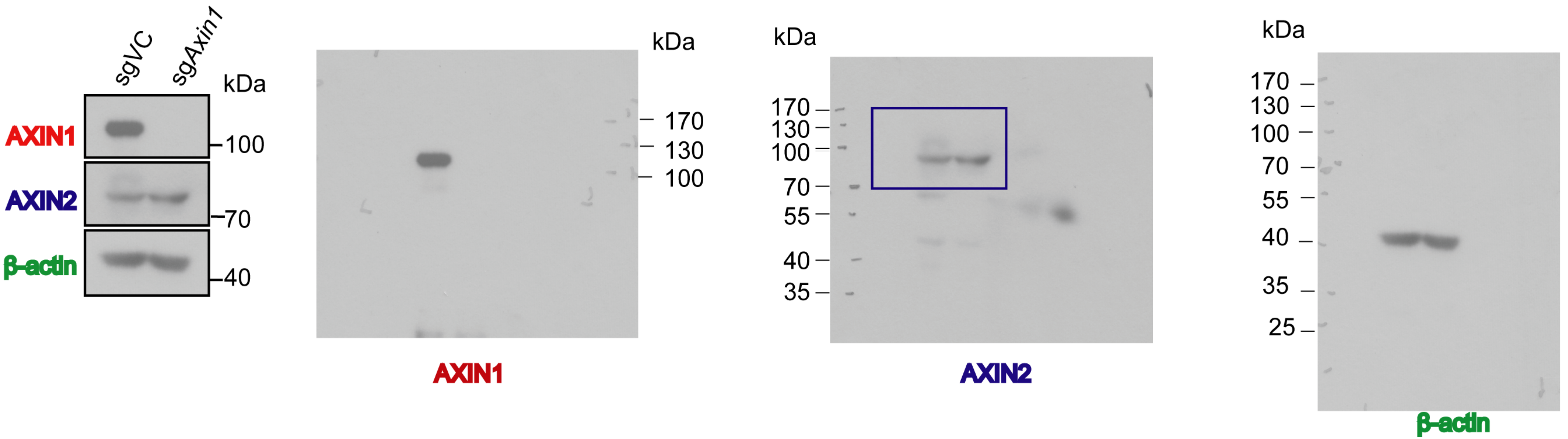

Source Fig. s2c

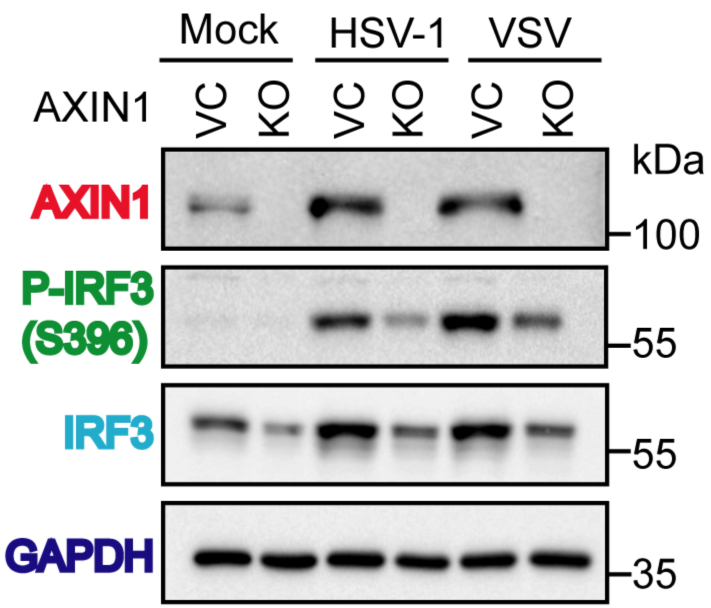

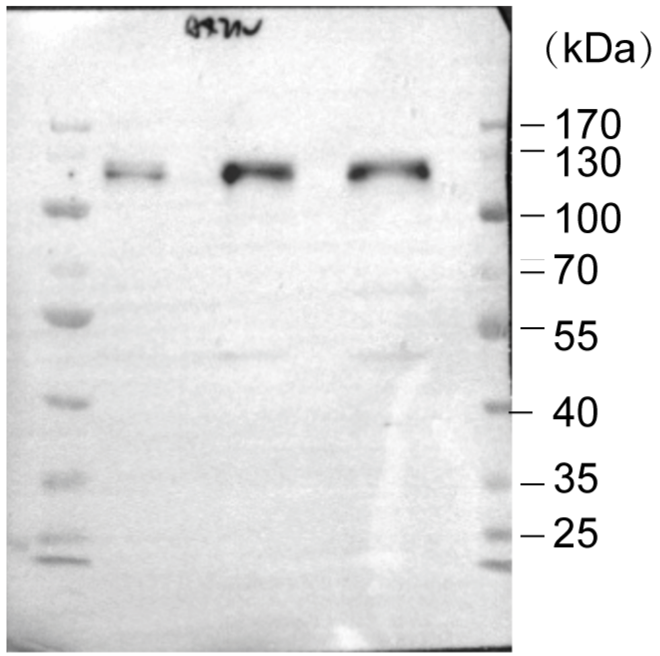

AXIN1

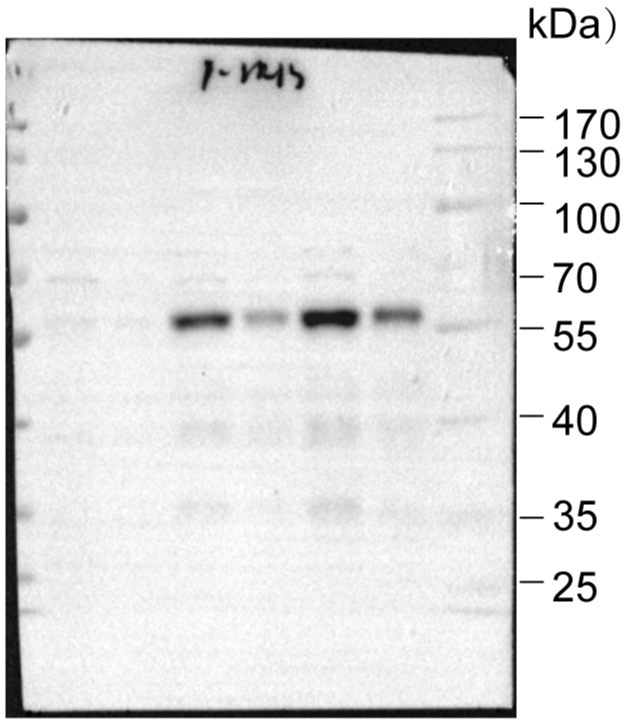

P-IRF3

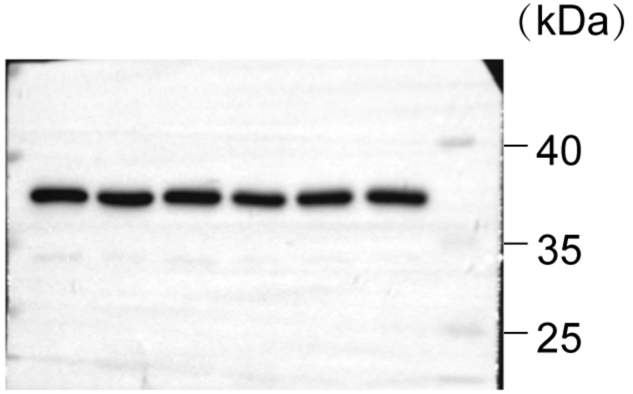

GAPDH

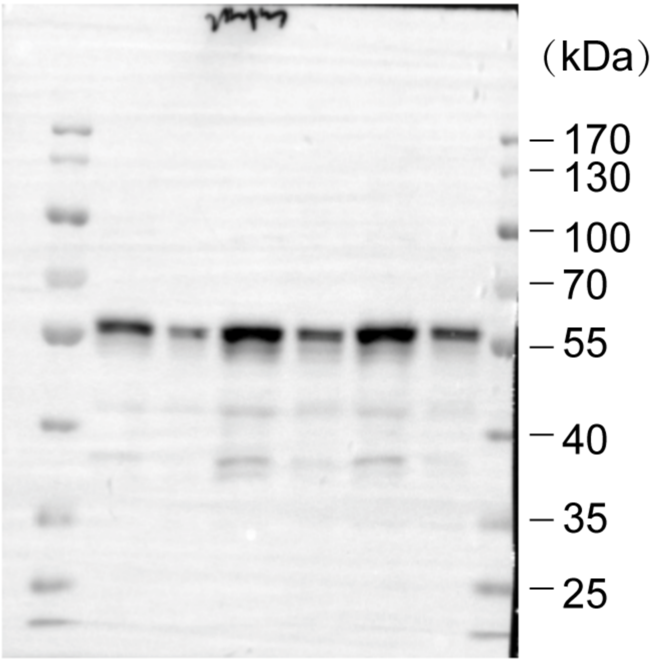

IRF3

Source Fig. s2d

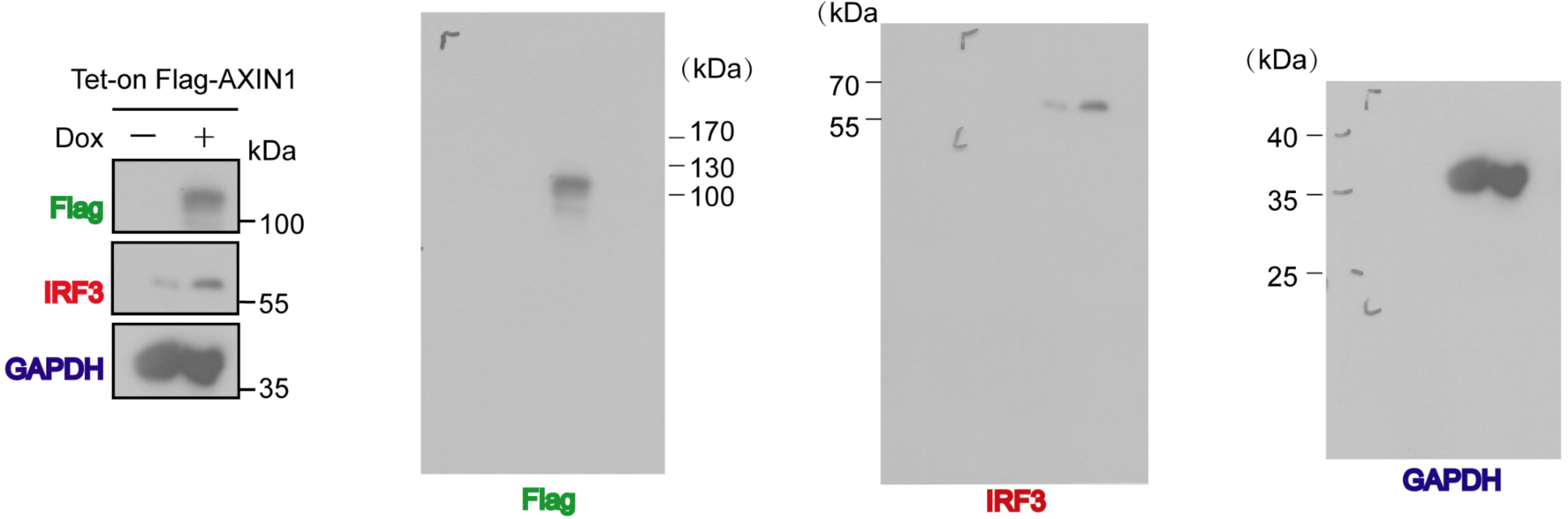

Source Fig. s2f

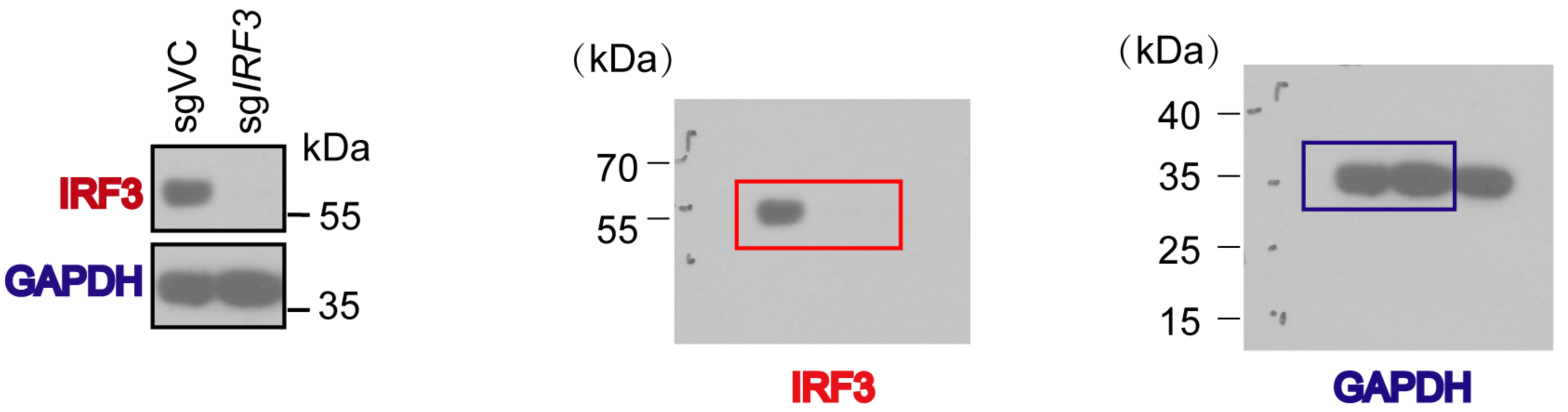

Source Fig. s3a

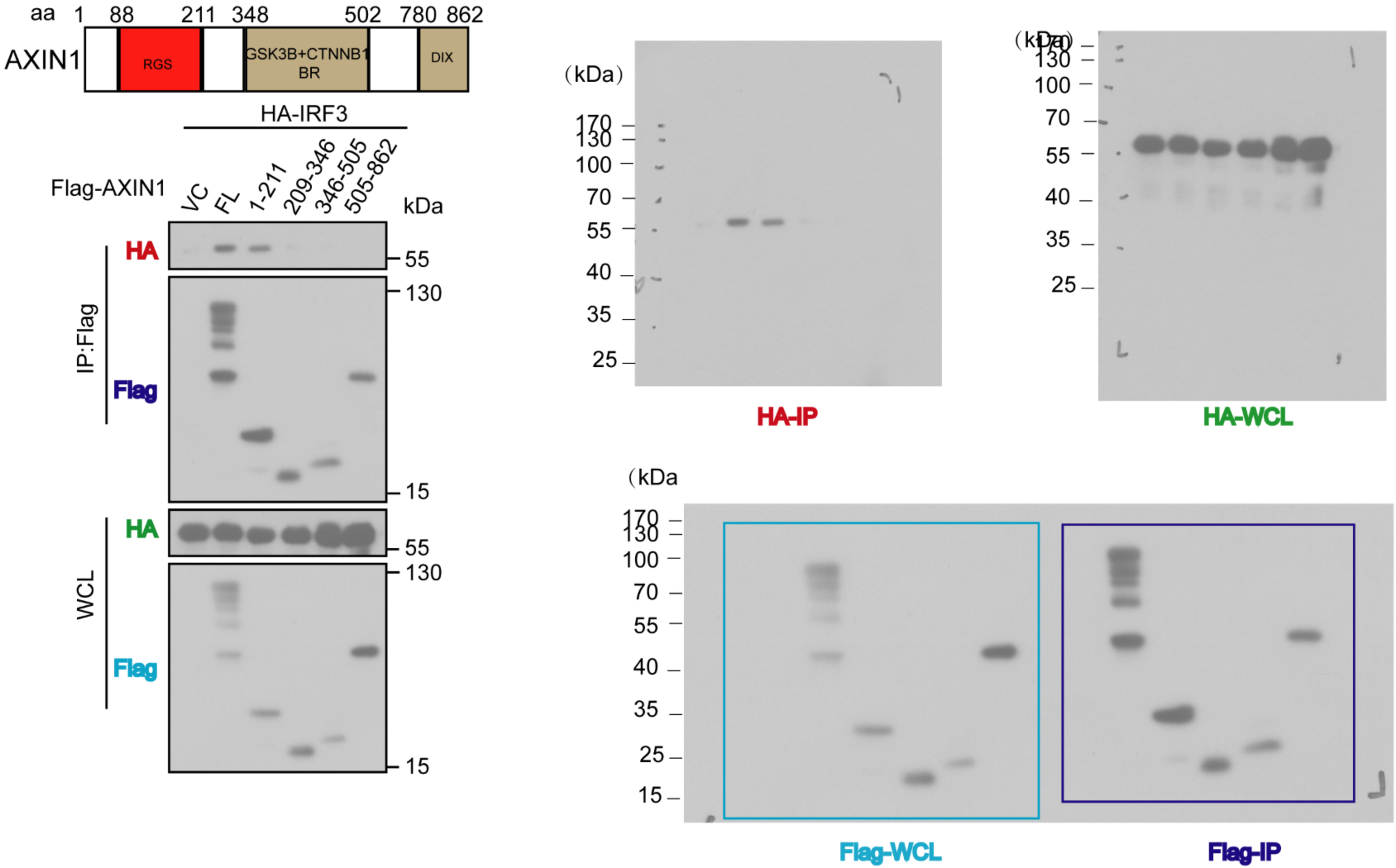

Source Fig. s3b

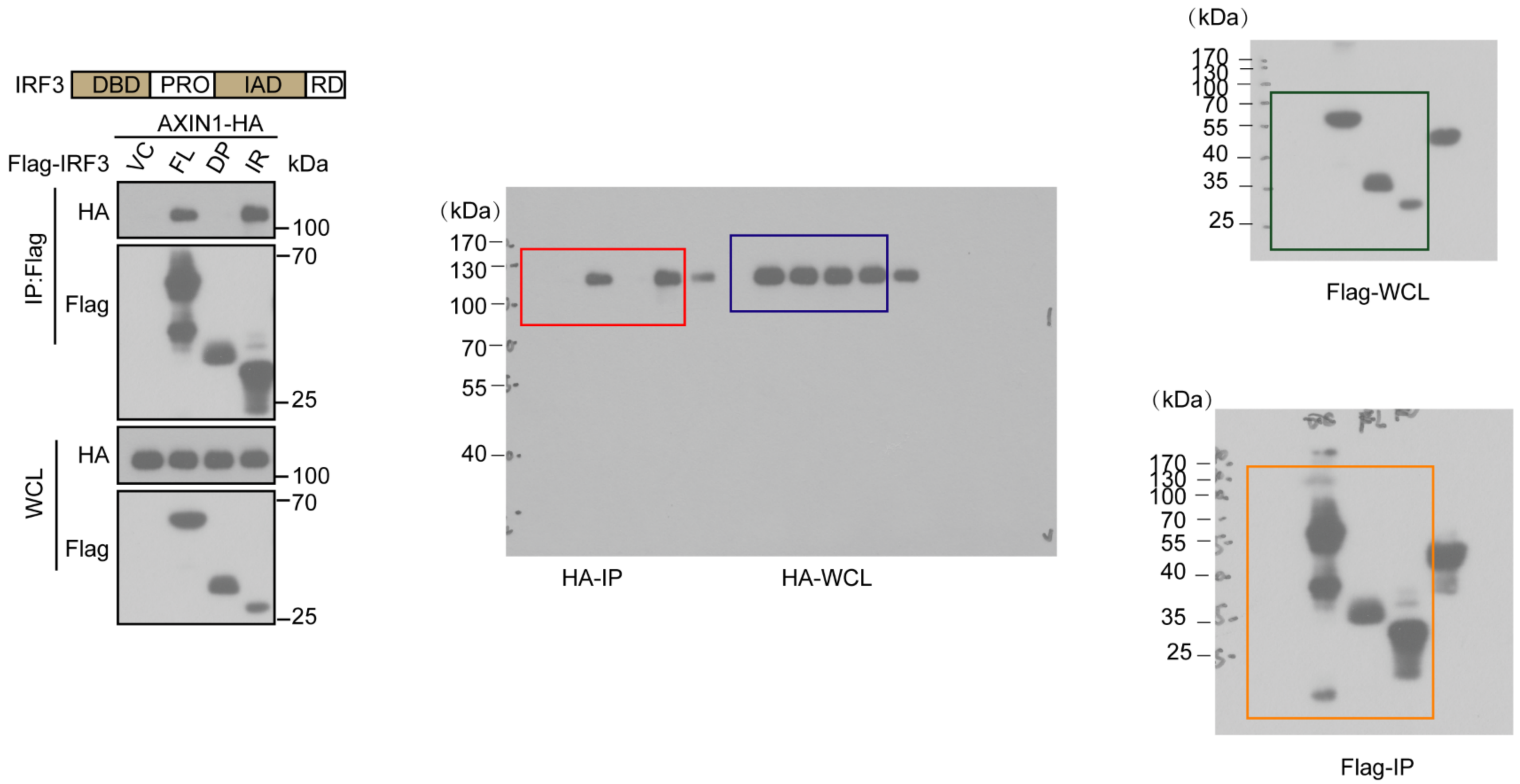

Source Fig. s4a

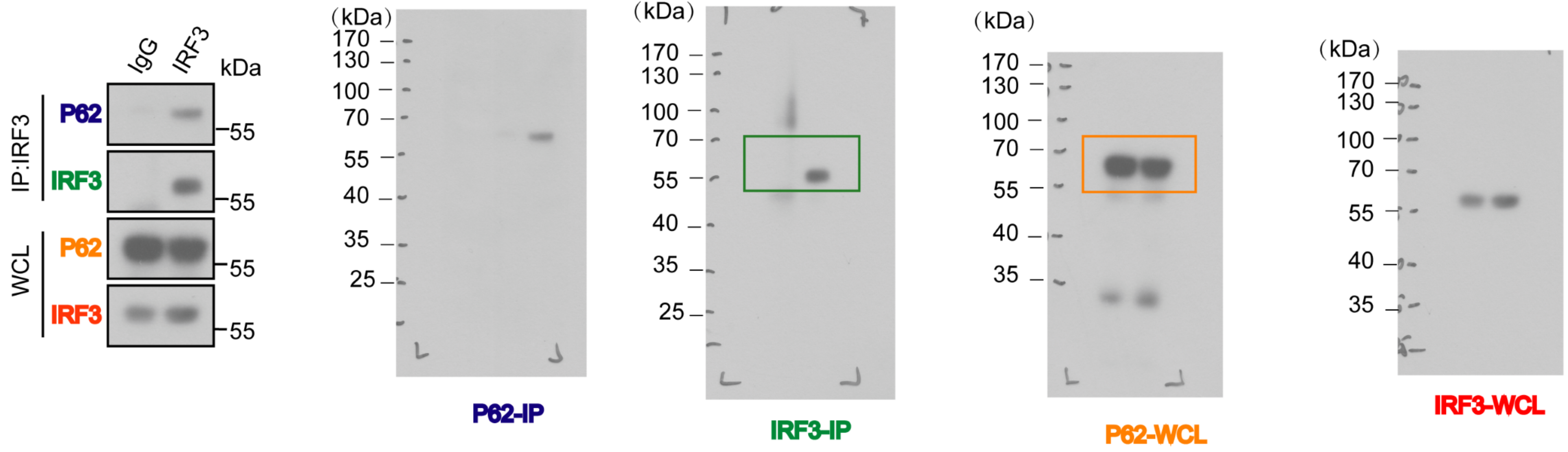

Source Fig. s4b

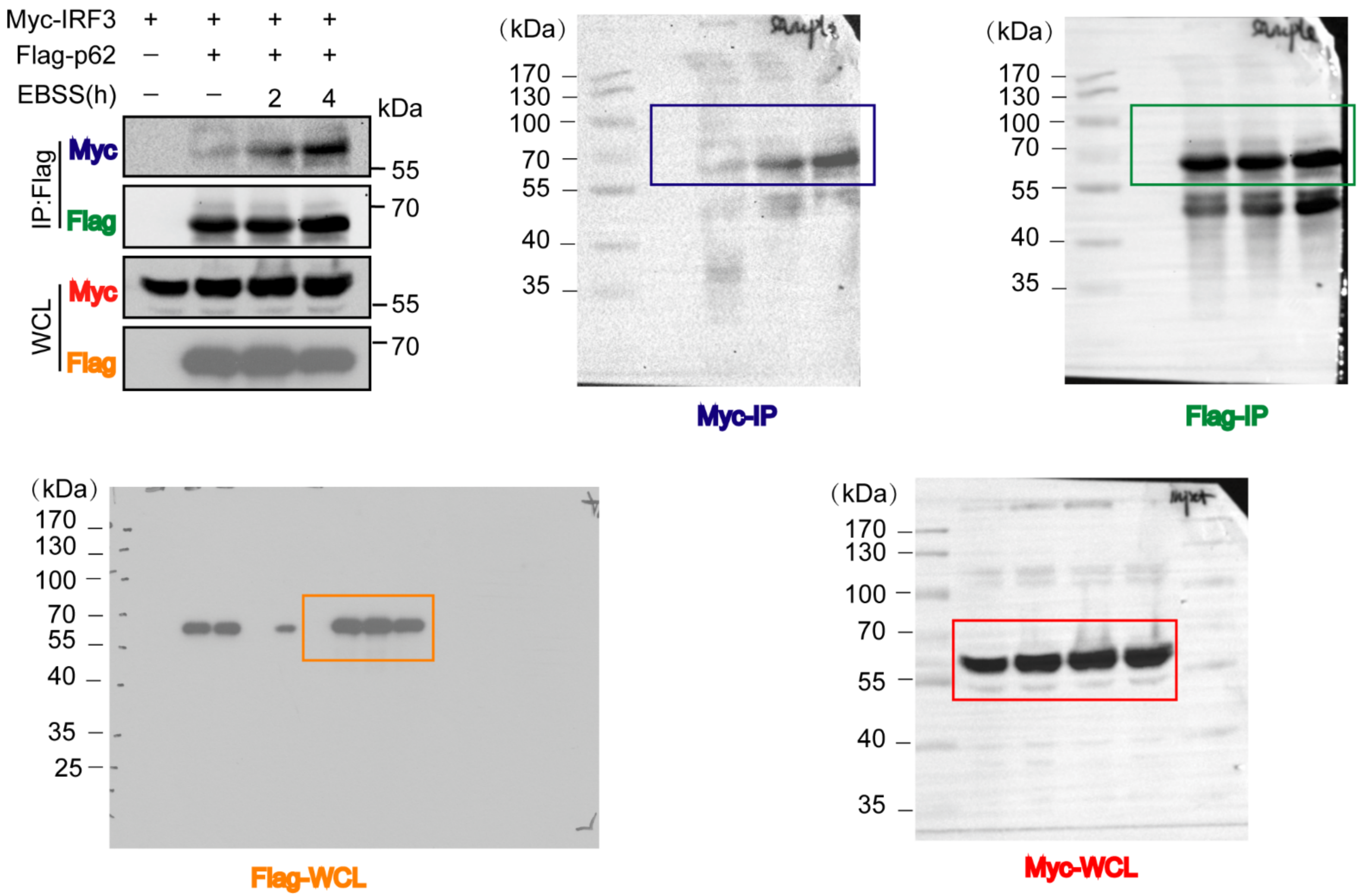

Source Fig. s4c

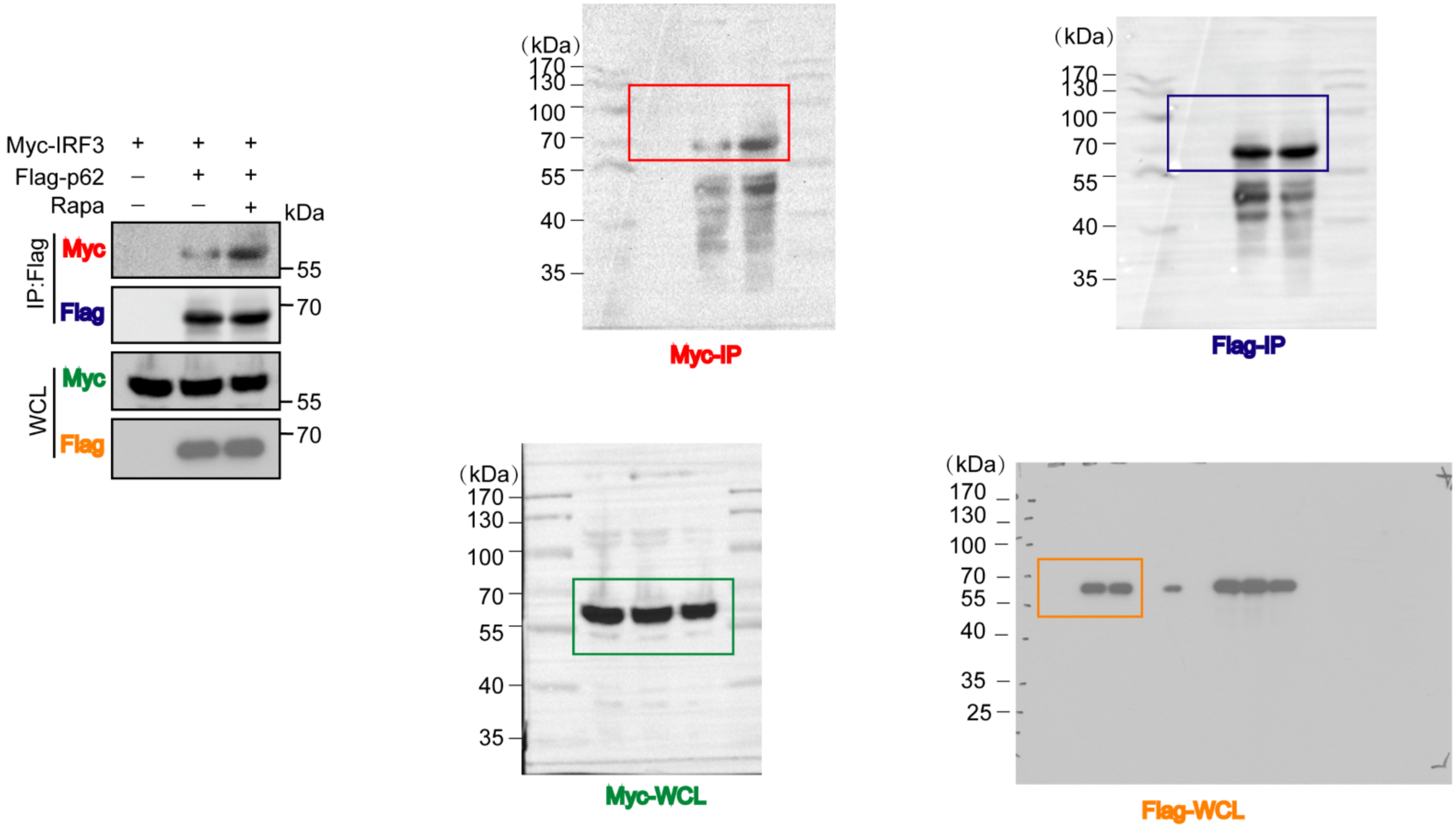

Source Fig. s5a

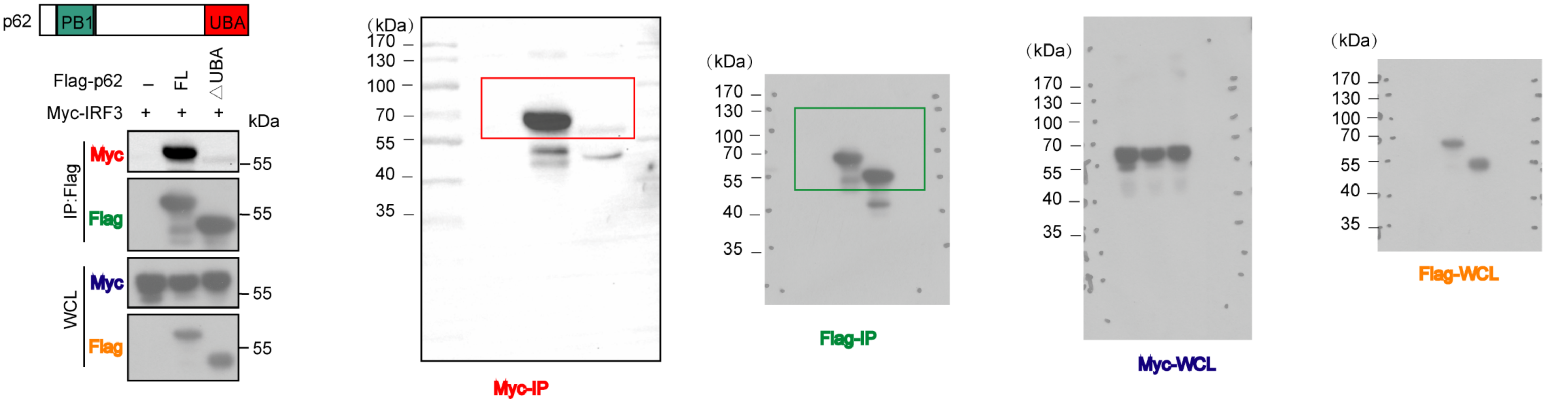

Source Extended Data Fig. 5b

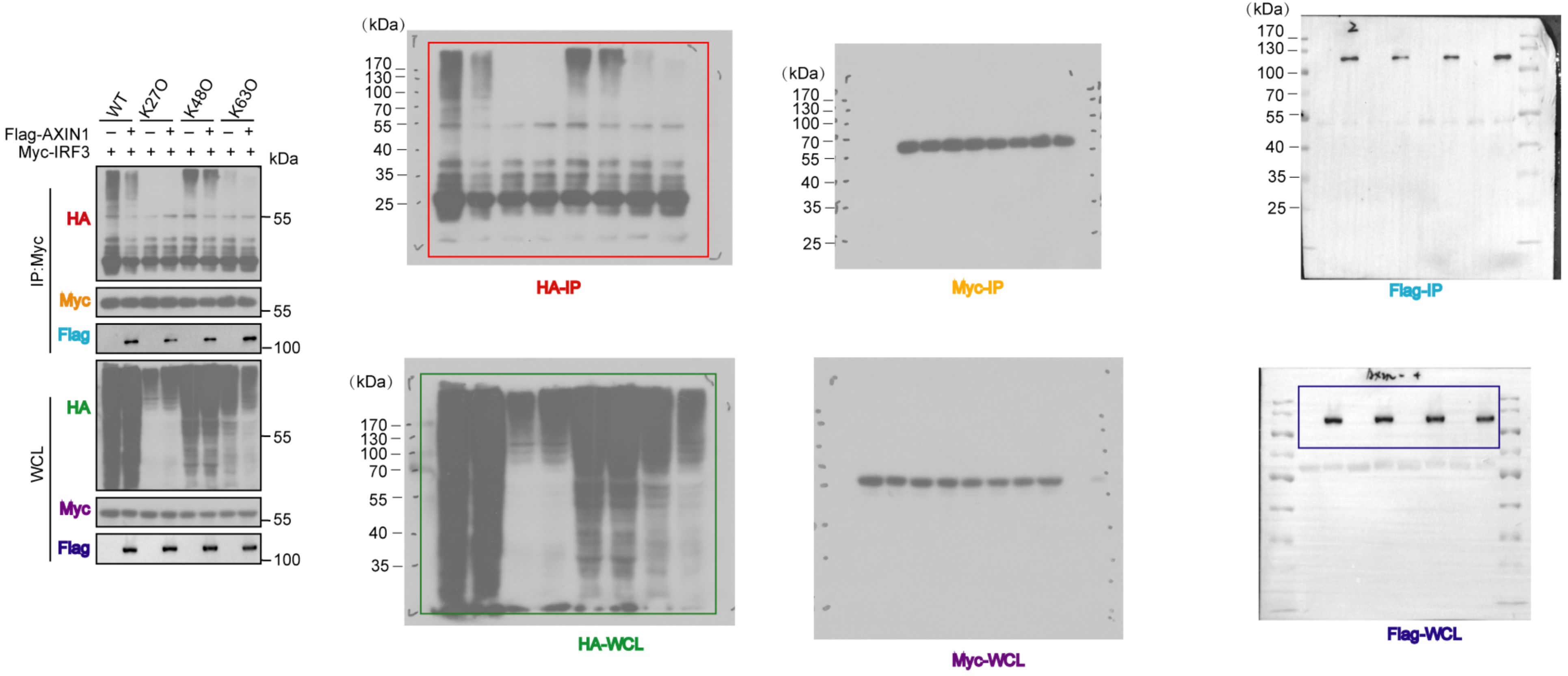

Source Fig. s5c

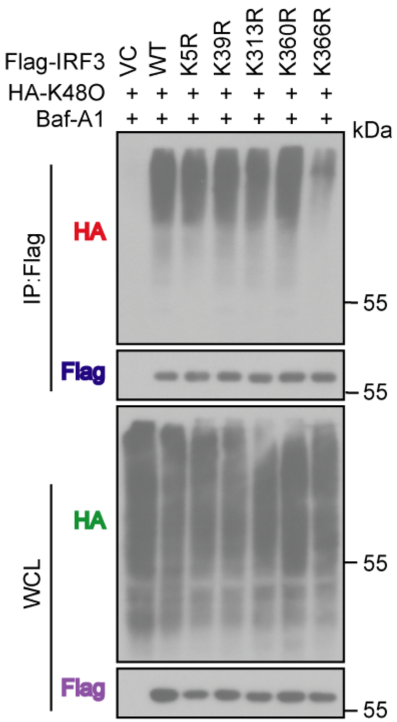

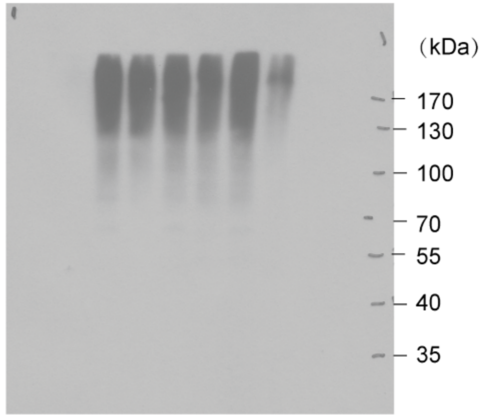

HA-IP

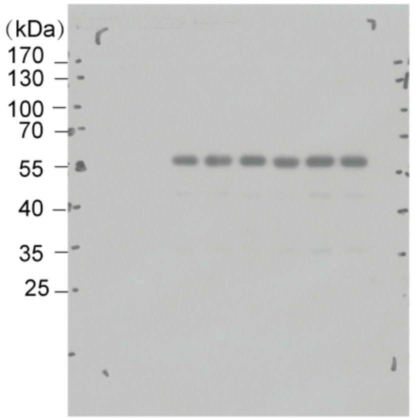

Flag-IP

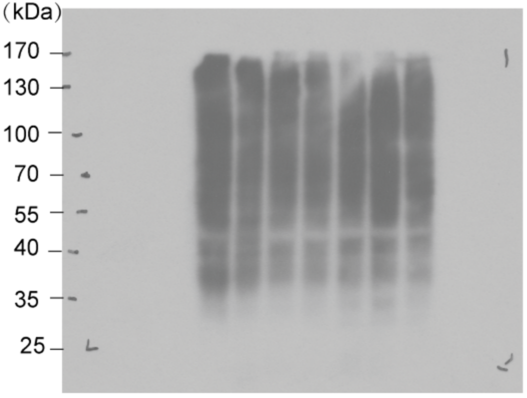

HA-WCL

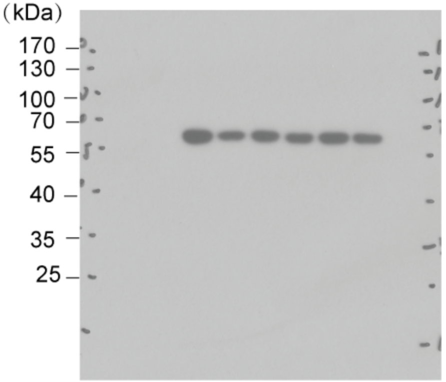

Flag-WCL

Source Fig. s5d

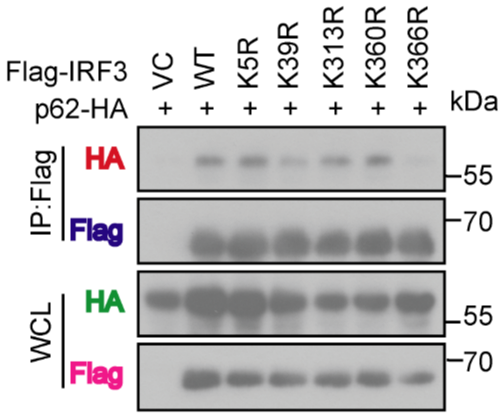

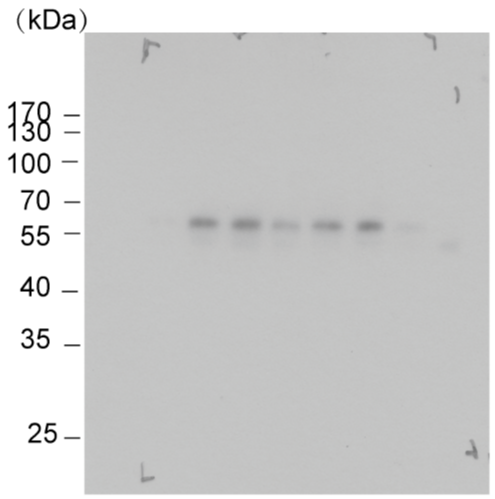

HA-IP

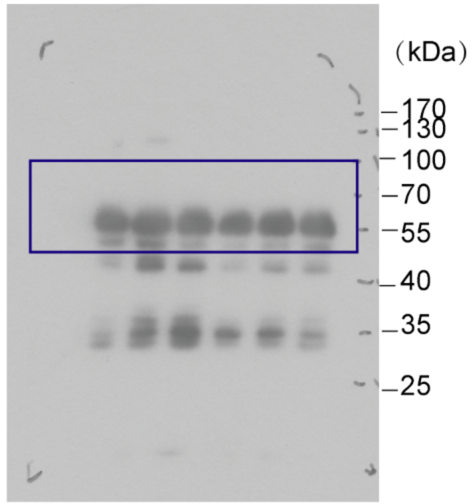

Flag-IP

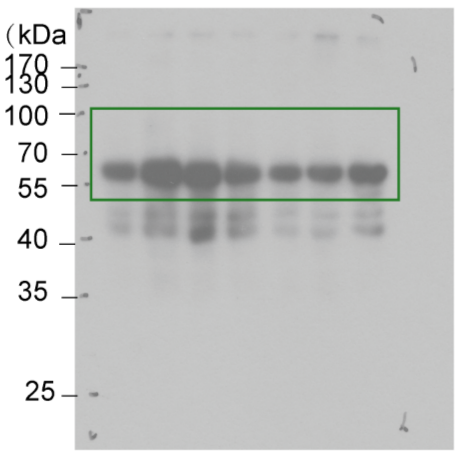

HA-WCL

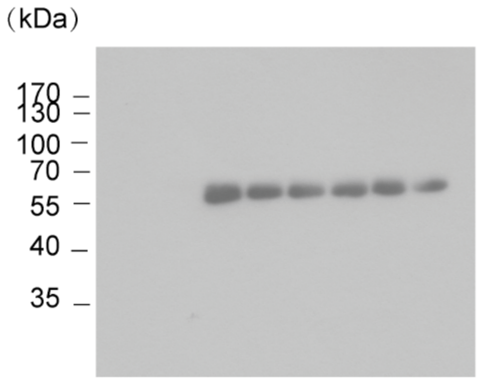

Flag-WCL

Source Fig. s6a

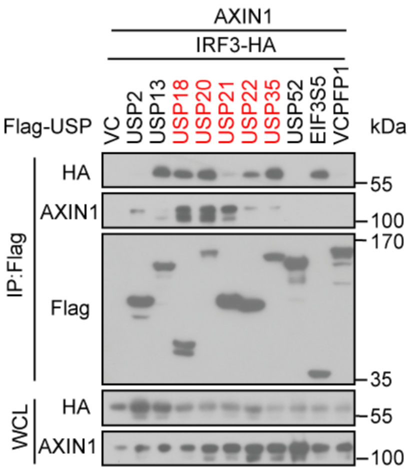

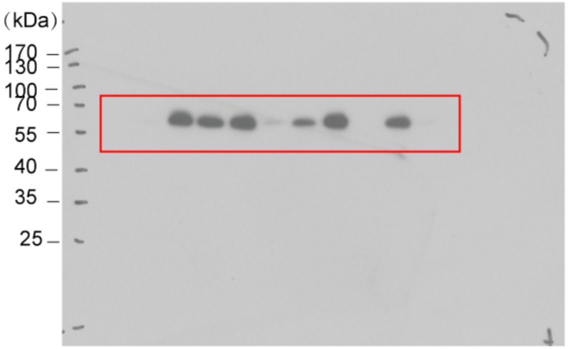

HA-IP

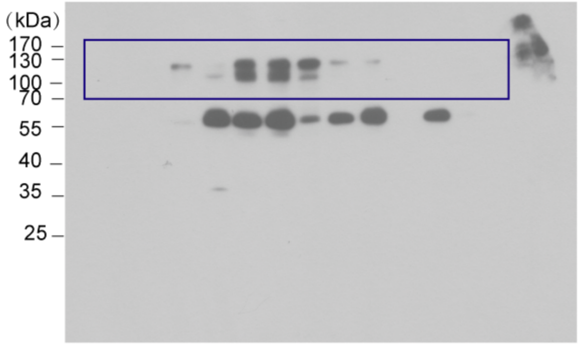

AXIN1-IP

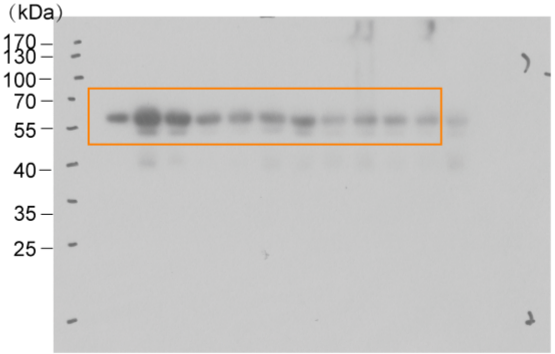

HA-WCL

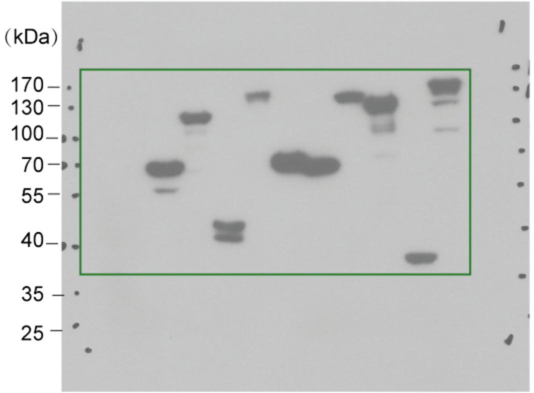

Flag-IP

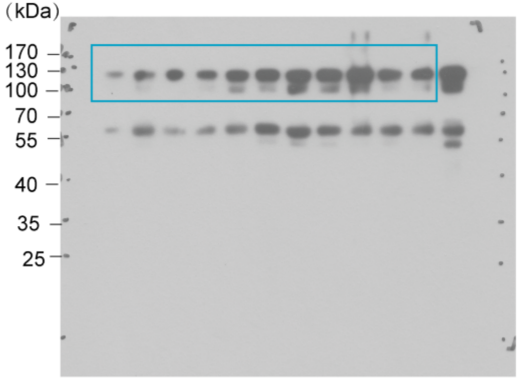

AXIN1-WCL

Source Fig. s6b

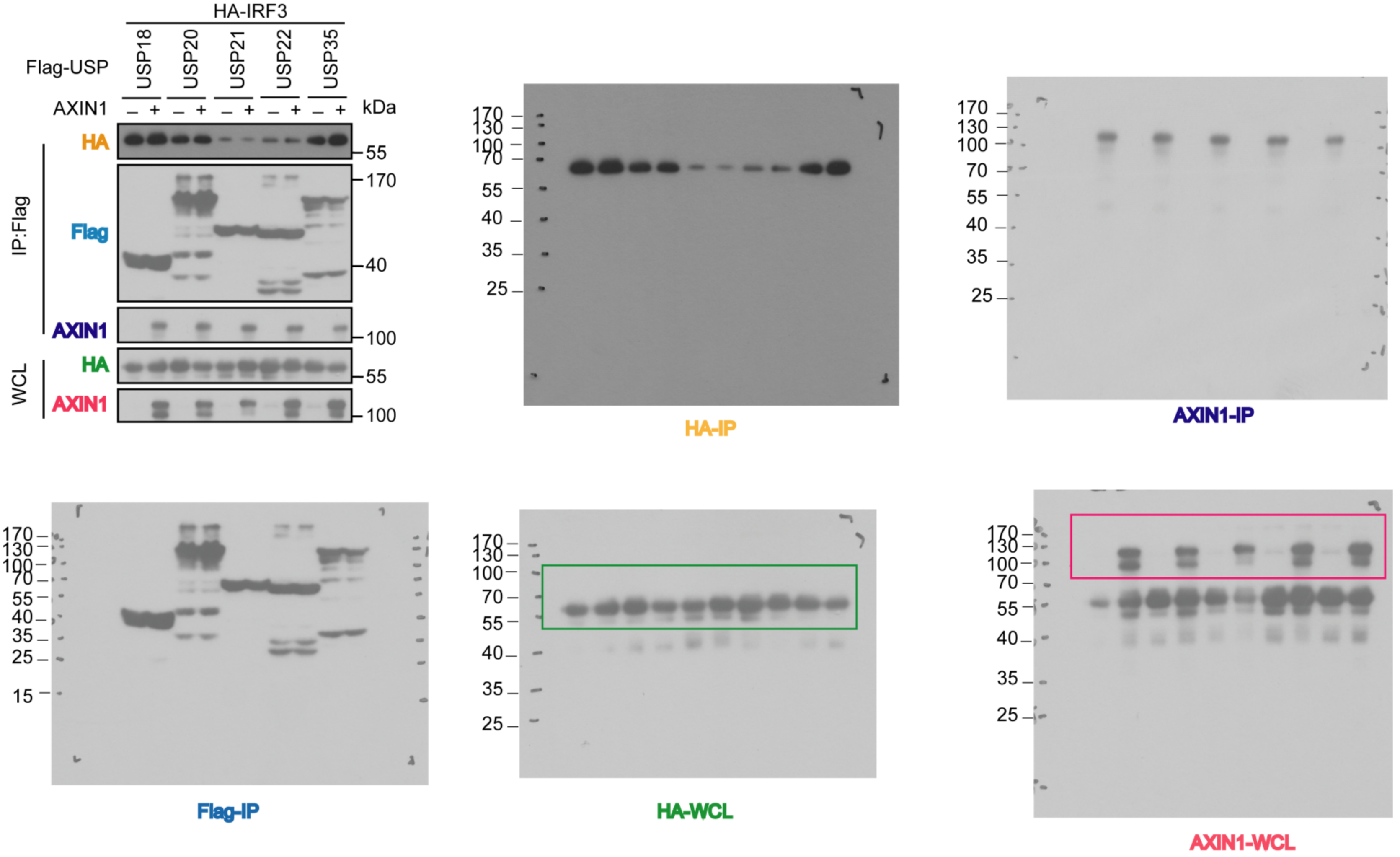

Source Fig. s6c

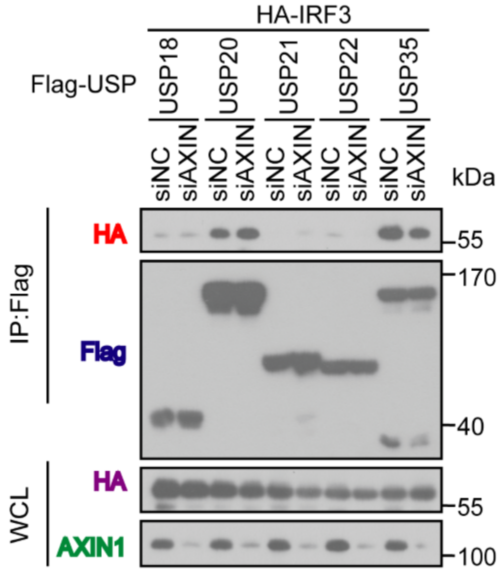

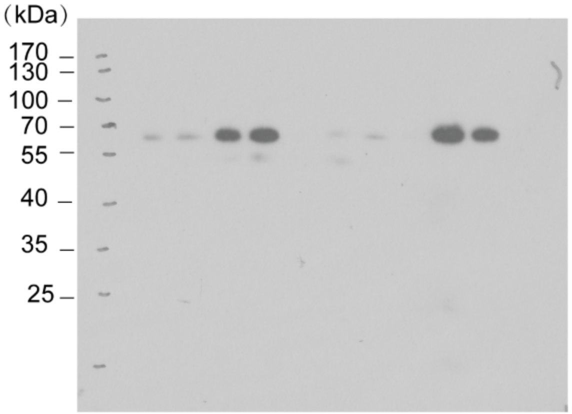

HA-IP

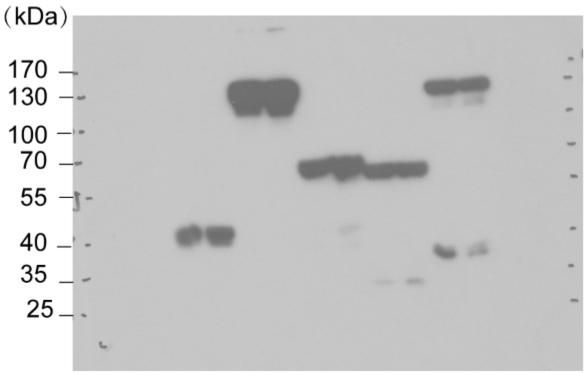

Flag-IP

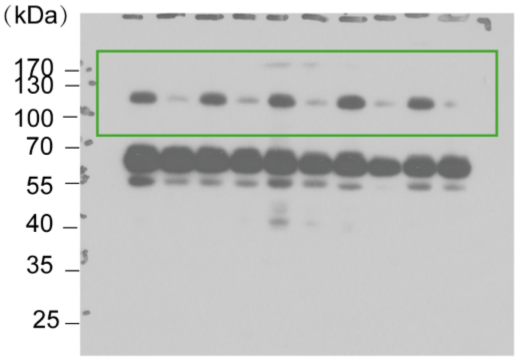

AXIN1-WCL

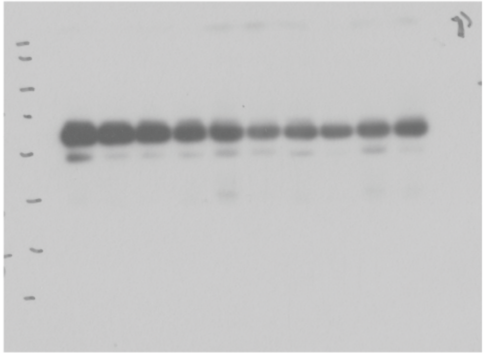

HA-WCL

Source Fig. s6d

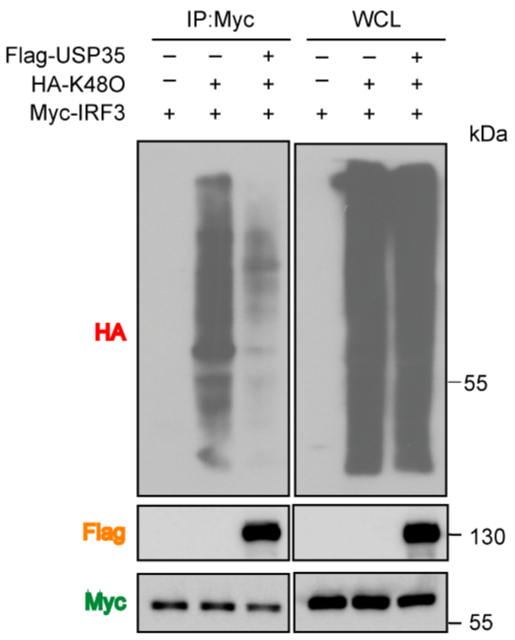

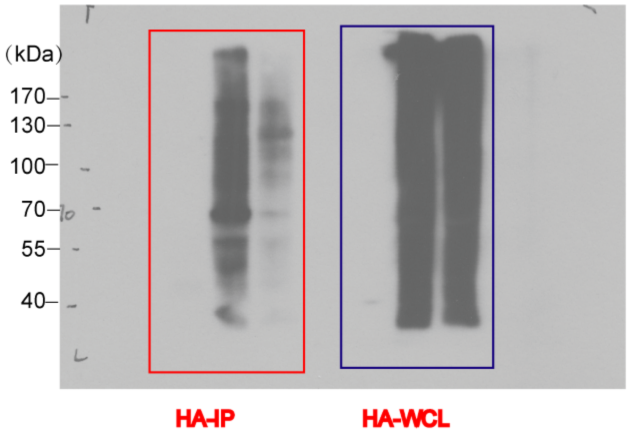

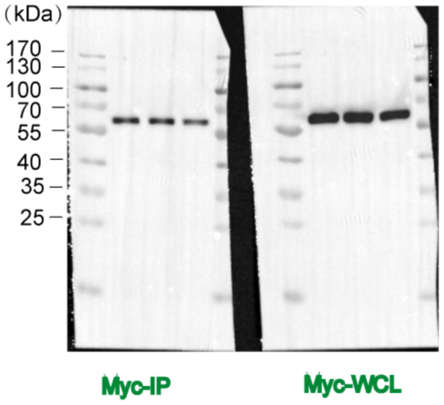

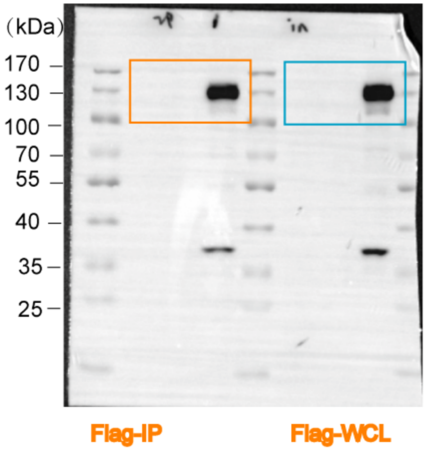

Source Fig. s6e

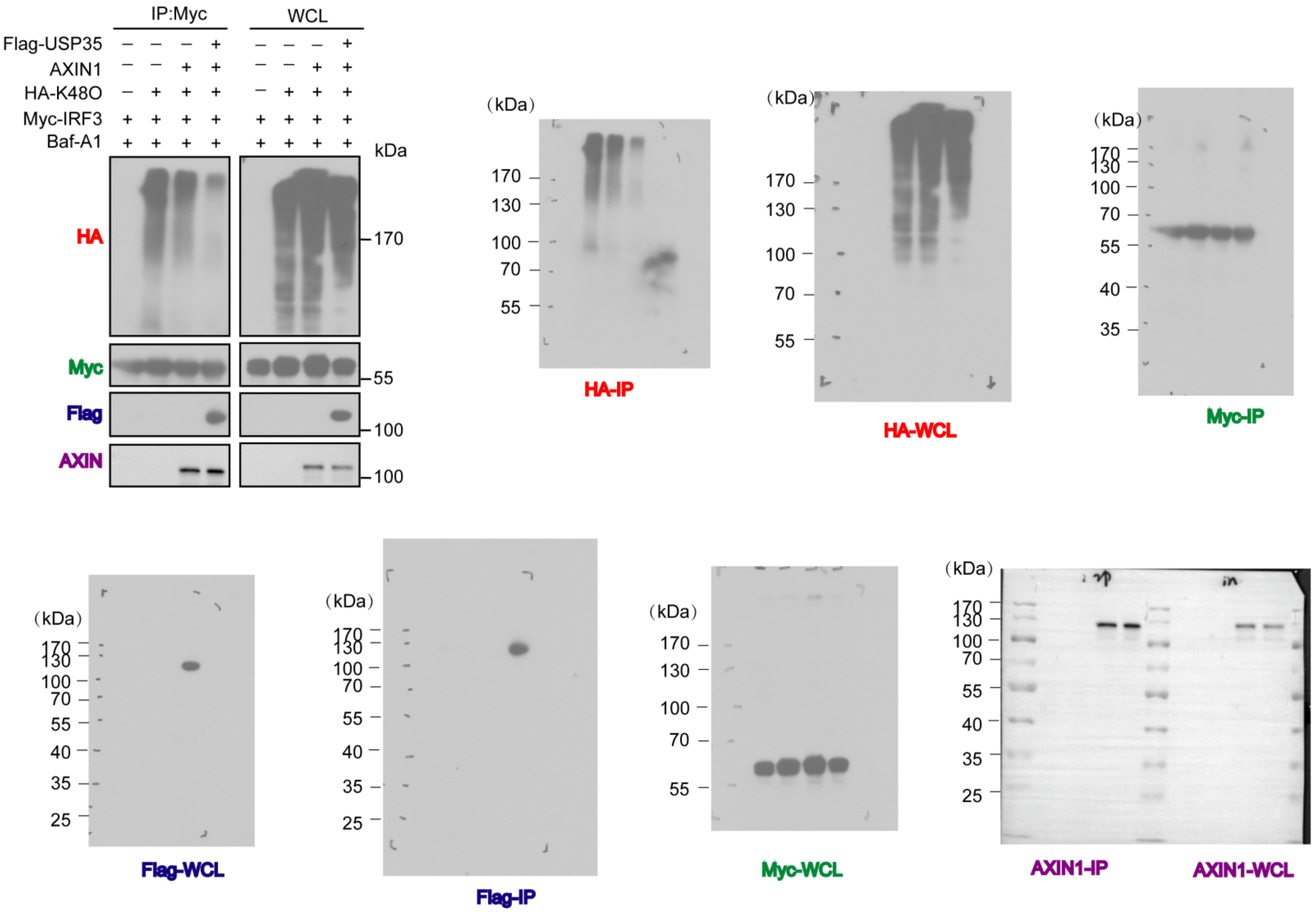

Source Fig. s6f

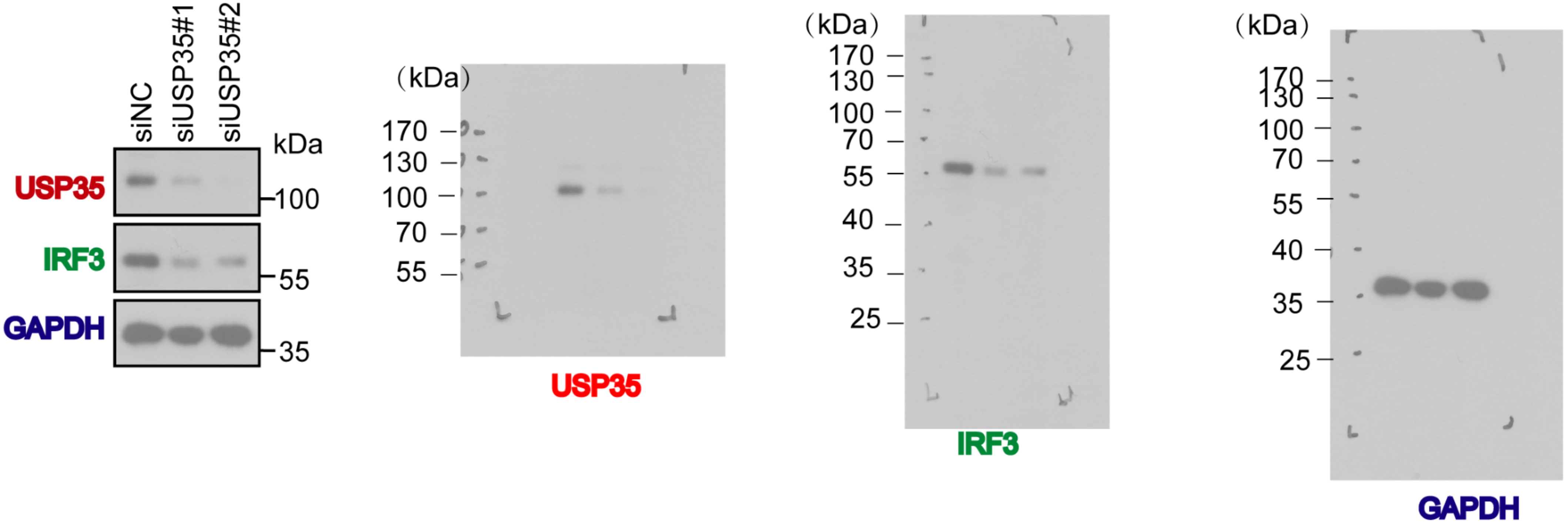

Source Fig. s7c

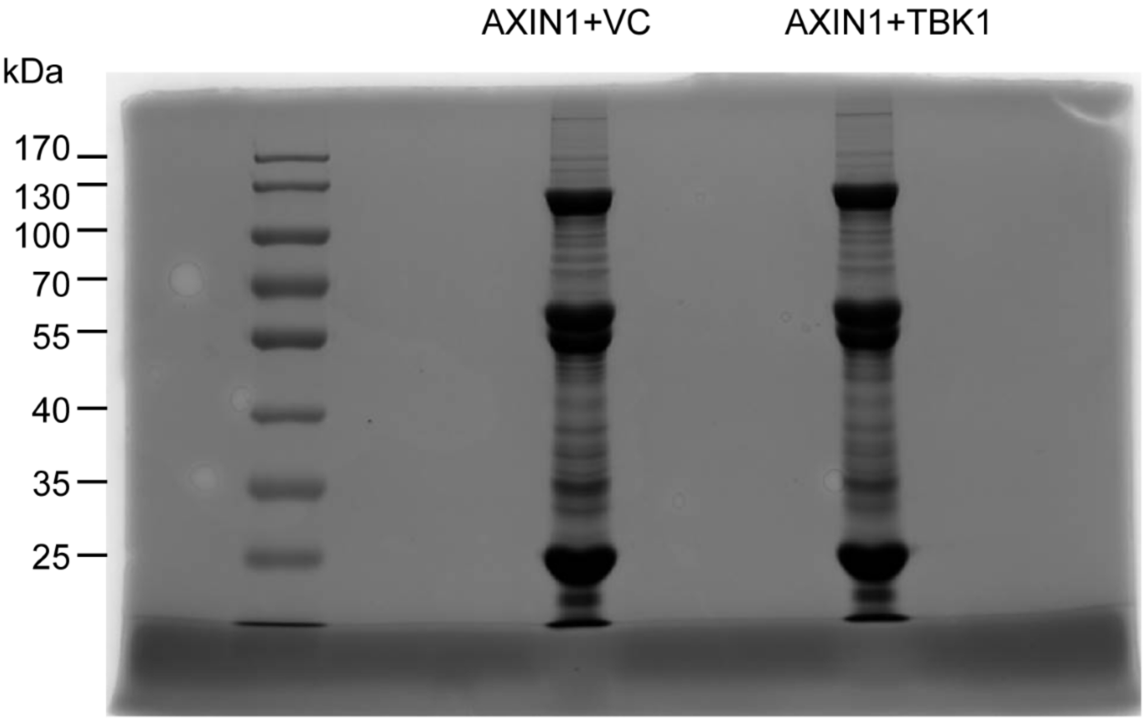

Source Fig. s7f

a

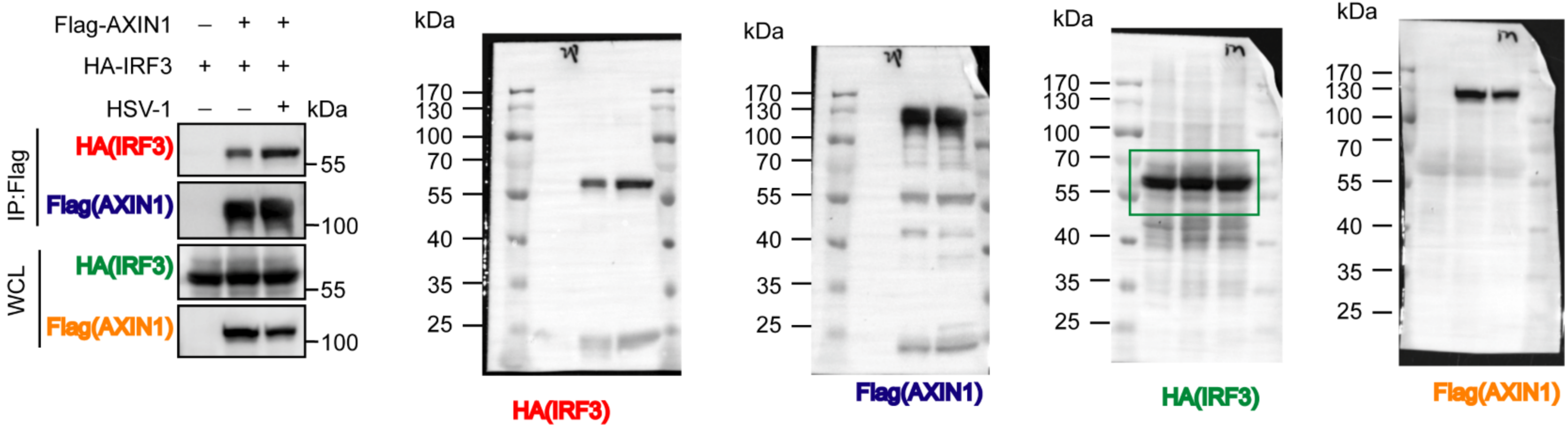

b

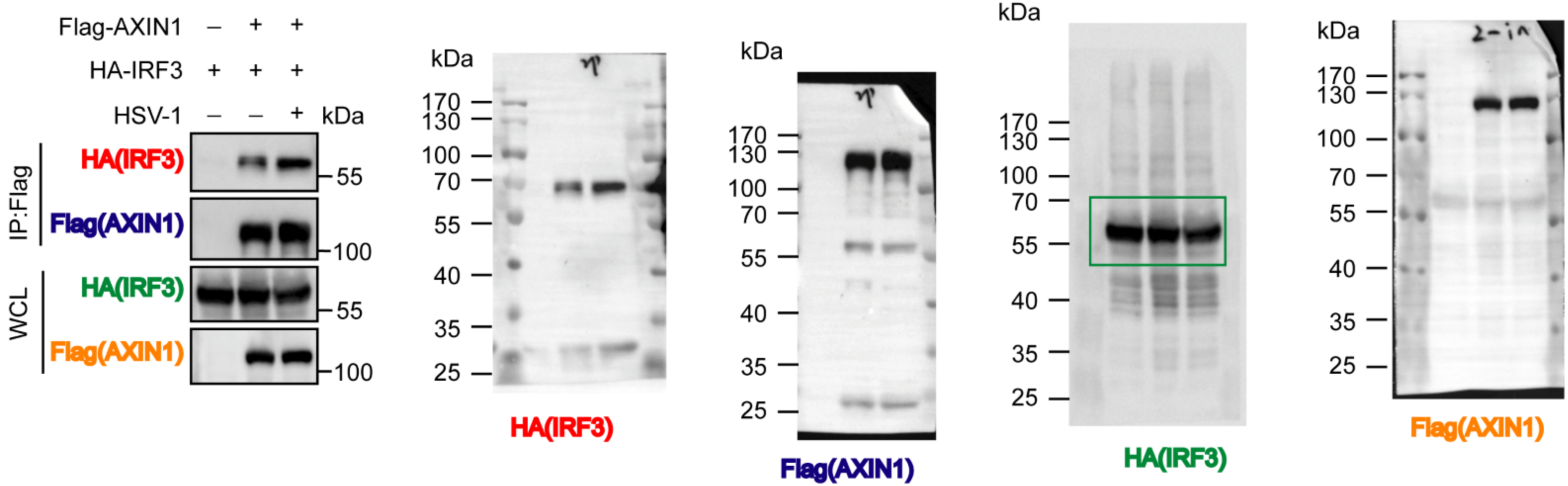

Source Fig. s8

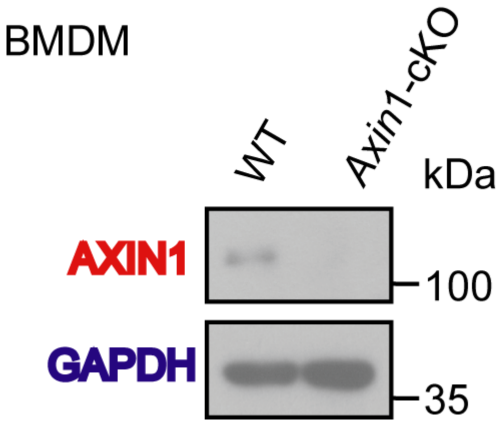

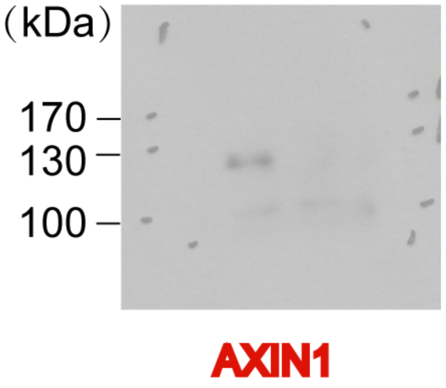

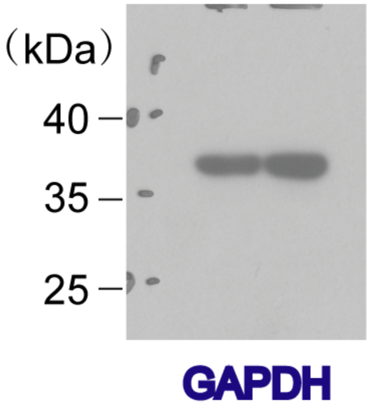

Source Extended Data Fig. 11a

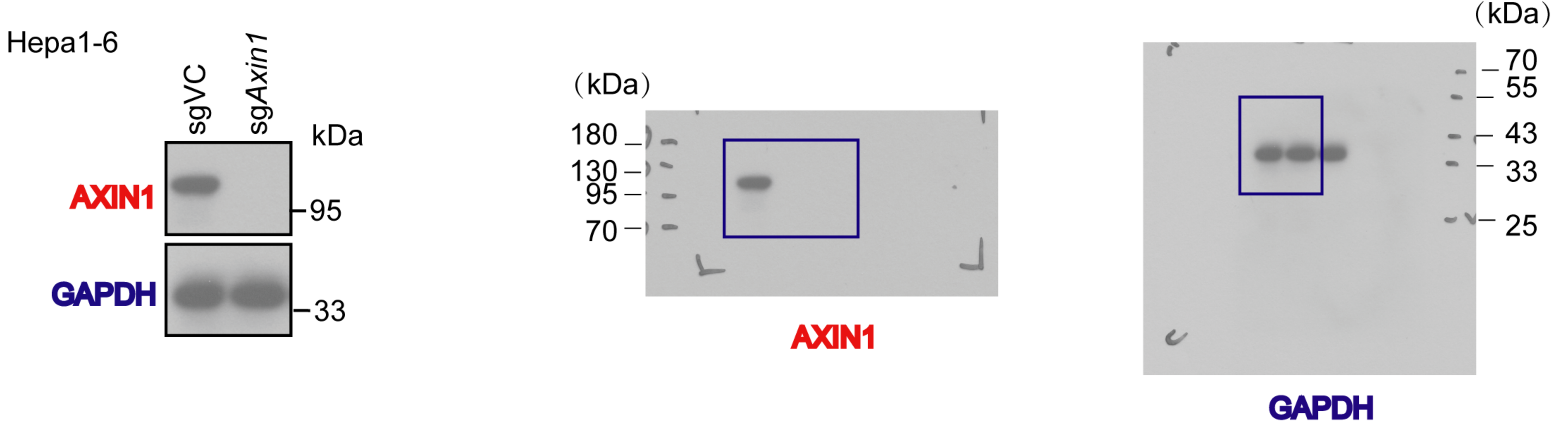

Supplement: Supplementary file 2 — Western blot source data [file 41392_2024_1978_MOESM2_ESM.pdf]
