# Supplementary figures and images for: AXIN1 boosts antiviral response through IRF3 stabilization and induced phase separation
Source: Signal Transduct Target Ther. 2024 Oct 9;9:281. doi: 10.1038/s41392-024-01978-y (PMC11464762; doi:10.1038/s41392-024-01978-y)

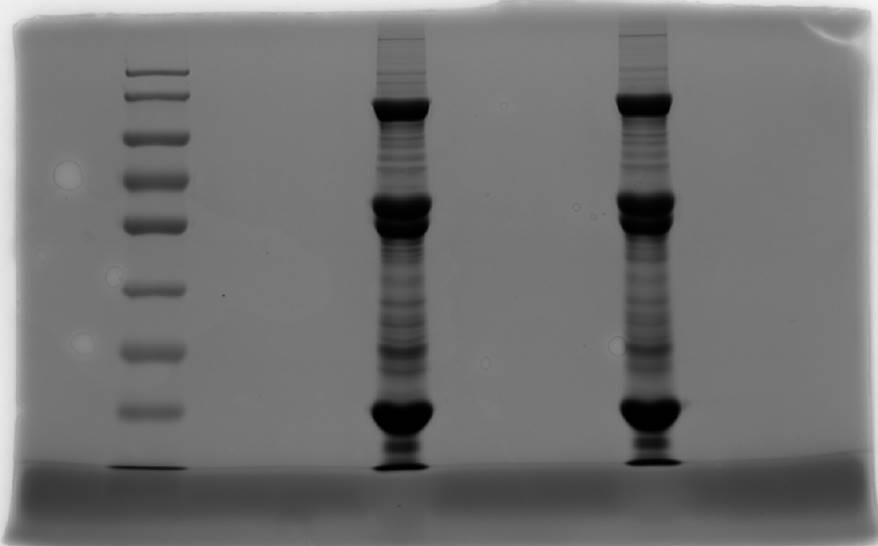

Supplement: Supplementary file 3 — Dataset-AI-proof-figures [file 41392_2024_1978_MOESM3_ESM.zip › AI-proof-reference/Figure s7c-Page #35, Match #22-AXIN-TBK1磷酸化考染图.jpg]

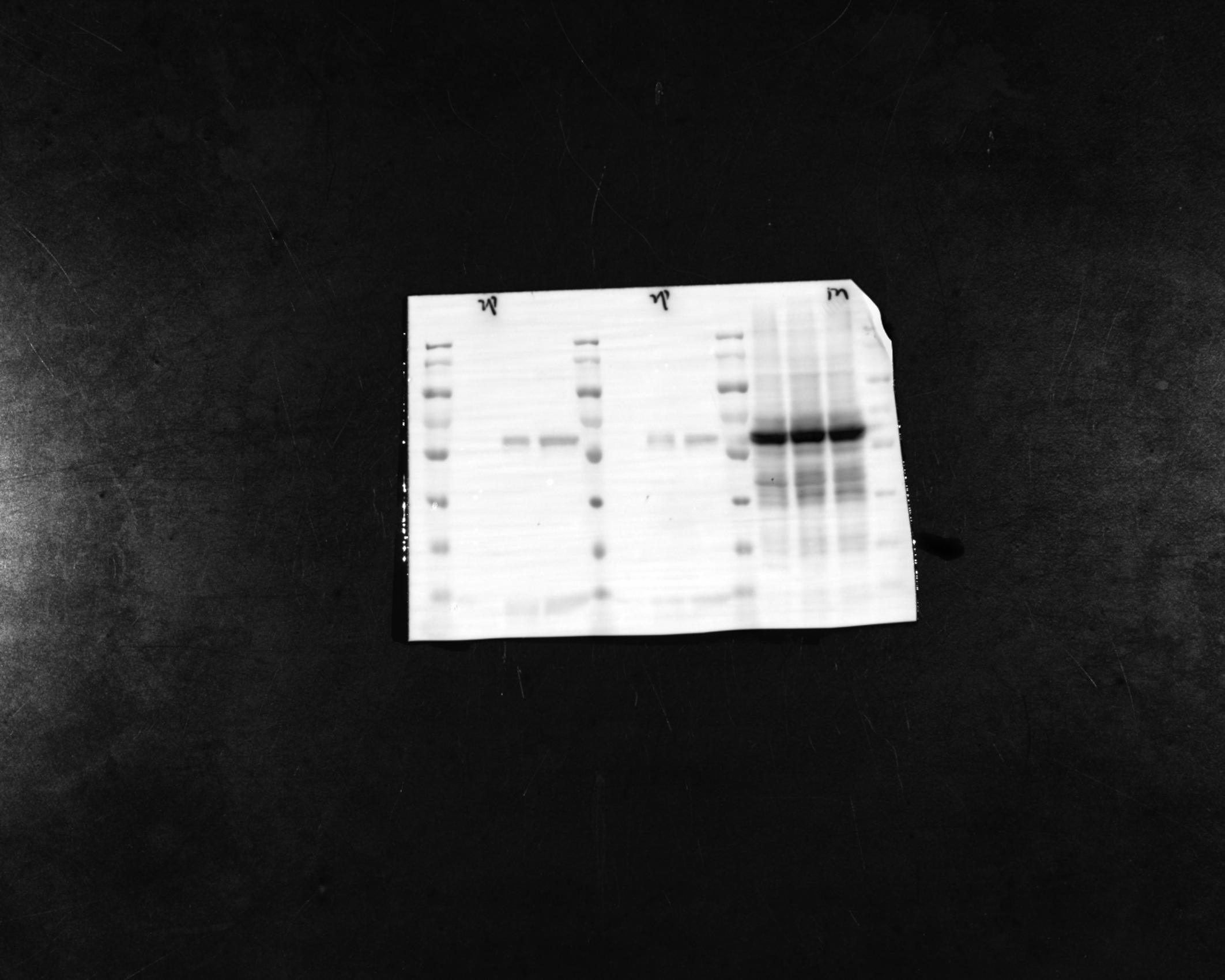

Supplement: Supplementary file 3 — Dataset-AI-proof-figures [file 41392_2024_1978_MOESM3_ESM.zip › AI-proof-reference/Figure S7f-Page #36, Match #23-HSV-input-HA-R(IRF3)-marker.tif]

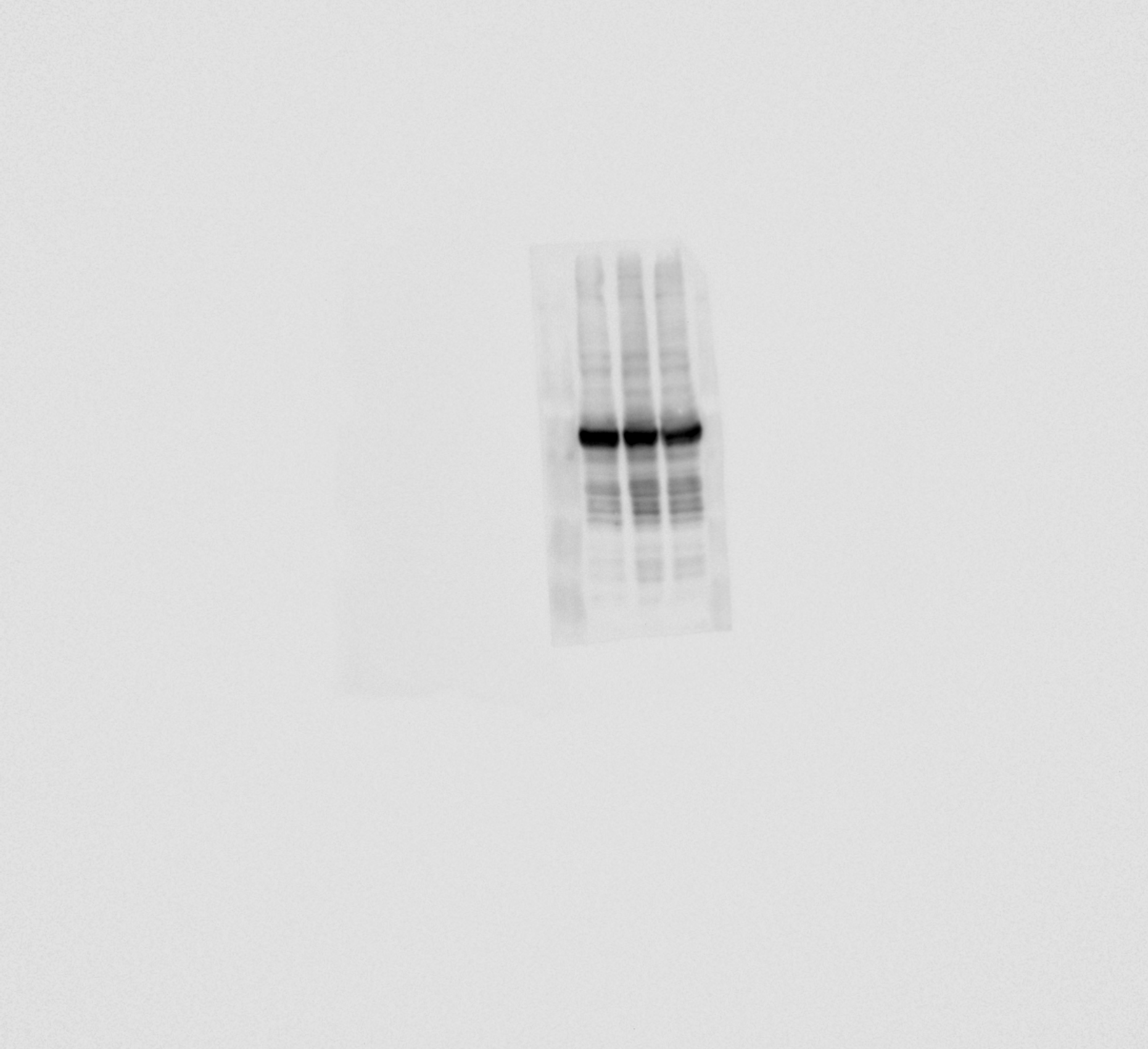

Supplement: Supplementary file 3 — Dataset-AI-proof-figures [file 41392_2024_1978_MOESM3_ESM.zip › AI-proof-reference/Figure S7f-Page #36, Match #23-VSV-IRF3-Input-HA.tif]
